# Age- and sex-related variations in extracellular vesicle profiling for the assessment of cardiovascular risk: the *EVaging* index

**DOI:** 10.1038/s41514-024-00189-7

**Published:** 2024-12-19

**Authors:** Jacopo Burrello, Jessica Goi, Alessio Burrello, Elena Vacchi, Azucena Rendon-Angel, Edoardo Lazzarini, Giovanni Bianco, Vittorio Limongelli, Giuseppe Vassalli, Carlo W. Cereda, Silvia Monticone, Paolo Mulatero, Benedetta Bussolati, Andrea Alimonti, Giovanni G. Camici, Giorgia Melli, Elena Osto, Giovanni Pedrazzini, Barile Lucio

**Affiliations:** 1 Cardiovascular Theranostics, Istituto Cardiocentro Ticino, Laboratories for Translational Research, Ente Ospedaliero Cantonale, Bellinzona, Switzerland; 2 Division of Internal Medicine and Hypertension Unit, Department of Medical Sciences, University of Torino, Torino, Italy; 3 Department of Control and Computer Engineering, Politecnico di Torino, Torino, Italy; 4 Neurodegenerative Diseases Group, Laboratories for Translational Research, Ente Ospedaliero Cantonale, Bellinzona, Switzerland; 5 Faculty of Biomedical Sciences, Università della Svizzera italiana (USI), Lugano, Switzerland; 6 Euler Institute, Faculty of Biomedical Sciences, Università della Svizzera italiana (USI), Lugano, Switzerland; 7 Neurology Department, Neurocenter of Southern Switzerland, Ente Ospedaliero Cantonale, Lugano, Switzerland; 8 Cellular and Molecular Cardiology Laboratory, Cardiocentro Ticino Institute, Bellinzona, Switzerland; 9 Department of Medical Sciences, University of Torino, Torino, Italy; 10 Institute of Oncology Research (IOR), Oncology Institute of Southern Switzerland, Bellinzona, Switzerland; 11 Department of Health Sciences and Technology (D-HEST), ETH Zurich, Zurich, Switzerland; 12 Center for Molecular Cardiology, University of Zurich, Schlieren, Switzerland; 13 Department of Research and Education, University Hospital Zurich, Zurich, Switzerland; 14 Division of Physiology and Pathophysiology, Otto Loewi Research Center for Vascular Biology, Immunology and Inflammation, Medical University of Graz, Graz, Austria; 15 Vetsuisse Faculty, University of Zurich, Zurich, Switzerland; 16 Division of Cardiology, Istituto Cardiocentro Ticino, Ente Ospedaliero Cantonale Lugano Switzerland, Zurich, Switzerland

**Keywords:** Biomarkers, Senescence

## Abstract

Extracellular vesicles (EVs) offer valuable diagnostic and prognostic insights for cardiovascular (CV) diseases, but the influence of age-related chronic inflammation (“inflammaging”) and sex differences on EV profiles linked to CV risk remains unclear. This study aimed to use EV profiling to predict age and stratify patients by CV risk. We developed an *EVaging* index by analyzing surface antigen profiles of serum EVs from 625 participants, aged 20 to 94 years, across varying CV risk groups. The *EVaging* index was associated with age in healthy individuals and distinguished CV risk profiles in patients, correlating with CV outcomes and likelihood of fatal CV events according to the European Society of Cardiology (ESC) SCORE, and reflecting age-associated comorbidities. While changes in disease-related EV fingerprint adds complexity in CV patients, EV profiling may help assess biological aging and CV risk, emphasizing EVs’ roles in inflammaging.

## Introduction

Inflammaging is characterized by persistent, low-grade, inflammation driven by cumulative cellular stress, senescent cells, and pro-inflammatory signals that accumulate with age. This chronic, non-resolving inflammation can damage healthy tissues and contribute to the so-called “chronic diseases of aging”, including cancer, diabetes mellitus, chronic kidney disease (CKD), and osteoarthritis^1^. Chronic systemic inflammation is also recognized as a significant contributor to diseases for which aging is a major risk factor, including cardiovascular (CV) conditions at high prevalence such as hypertension, hyperlipidemia, and obesity. These conditions collectively rank among the leading causes of disability and mortality globally. Researchers have developed comprehensive approaches to identify biomarkers that more accurately reflect overall age-related inflammatory activity. This involves measuring a broad spectrum of inflammatory markers, such as canonical inflammatory cytokines (IL-6, IL-1Ra, and IL-1β) and integrating them into indices. Studies have identified significant correlations between these indices and the risk of chronic diseases like CV disease, kidney disease, diabetes, and overall mortality^2^. However, the expression levels of inflammatory markers such as cytokines exhibit inconsistencies between younger and older individuals, particularly when the health status of the older population is rigorously controlled^3,4^. This suggests that the relationship between inflammation and aging is complex and may be influenced more by the presence of chronic diseases and frailty rather than aging alone. Understanding how inflammatory markers co-vary in populations over extended periods and interpreting different inflammatory profiles in clinical or public health contexts remains challenging^2^.

Extracellular vesicles (EVs) are nano-sized, membrane-bound particles released by cells. These vesicles carry proteins, lipids, and nucleic acids that reflect the condition of their cells of origin, making them valuable for understanding the physiological and pathological states associated with aging^5^. The role of EVs as mediators of immune responses has been known since the early ‘90s^6^. The content of these circulating EVs may provide insights into the mechanisms driving inflammaging and its related diseases.

EVs have shown potential diagnostic and prognostic value for a majority of chronic aging diseases, including CV events^7^, neurodegenerative diseases^8^, chronic kidney diseases^9^, diabetes^10^, and more. EV function as immune cell mediators throughout all stages of diseases progression, suggesting that the EV signature is likely indicative of an inflammatory “dynamic state”^11^. We recently described a significant increased levels of EV-specific surface signature in patients with an on-going acute event. Notably, the expression of these specific markers reached their highest levels upon hospital admission, followed by a rapid decline over the subsequent 2 days^12,13^. In healthy subjects, age-related EV content was consistent with senescence-associated biomarker profiles^14–16^.

Given the versatility of EVs in various physiological and pathological conditions, we believe they serve as effective sentinels for understanding the multivariate relationships among age-affected inflammatory markers and clinically relevant conditions. This understanding can aid in interpreting changes in these markers and the underlying biological processes that affect the inflammatory system over extended timescales.

In this study, we developed and validated an EV-based index of aging, termed *EVaging*, by stratifying EV profiles by age decade. Using machine learning techniques, we aim to investigate the ability of our index to predict chronological age and assess its capacity to reflect aging-associated processes and CV risk profiles. Then, the study explores variations in EV antigens contributing to *EVaging* in a cohort of 625 individuals, aged 20 to 94 years, including markers of endothelial, platelet, and immune cell origin, across different age groups and their association with systemic inflammation, coagulation, CV risk, sex, and pharmacological treatments.

## Results

### Characteristics of the enrolled cohort and age stratification

The overall cohort included 625 participants (Supplementary Fig. S1A), which were divided into four groups according to their CV risk profile (see Methods section): (i) Healthy controls (HC; *n* = 132), (ii) Subjects with at least one CV risk factor (*n* = 268), (iii) Patients with established cardiac disease and/or organ damage (OD; *n* = 138), and (iv) Patients after an acute CV event (*n* = 87). Characteristics of the recruited patients are reported in Table 1. Mean age was 55 years, 45.8% were females, with a BMI of 27.5 Kg/sqm and 134/82 mmHg as mean systolic and diastolic blood pressure (BP) levels. Considering CV risk factors, prevalence of hypertension, hyperlipidemia, diabetes, obesity, and CKD was 44.8%, 49.9%, 12.8%, 21.3%, and 10.1%, respectively; 19.0% of patients had history of cardiac disease, and 20.6% displayed known OD (including microalbuminuria, and/or left ventricular hypertrophy). After stratification in the four groups of CV risk (Supplementary Fig. S1B), age increased from 51 to 62 years from group “i” to group “iv”, as well as BP levels (from 123/77 to 146/87 mmHg), and prevalence of the different CV risk indicators (*P* < 0.001 for all comparisons). The biochemical profile of patients from groups “iii” and “iv” was unfavorable as compared to HC and subjects with CV risk factors, with higher levels of glucose, cholesterol, triglycerides, leucocytes, and C-reactive protein (CRP), and lower eGFR (Table 1). After stratification for decades of age (from less than 40 years, to equal or more than 70 years; Supplementary Table S1), we observed that older patients displayed higher BP levels (from 126/79 to 144/83 mmHg), a lower BMI (from 29.8 to 26.1 Kg/sqm), and an increasing prevalence of hypertension, hyperlipidemia, CKD, OD, and cardiac disease. Moreover, CRP displayed a linear and progressive increase at the increase of age (*P* < 0.001).

**Table 1:** Characteristics of the enrolled cohort

Variable	Overall cohort (*n* = 625)	Healthy controls (*n* = 132)	CV risk factor (*n* = 268)	Organ damage (*n* = 138)	Acute CV event (*n* = 87)	*P-*value
Age (years)	55 ± 15.8	51 ± 15.4	53 ± 15.8	58 ± 15.1	62 ± 14.1	**<0.001**
Sex (ref. female)	286 (45.8)	69 (52.3)	154 (57.6)	45 (32.6)	18 (20.7)	**<0.001**
SBP (mmHg)	134 ± 20.6	123 ± 17.1	132 ± 19.0	138 ± 20.0	146 ± 22.1	**<0.001**
DBP (mmHg)	82 ± 11.8	77 ± 10.4	82 ± 11.2	84 ± 11.7	87 ± 13.0	**<0.001**
BMI (Kg/sqm)	27.5 ± 5.50	26.0 ± 2.91	28.8 ± 6.62	27.1 ± 5.20	26 ± 4.1	**<0.001**
Hypertension (ref. yes)	280 (44.8)	0 (0.0)	132 (49.3)	88 (63.8)	60 (69.0)	**<0.001**
Hyperlipidemia (ref. yes)	312 (49.9)	0 (0.0)	175 (65.3)	81 (58.7)	56 (64.4)	**<0.001**
Type 2 Diabetes (ref. yes)	80 (12.8)	0 (0.0)	26 (9.7)	42 (30.4)	12 (13.8)	**<0.001**
Obesity (ref. yes)	133 (21.3)	0 (0.0)	90 (33.6)	33 (23.9)	10 (11.5)	**<0.001**
CKD (ref. yes)	63 (10.1)	0 (0.0)	34 (12.7)	23 (16.7)	6 (6.9)	**<0.001**
Cardiac Disease (ref. yes)	119 (19.0)	0 (0.0)	0 (0.0)	99 (71.7)	20 (23.0)	**<0.001**
Organ damage (ref. yes)	129 (20.6)	0 (0.0)	0 (0.0)	73 (52.9)	56 (64.4)	**<0.001**
Glucose (mmol/L)	5.7 ± 1.86	5.3 ± 1.81	5.2 ± 1.32	6.3 ± 2.15	6.4 ± 2.30	**<0.001**
Tot Cholesterol (mmol/L)	5.1 ± 1.21	4.5 ± 0.91	5.2 ± 1.14	5.1 ± 1.21	5.4 ± 1.48	**<0.001**
HDL (mmol/L)	1.4 ± 0.63	1.4 ± 0.37	1.4 ± 0.80	1.3 ± 0.44	1.4 ± 0.59	0.756
Triglycerides (mmol/L)	1.7 ± 1.20	1.2 ± 0.68	1.9 ± 1.27	1.5 ± 1.04	2.0 ± 1.54	**<0.001**
LDL (mmol/L)	3.0 ± 0.97	2.6 ± 0.84	3.2 ± 0.90	3.1 ± 1.01	3.1 ± 1.08	**<0.001**
Creatinine (mg/dL)	0.98 ± 0.468	0.88 ± 0.176	0.96 ± 0.483	1.10 ± 0.677	0.97 ± 0.197	**0.001**
eGFR (mL/min)	86 ± 25.1	90 ± 18.1	86 ± 26.8	81 ± 27.3	86 ± 24.2	**0.048**
WBC (n/L*1000)	8.0 ± 2.87	6.8 ± 2.15	7.7 ± 2.35	7.5 ± 2.58	9.5 ± 3.49	**<0.001**
Neutrophils (n/L*1000)	5.4 ± 2.64	4.3 ± 1.93	5.1 ± 2.27	5.0 ± 2.28	6.9 ± 3.13	**<0.001**
Lymphocytes (n/L*1000)	1.9 ± 0.81	2.0 ± 0.78	2.0 ± 0.68	1.9 ± 0.70	1.9 ± 1.05	0.924
Monocytes (n/L*1000)	0.43 ± 0.152	0.40 ± 0.133	0.44 ± 0.143	0.42 ± 0.157	0.44 ± 0.168	0.494
Basophils (n/L*1000)	0.05 ± 0.031	0.04 ± 0.025	0.05 ± 0.035	0.05 ± 0.031	0.05 ± 0.031	0.825
Eosinophils (n/L*1000)	0.15 ± 0.107	0.12 ± 0.078	0.17 ± 0.120	0.14 ± 0.102	0.14 ± 0.110	0.094
C-reactive Protein (mg/L)	4.8 ± 6.19	2.1 ± 1.9	3.5 ± 3.13	5.6 ± 6.53	10.8 ± 10.57	**<0.001**
Hb (g/L)	144 ± 13.4	141 ± 12.7	146 ± 15.6	142 ± 13.3	145 ± 10.6	0.184
Platelets (n/L*1000)	251 ± 60.6	238 ± 51.9	258 ± 55.4	247 ± 69.5	256 ± 62.5	0.334

The table reports characteristics of patients included in the study. A *P*-value <0.05 was considered significant and showed in bold.

*SBP* systolic blood pressure, *DBP* diastolic blood pressure, *CKD* chronic kidney disease (eGFR lower than 60 mL/min), *HDL/LDL* high/low density lipoproteins, *eGFR* estimated glomerular filtration rate, *WBC* white blood cells, *Hb* haemoglobin.

**Fig. 1:**
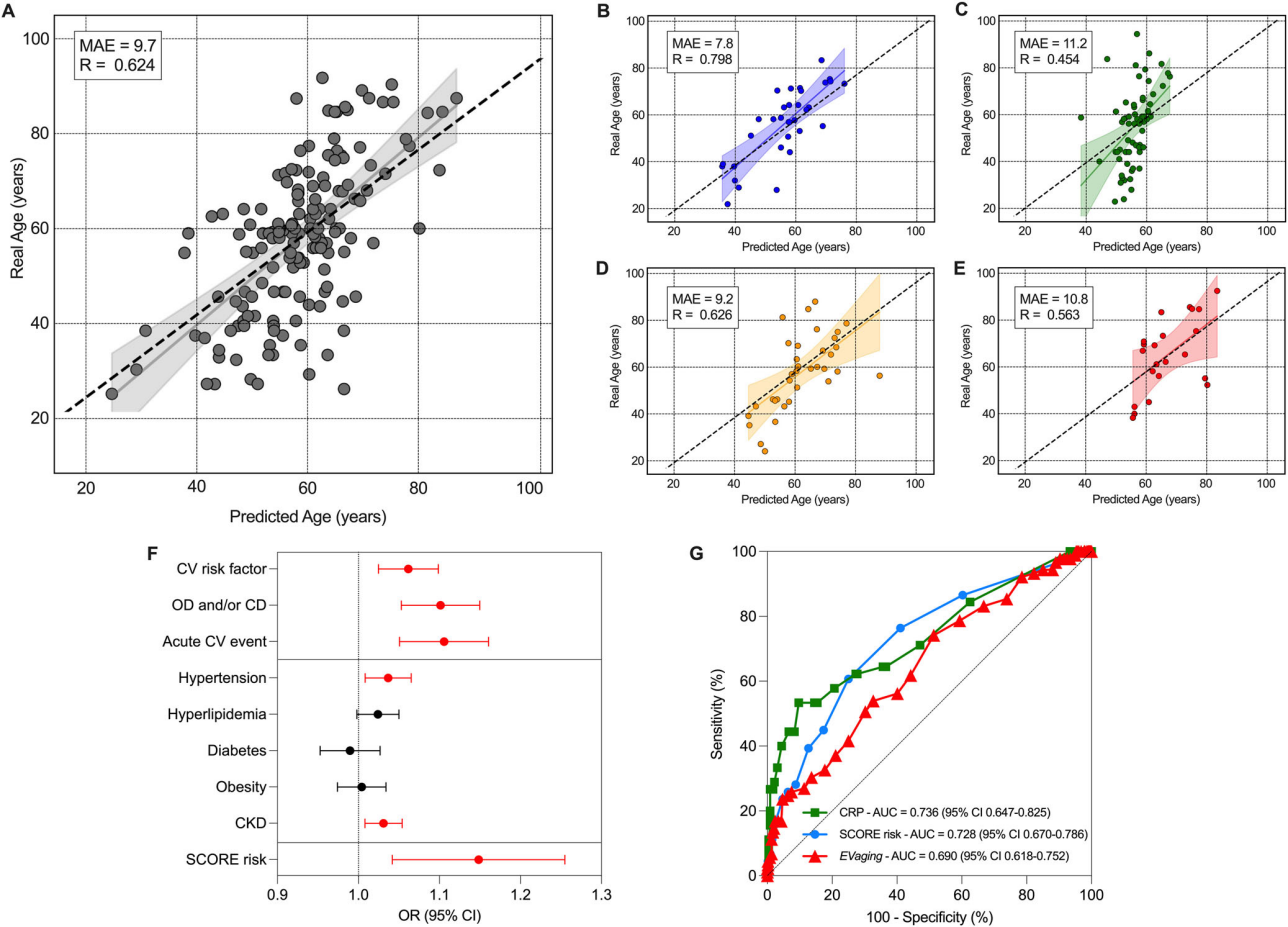
EV profiling predicts the age: the *EVaging* index. Data from EV profiling were used to train and validate a machine learning model (support vector regressor with RBF kernel) able to predict the age starting from levels of the 37 evaluated EV surface antigens in the overall cohort (**A**) and among healthy controls (**B**), patients with a CV risk factor (**C**), with organ damage and/or cardiac disease (**D**), and those after an acute CV event (**E**). For model generation, 75% of the dataset was used for training and 25% for testing. Prediction curves and 95% confidence intervals, mean absolute error (MAE), and Pearson’s R are shown for each model at validation (see Supplementary Fig. S2 for the visualization of *EVaging* performance at training of the model). **F** Forest plot reporting age-adjusted logistic and linear regression models to associate *EVaging* index with single CV risk indicators and ESC (European Society of Cardiology) SCORE risk. **G** ROC curves comparing the performance of C-reactive protein (CRP), ESC SCORE risk, and *EVaging* index in the detection of an acute CV event.

**Fig. 2:**
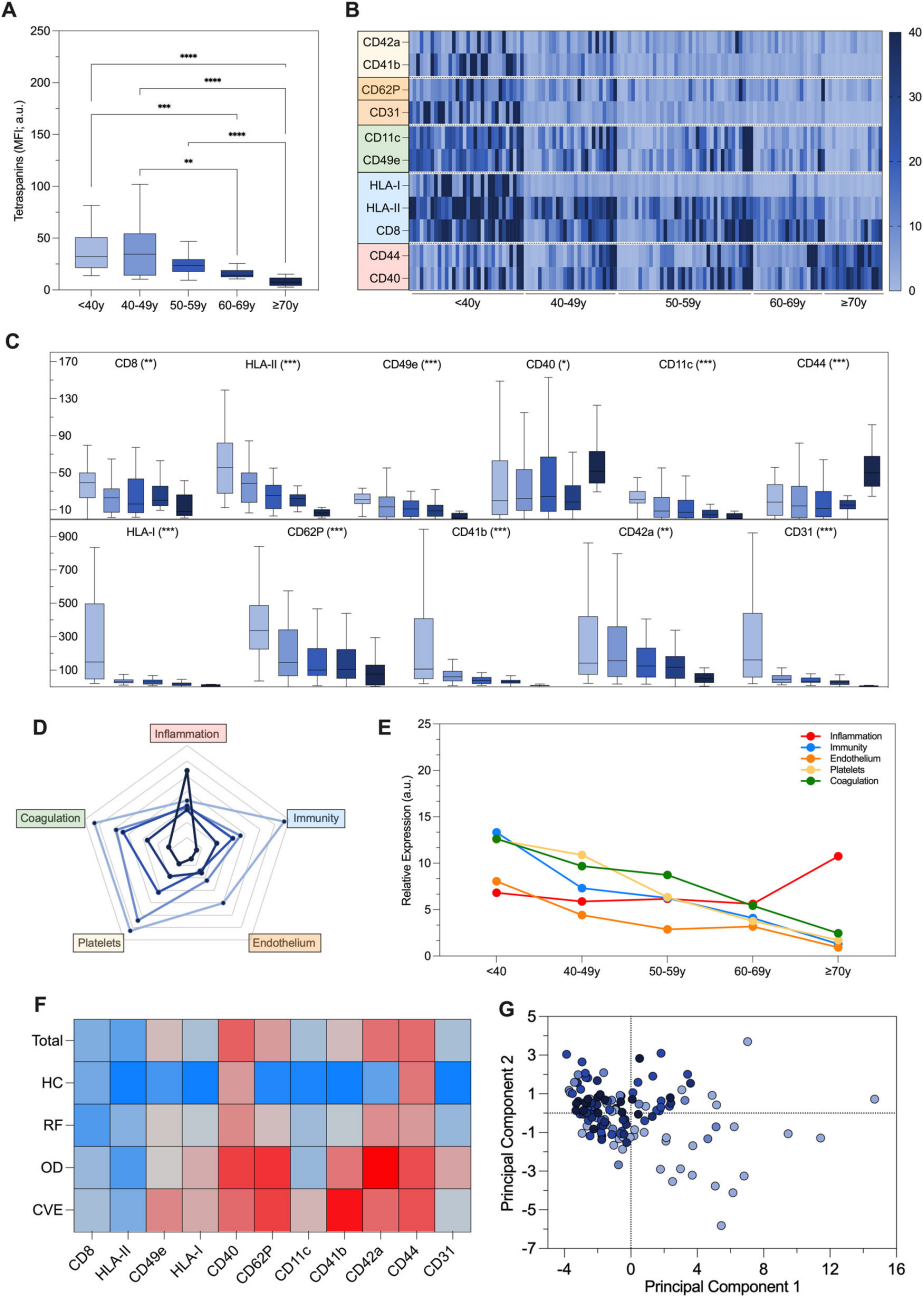
EV profiling in healthy controls. EV surface antigens were evaluated by FC in 132 healthy controls (HC), after stratification for age. **A** Tetraspanin levels (MFI; a.u.). **B** Heat map reporting EV markers differentially expressed in HC. **C** EV antigens differentially expressed in HC after stratification for age. **D** Radar charts of putative biological processes attributed to EV antigens differentially expressed in HC (platelets [CD41b, CD42a, CD62P]; endothelium [CD31, CD62P]; coagulation [CD11c, CD49e]; immunity [CD8, HLA-I, HLA-II]; inflammation [CD40, CD44]). **E** Evaluation of biological processes associated with differentially expressed EV markers during aging. **F** Matrix showing correlations between age and EV antigens (scale ranging between blue and red, for inverse and direct correlations. **G** Principal component analysis showing an age shift of patients visualized according to their EV profile; each point represents a patient and decades of age are reported in shade of blue (light = younger; dark = older). Risk factors (RF); Cardiovascular events (CVE). *P < 0.01; **P < 0.05; ***P < 0.001; ****P < 0.0001.

**Fig. 3:**
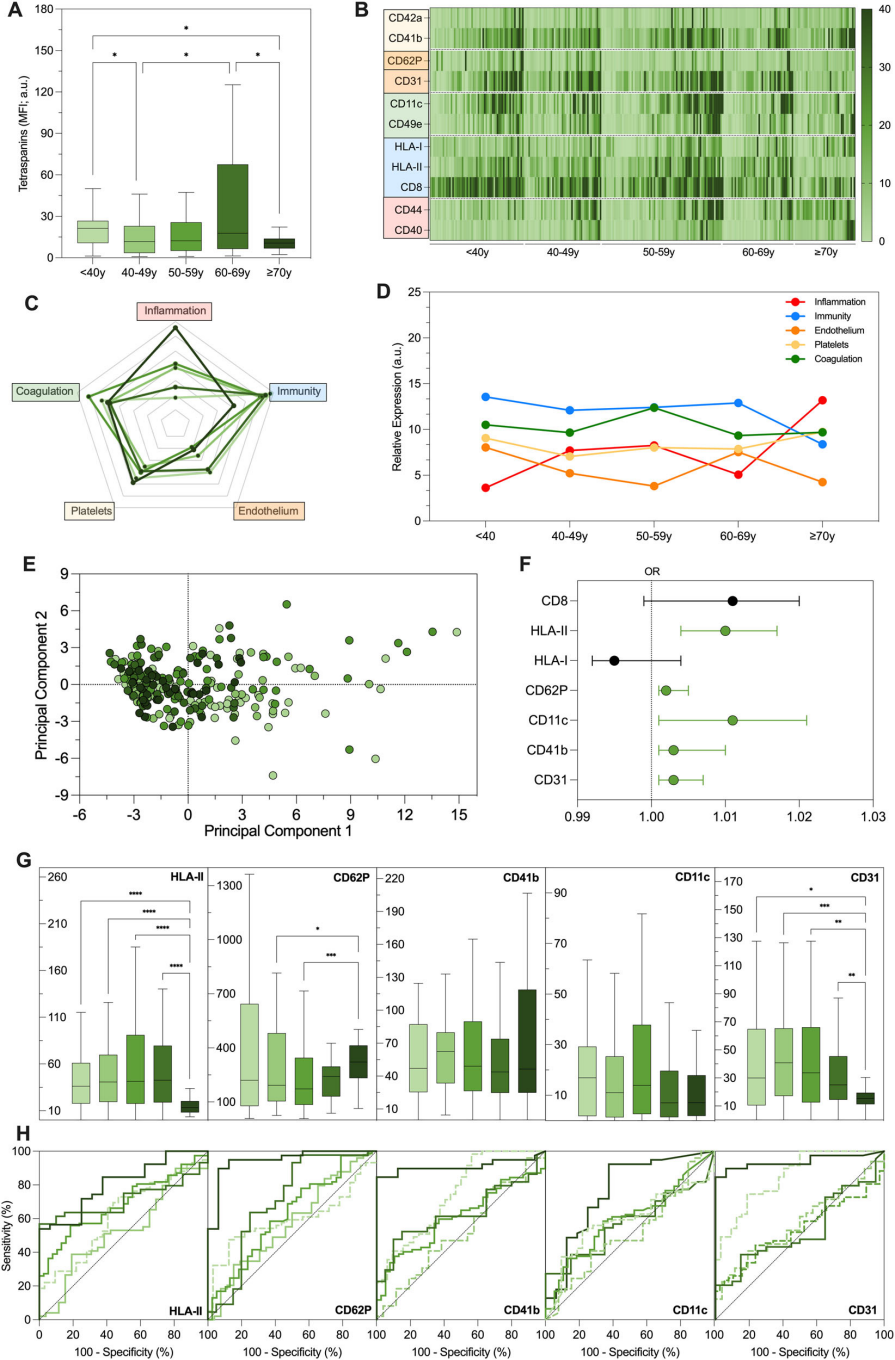
EV profiling in subjects with cardiovascular risk factors. EV surface antigens were evaluated by FC in 268 subjects with at least one CV risk factor (hypertension, hyperlipidemia, diabetes, obesity, or CKD), after stratification for age. **A** Tetraspanin levels (MFI; a.u.). **B** Heat map reporting, in this cohort, EV markers differentially expressed in HC. **C** Radar charts of putative biological processes attributed to EV antigens differentially expressed in HC (platelets [CD41b, CD42a, CD62P]; endothelium [CD31, CD62P]; coagulation [CD11c, CD49e]; immunity [CD8, HLA-I, HLA-II]; inflammation [CD40, CD44]). **D** Evaluation of biological processes associated with differentially expressed EV markers during aging. **E** Principal component analysis showing an age shift of patients visualized according to their EV profile; each point represents a patient and decades of age are reported in shade of green (light = younger; dark = older). **F** Forest plot reporting EV antigens associated with the presence of a CV risk factor, independently from age (in green). **G** EV antigens associated with a CV risk factor independently from age, after stratification for age (nMFI; %). **H** Discrimination of subjects with at least a CV risk factor from HC; ROC curves were drawn for EV antigens associated with a CV risk factor, independently from age, after stratification for decades of age (green; light = younger, dark = older). *P < 0.01; **P < 0.05; ***P < 0.001; ****P < 0.0001.

**Fig. 4:**
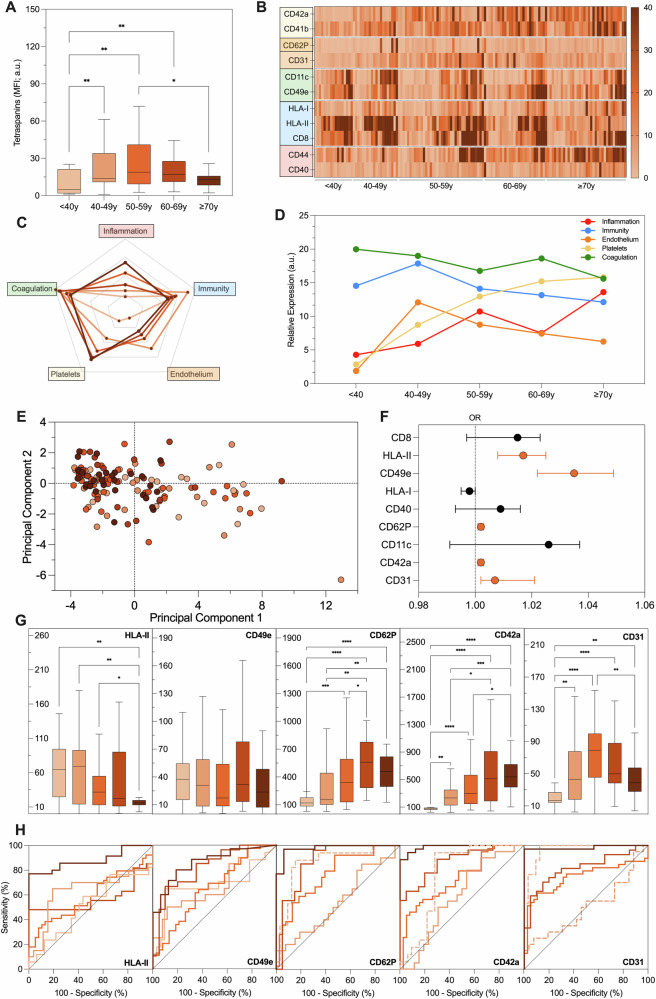
EV profiling in patients with organ damage and/or established cardiac disease. EV surface antigens were evaluated by FC in 138 patients with established cardiac disease (coronary artery disease, and or chronic heart failure) and/or OD (microalbuminuria, or left ventricular hypertrophy), after stratification for age. **A** Tetraspanin levels (MFI; a.u.). **B** Heat map reporting, in this cohort, EV markers differentially expressed in HC. **C** Radar charts of putative biological processes attributed to EV antigens differentially expressed in HC. **D** Evaluation of biological processes associated with differentially expressed EV markers during aging. **E** Principal component analysis showing an age shift of patients visualized according to their EV profile; each point represents a patient and decades of age are reported in shade of orange (light = younger; dark = older). **F** Forest plot reporting EV antigens associated with the presence of cardiac disease and/or OD, independently from age (in orange). **G** EV antigens associated with cardiac disease/OD independently from age, after stratification for age (nMFI; %). **H** Discrimination of subjects with at established cardiac disease and/or OD from HC; ROC curves were drawn for EV antigens associated with a cardiac disease/OD, independently from age, after stratification for decades of age. *P < 0.01; **P < 0.05; ***P < 0.001; ****P < 0.0001.

**Fig. 5:**
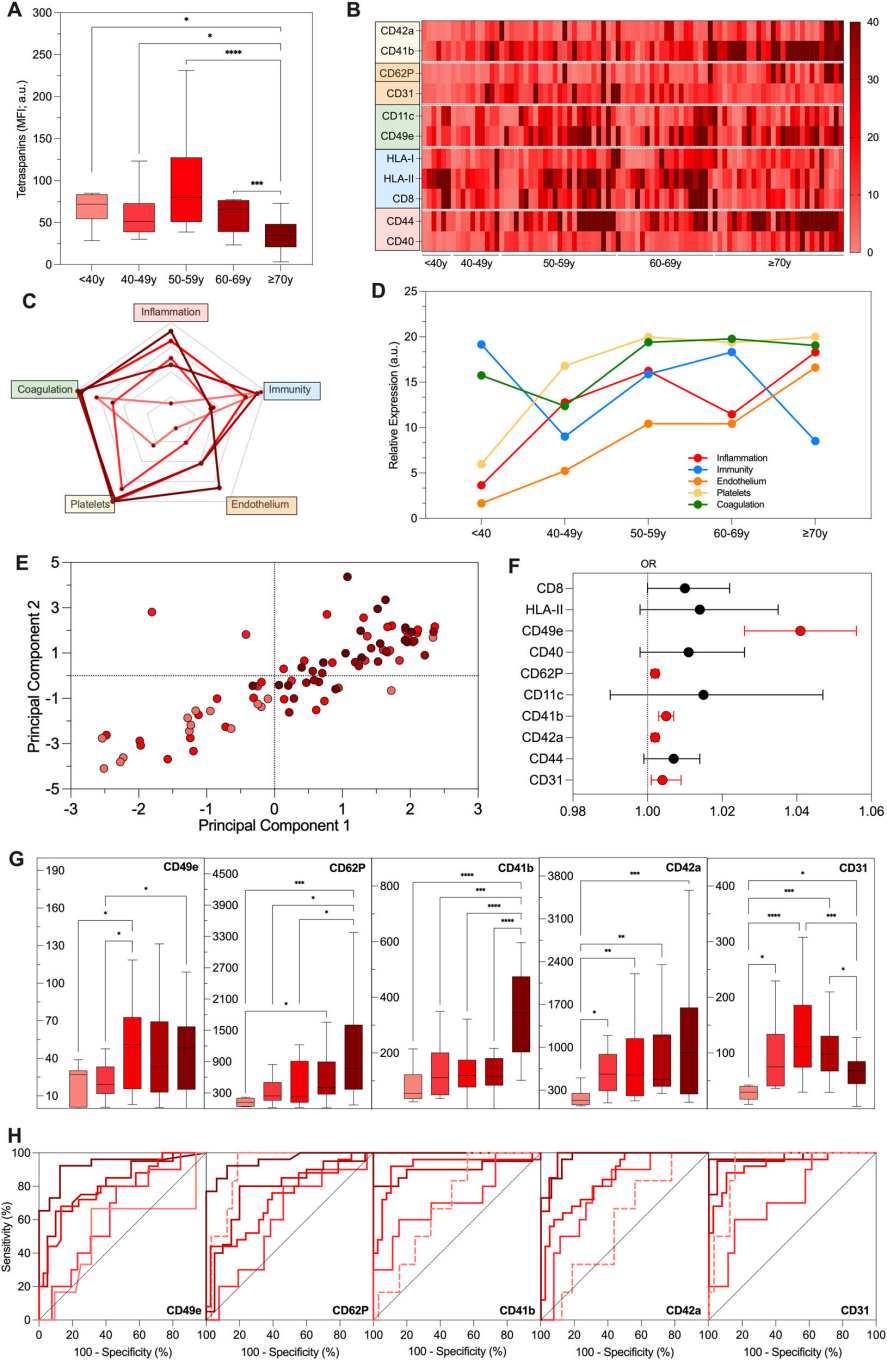
EV profiling in patients after a cardiovascular event. EV surface antigens were evaluated by FC in 87 patients after an acute CV event, after stratification for age. **A** Tetraspanin levels (MFI; a.u.). **B** Heat map reporting, in this cohort, EV markers differentially expressed in HC. **C** Radar charts of putative biological processes attributed to EV antigens differentially expressed in HC. **D** Evaluation of biological processes associated with differentially expressed EV markers during aging. **E** Principal component analysis showing an age shift of patients visualized according to their EV profile; each point represents a patient and decades of age are reported in shade of red (light = younger; dark = older). **F** Forest plot reporting EV antigens associated with an acute CV event, independently from age (in red). **G** EV antigens associated with an acute CV event independently from age, after stratification for age (nMFI; %). **H** Discrimination of subjects after an acute CV event from HC; ROC curves were drawn for EV antigens associated with acute CV events, independently from age, after stratification for decades of age. *P < 0.01; **P < 0.05; ***P < 0.001; ****P < 0.0001.

EV analysis by flow cytometry (FC) was performed on serum aliquots from each subject in the study. Using machine learning algorithms that employ supervised learning, a predictive model was developed based on the profiling of EV surface antigens. This model aims to predict chronological age while also reflecting the increasing incidence of aging-related CV comorbidities (Fig. 1). Next, data were analyzed after stratification for groups of CV risk and decades of age (Figs. 2–5 and Supplementary Tables S2–S8; Supplementary Figs. S2–S4). Tetraspanins (CD9, CD63, and CD81, commonly referred to as pan-EV markers because they are expressed by all types of EVs) and EV antigens differentially expressed in HC (Supplementary Table S2) were analyzed in all the other groups, looking at how they changed in presence of CV risk factors, OD, and/or cardiac disease, or after an acute CV event. Finally, we performed a sub-analysis dividing the cohort between females and males, to evaluate the effect of sex on EV profile and the association of the latter with CV risk indicators (Supplementary Fig. S5 and Supplementary Tables S9–S21).

### An aging index based on extracellular vesicles profiling: *EVaging*

Normalized median fluorescence intensity (nMFI) levels for each of the 37 EV antigens were combined in an index able to predict patients’ age based exclusively on the EVs biomolecular fingerprint, namely the *EVaging* index. From the overall cohort, 468 subjects were used to train (Supplementary Fig. S2), and the remaining 157 to validate the machine learning model (Fig. 1). Patients were randomly assigned to the training or to the validation dataset; we employed a supervised algorithm of support vector regressor to build the model (see methods).

Considering the overall cohort, *EVaging* was able to predict the real age of patients with a mean absolute error (MAE) of 9.7 years at validation; the regression analysis comparing real and predicted age displayed a good correlation with an R of 0.624 (Fig. 1A). At subgroup analysis, our index displayed higher performance in HC (MAE = 7.8 years, with an R = 0.798), while the prediction accuracy was lower in patients with at least a CV risk factor, OD and/or established cardiac disease, or in case of an acute CV event (MAE ranging between 9.2 and 11.2 years; R = 0.454-0.626; Fig. 1B–E). This is expected considering that these conditions are associated with endothelial dysfunction, platelets activation, and vascular inflammation, which influence circulating EV antigens. In age-adjusted regression models, *EVaging* index was associated with some of the explored CV risk indicators, including hypertension, CKD, presence of at least one CV risk factor, OD and/or cardiac disease, and acute CV events (OR from 1.037 up to 1.105; Supplementary Table S22 and Fig. 1F). Notably, *EVaging* was also significantly associated with the 10-years likelihood of fatal CV events estimated by the European Society of Cardiology (ESC) SCORE risk (OR 1.1248 - *P* < 0.001), and able to discriminate patients experiencing an acute CV event with an AUC of 0.690, similarly to CRP and SCORE risk (AUC of 0.736 – *P* = 0.313, and 0.728 – *P* = 0.406, respectively; Fig. 1G).

### Modulation of EV markers defining *EVaging* index

Considering the 37 antigens defining the *EVaging* index, we found an inverse significant correlation of lymphocytes and age in subjects with OD/established cardiac disease and those with an acute CV event (Rho = −0.278/−0.265; *P* < 0.005; Supplementary Fig. S3A and Supplementary Table S18), whereas CRP was directly correlated to age only in HC, reflecting chronic systemic inflammation (Rho = 0.724; *P* < 0.001; Supplementary Fig. S3B and Supplementary Table S18). We also found that EV surface antigens derived by T- (CD2, CD3, CD4, CD8, CD14, CD69, CD86) /B- lymphocytes (CD19, CD20, CD24), and those reflecting inflammation (CD40, CD44) decreased at the increase of CRP, even if not significantly, in all groups except for patients with an acute CV event (Supplementary Fig. S3C and Supplementary Table S19). Moreover, EV markers of T- and B- lymphocytes were inversely correlated to age (minimum Rho of −0.333), whereas CD40 and CD44 increased at the increase of age (Rho ranging between 0.141 and 0.393), consistently with the concept of inflammaging (Supplementary Fig. S3D and Supplementary Table S20) characterized by a substantial reduction in adaptive immune responses alongside persistent, low-grade inflammation. Levels of EV surface antigens may be associated to conventional biochemical parameters, reflecting not only inflammation but also CV outcomes, renal function, and gluco-lipidic profile (Supplementary Table S21 and Supplementary Fig. S4). Levels of CD62P were correlated with the number of platelets, CD44 and CD31 with eGFR, and HLA-I/II, CD49e, CD40, CD62P, CD42a, CD44, and CD31 with glucose, total cholesterol, and HDL.

### EV profiling in healthy controls

The group of HC included 132 subjects with a mean age of 51 years, 52.3% were females, with BP levels of 123/77 mmHg, a BMI of 26.0 Kg/sqm, preserved renal function, and a normal gluco-metabolic profile, blood cell count and CRP (Table 1). EVs profiling is reported in Fig. 2 after stratification for decades of age (see also Supplementary Table S2). Mean MFI for tetraspanins progressively decreases with aging (4.2-fold decrease in patients with an age equal to or higher than 70 years compared to those with less the 40 years; Fig. 2A). Normalized levels of eleven EV antigens showed differential expression with aging. Specifically, CD41b, CD42a, CD62P, CD31, CD11c, CD49e, CD8, HLA-I, and HLA-II progressively decreased across different age decades, whereas CD40 and CD44 levels increased with age (Fig. 2B, C).

Clustering these EV markers by biological functions, at the increase of age, we observed an increase of antigens associated with inflammation, and a decrease of all the other markers from endothelium, platelets, immune system cells, or involved in coagulation (Fig. 2D, E). Consistently, levels of the 11 differentially expressed EV markers were inversely correlated with age, except for CD40 and CD44, which displayed a direct correlation with aging (Fig. 2F and Supplementary Table S6; from blue to red color code for Rho ranging between -1 and 1, respectively). Finally, a principal component analysis (PCA) combining levels of expression of single EV antigens in a specific fingerprint was used to visualize patient distribution: the plot displayed an apparent age-related shift (Fig. 2G), discriminating between the youngest and the eldest subjects, consistently with what observed with *EVaging* index.

### EV profiling in patients with cardiovascular risk factors

Subjects with at least one risk factor, including hypertension, hyperlipidemia, diabetes, obesity, or CKD (*n* = 268) were classified as being at CV risk. The median age of these subjects was 53 years, 57.6% being female. The mean blood pressure was 132/82 mmHg, and the mean BMI was 28.8 kg/m². This group exhibited elevated levels of total cholesterol and triglycerides, while glucose levels, renal function, blood cell count, and CRP levels remained within normal ranges (Table 1). As for EV markers, we observed a reduction in tetraspanin levels, although the decrease was less pronounced (2.0-fold decrease) and exhibited less linearity compared to the HC (Fig. 3A). Age-related differentially expressed antigens in HC demonstrated substantial stability across various decades of aging within this group. However, subjects over 70 exhibited a significant increase in inflammatory markers and a corresponding decrease in immunity-related markers compared to those aged 60–69 (Fig. 3B–D). The PCA displayed, again, a shift across the different age decades (Fig. 3E).

Age-adjusted multivariate analysis confirmed the association of HLA-II, CD62P, CD11c, CD41b and CD31 (OR ranging between 1.001 and 1.011; Fig. 3F and Supplementary Table S7); HLA-II and CD31 showed a significant decrease, whereas CD62P increased when considering in subject aging >70 years, in particular in eldest patients; no change was observed for CD41b and CD11c, which were then associated with presence of at least one CV risk factors, independently from age (Fig. 3G). Variations of vesicular markers with aging resulted in a different performance in the discrimination of patients with at least one CV risk factor from HC in the different decades of age: low levels of HLA-I and CD31 displayed a high accuracy in the discrimination of CV risk factors in younger patients, whereas high levels of CD62P and CD31 correctly classified most of older patients, with AUC up to 0.946 (Fig. 2H and Supplementary Table S8).

### EV profiling in patients with organ damage and/or cardiac disease

Patients with OD (microalbuminuria, or left ventricular hypertrophy) and/or established cardiac disease (coronary artery disease, or chronic heart failure) (*n* = 138) were older, with a mean age of 58 years-old, 32.6% were females, with higher BP levels (138/84 mmHg), and an overall less favorable CV risk profile, with a higher prevalence of hypertension, diabetes, and CKD; a slight increase was observed also in levels of CRP (Table 1). EV antigens differentially expressed in HC were evaluated within this group (Fig. 4 and Supplementary Table S4). Tetraspanins, CD31, CD41b, CD42a, and CD62P showed an inverse U-shape, with the highest levels occurring in the 50–59 and 60–69 age decades. In contrast, HLA-II levels decreased with age, while CD40/CD44 levels increased (Fig. 4A, B). When clustering by biological function, we observed a decrease of immunity- and endothelial-related EV markers, along with an increase of inflammation- and platelet activation-related antigens (Fig. 4C, D). At PCA, we observed only a modest age shift of the EV-specific fingerprints (Fig. 4E), consistently with a lower age prediction accuracy of our *EVaging* index. The age-adjusted multivariate regression confirmed the association of HLA-II, CD49e, CD62P, CD42b, and CD31 (OR between 1.002 and 1.035; Fig. 4F and Supplementary Table S7): as expected, HLA-II progressively decreased, and CD42a increased with aging, while we observed the highest levels for CD62P and CD31 between 50 and 69 years (Fig. 4G), resulting in heterogenous diagnostic performance at ROC curves analysis (Fig. 4H). In the detection of OD and cardiac disease, HLA-II reached a quite reliable performance only in patients with an age equal or higher than 70 years (AUC = 0.880), while CD62P, CD42a, and CD31 were highly accurate from 60 years of age (AUC up to 0.979–0.988); again, low levels of CD31 suggested the presence of CV risk indicators (in this case OD and/or cardiac disease) in younger patients (<40 years; AUC = 0.967; Supplementary Table S8). We acknowledge that patients with OD may have a different CV risk profile as compared to those with established cardiac disease; we then performed a sub-analysis dividing patients accordingly (Supplementary Table S23). As expected, levels of several EV antigens (CD8, CD8, CD1c, CD25, CD62P, CD42a, CD86, CD44) increased in subjects displaying both OD and established cardiac disease (coronary artery disease, or chronic heart failure), as compared with those with only OD or cardiac disease. A slight increase was observed in levels of CD62P and CD42a in patients with cardiac disease compared to those with OD.

### EV profiling in patients with an acute cardiovascular event

We evaluated a cohort of patients after an acute CV event (*n* = 87; myocardial infarction, or cerebrovascular event; Fig. 5). They were the eldest subjects included in our study (mean age 62 years old), with the lowest prevalence of females (20.7%) and the highest mean levels of BP (146/87 mmHg). These patients displayed the worst CV risk profile, with the highest prevalence of most of the evaluated CV risk indicators and an increase of inflammatory markers (white blood cells and CRP) as compared to the other groups (Table 1).

EV profiling showed linear decrease of tetraspanins starting from decades 50-59 with aging (2.1-fold decrease versus >70 years; Fig. 5A); among EV antigens which were differentially expressed in HC, HLA-II decreased, CD40, CD41b, CD42a, CD44, CD49e, CD62P increased, while CD31 displayed an inverse U-shape peaking at 50–59 years (Fig. 5B and Supplementary Table S5). Regarding EV markers-associated biological functions, they all increased with aging, except for immunity which increased only temporarily at 60–69 years of age and then decreased sharply in eldest patients (Fig. 5C, D). The age shift of EV fingerprint was evident in this cohort at very high CV risk, with a clear discrimination of youngest patients (Fig. 5E), and again five EV markers were significantly associated with the endpoint (acute CV event) at age-adjusted multivariate analysis: CD49e, CD62P, CD41b, CD42a, and CD31 (OR up to 1.041; Fig. 5F and Supplementary Table S7). The levels of all these EV antigens displayed significant changes across the age decades, resulting also in this case in very heterogenous diagnostic performance at ROC curves analysis (Fig. 5G, H). All the evaluated EV markers displayed high performance of discrimination in patients aged 70 years or older (AUC between 0.925 and 1.000), while CD31, CD41b, and CD42a were very accurate from the age of 50 years (AUC higher than 0.845); interestingly, low levels of CD31, and CD62P were diagnostic for a CV event in patients under 40 years of age (AUC of 0.922 and 0.906, respectively; Supplementary Table S8).

### EV profiling after stratification for sex, age, and pharmacological treatment

Considering sex as an important demographic factor which may affect either EV profiling and CV risk profile, we performed a sub-analysis of our cohort, comparing females and males (Supplementary Table S9). Males were older (53 *versus* 57 years), with higher BP levels and a higher prevalence of hypertension, hyperlipidemia, cardiac disease, and OD; in addition, CRP levels were increased in males as compared to females subjects (*P* < 0.05 for all comparisons). After stratification for sex (Supplementary Tables S10–S15), EV profiling showed a slightly higher expression of CD20, CD40, CD44, CD86, and CD133/1 in females as compared to male HC (Supplementary Fig. S5A); the same trend was observed in subjects with at least one CV risk factor (Supplementary Fig. S5B), while no significant differences were observed in patients with OD and/or cardiac disease, and in those after an acute CV event (Supplementary Fig. S5C, D). We then performed a multivariate analysis adjusted for both age and sex (Supplementary Fig. S5E, G, I and Supplementary Table S16), which identified CD11c, CD31, and HLA-II, as independent predictors of a CV risk factor, CD20, CD42a, CD49e, CD62P, and HLA-II as predictors of OD/cardiac disease, and CD31, CD42a, CD44, CD49e, and CD62P, as predictors of a CV event. We finally evaluated the diagnostic performance of these EV markers, stratifying ROC curve analyses for decades of age and sex (Supplementary Fig. S5F, H, J) and confirming a significant effect of these parameters on the discrimination of patients with a CV risk indicator (AUC ranging between 0.508 and 1.000, with a generally higher accuracy in older female subjects; Supplementary Table S17).

Most recruited patients were on a daily chronic medication regimen with one or more medications (313 of the 493 patients belonging to groups ii to iv). Medications could alter the expressions of several EV markers; to address this issue, we performed a sub-analysis in groups of patients with at least one CV risk factor, OD and/or established cardiac disease, and after an acute CV event, after stratification for treatment with antihypertensive medications, lipid-lowering drugs, hypoglycemic agents (including insulin), antiplatelets, and anticoagulants (Supplementary Table S24). Minor differences were observed in levels of CD8, CD49e, and CD11c after treatment with hypoglycemic agents, and in levels of CD62P, CD42a, CD44, and CD31, after treatment with anticoagulants, in patients with organ damage and/or cardiac disease. Overall, the evaluated pharmacological treatment did not display a significant influence on the evaluated EV antigens.

## Discussion

Our study showed that the *EVaging* index is a promising predictor of chronological age, with a mean absolute error of 9.7 years overall, and 7.8 in HC at validation. At same time it reflects conditions and processes related to aging, as it is associated with various CV risk indicators and the 10-years likelihood of fatal CV events, as estimated by the ESC SCORE risk^7^. In healthy individuals, EV-associated surface markers contributing to *EVaging*, particularly those related to immunity and inflammation, showed an age-related decrease in markers of T- and B-cell effectiveness and functionality (CD8, HLA-I, HLA-II). Conversely, markers associated with the low-grade, chronic activation of innate immune cells, such as macrophages and antigen-presenting cells (CD40 and CD44), increased with age. Additionally, markers like CD31, CD42a, CD44, CD49e, and CD62P were associated with a worse CV profile, showing higher diagnostic accuracy in older *versus* younger subjects, and in females compared to male patients. Pharmacological treatments (hypoglycemic agents and antiplatelets/anticoagulants) showed minor effects on certain EV markers, but did not significantly impact overall EV expression (Supplementary Fig. S1A).

The clinical utility of biomarkers lie in their ability to enhance understanding of disease processes and contribute to better health outcomes^17^. Therefore, the validation of an aging biomarker involves showing that it has predictive power for both chronological age and aging-associated outcomes. Our *EVaging* index performance, as confirmed by strong correlations to clinical data, suggests that the variation in EV surface antigens mirrors the aging process. In patients, systemic inflammation and tissue damage, associated with CV comorbidities, likely alter the composition and expression levels of EV surface antigens, thereby disrupting the model’s predictive capability for chronological aging and reflecting the underlying disease processes. We recently demonstrated that the expression levels of EV-CD62P, EV-CD42a, and EV-CD31 exhibited an 8.2–12.2% increase for each 1% increase in the 10-year risk of a fatal CV event, independent of other CV risk indicators and displayed a 2.3- to 2.9-fold increase in patients with an acute ischemic event as compared to controls^7^. In the present study, we further refined this association by examining age-related variations in the performance of these markers. We found that low levels of EV-CD31 and EV-CD41b identified CV risk factors in younger patients, whereas high levels of EV-CD62P, EV-CD41b, and EV-CD31 were more effective in older patients, achieving an AUC of up to 0.946. Moreover, as expected, levels of different EV markers were also correlated to biochemical parameters related to CV outcome, renal function, and gluco-lipidic profile.

As it is well known, sex influences CV risk and women as up until menopausal age exhibit a lower CV risk due to their hormonal profile^18^ and the EV-specific signature. Few studies have explored sex differences in EVs and their associated cargo. Concentrations often show no sex differences, while EV transcriptome is distinctly different from, and more sex-variant than, the transcriptome of cell-of-origin^19,20^. As for studies focusing on surface antigens, most of them have focused narrowly on selected EV cargo populations^21^. Studies using flow cytometry to analyze EVs in healthy controls found no significant sex differences in the surface levels of markers like CD41, CD105, CD235 and CD142^22^. Similarly, the number of CD62E and CD31 positive EVs (biomarkers of activated endothelial cells) showed no sex differences in blood^21^. In our study, by including a more comprehensive profiling of EVs, we confirmed the absence of sex differences in the previously cited markers, and identified five out of 37 EV antigens that were differentially expressed, with a tendency to be more highly expressed in females compared to healthy males. Notably, when profiling EVs in subjects with CV risk factors and stratifying by sex, CD31 and CD62P (different from CD62E but also a marker of endothelial cell activation) emerged as sex-specific markers, showing the highest level of significance, with markedly increased expression in females. Collectively, these findings highlight that EV markers are valuable for assessing CV profile with great potential for tailored age- and sex-adjusted CV risk assessment and management.

The *EVaging* index reflects fundamental inflammaging biology. A traditional understanding of inflammaging suggests that low-grade inflammation increases during aging^23^. This is characterized by the activation of immune components that are distinct from those engaged during an acute response with a substantial reduction in the adaptive immune responses^24^. This is primarily due to thymic atrophy, which leads to a decrease in the frequency of naïve cells and a relative increase in the prevalence of memory cells in the peripheral blood. Indeed, as we age, the susceptibility to new viral infections and tumors increases, and vaccine efficacy is reduced, while the specific response to previously encountered pathogens can result in persistent infections^24,25^. Such chronic, low-grade inflammation is further promoted by the accumulation of senescent cells in aging organs and tissues, which secrete proinflammatory factors known as the senescence-associated secretory phenotype (SASP). For example, CCL2, secreted by senescent skin cells, acts as a chemokine, attracting proinflammatory monocytes and contributing to increased levels of inflammatory markers in the blood and tissue^26^. EVs contribute to the SASP secretome and impact T cell activation and interleukin production^27^.

In our study, the EV-based index of age in healthy subjects was driven largely by CD40 and CD44 with a significant axis of variation among individuals aged 60–69 and over 70 years, highlighting their roles as biomarkers of inflammation. CD40, a co-stimulatory protein on antigen-presenting cells, is crucial for their activation and plays a critical role in immune responses and inflammation. Its interaction with CD40 ligand (CD40L) on T cells promotes the production of pro-inflammatory cytokines^28^. CD44, a cell surface glycoprotein, is expressed on various cell types, including leukocytes, and contributes to inflammation by recruiting leukocytes to sites of inflammation and binding to hyaluronic acid and other extracellular matrix components, essential for tissue repair^29^. Finally, platelet-derived EVs with co-stimulatory molecules (CD40L, CD40, OX40L) and functional 20S proteasomes can serve as complete units of antigen presentation. These EVs process exogenous antigens and load peptides onto MHC class I molecules, leading to the proliferation of antigen-specific CD8+ T cells^30^. Our data are further supported by the significant direct correlation of CRP, which is primarily classified as a marker of inflammation, with age in these subjects. However, this correlation becomes unstable in subjects at CV risk or in patients who have experienced an acute CV event, revealing the well-known notion that CRP is modestly related to higher CV risk^31^ and fluctuations in CRP levels among patients with stable ischemic cardiopathy may represent a limiting factor for accurate cardiovascular risk stratification^32^. On the other hand, the multiplexing profile of EVs may unveil multi-factor interactions.

The second biological process we identified is primarily explained by the markers CD8, HLA-I, and HLA-II. This axis can be interpreted as representing both innate and adaptive immune responses and appears to be inversely correlated to the aging process. The innate response includes mast cells, eosinophils, and basophils, which defend the body against immediate infectious agents regardless of prior exposure. The adaptive response, involving T-cells and naïve B-cells, targets specific pathogens and infected cells, eliminating them through a pathogen-specific immunologic response. Additionally, cells involved in adaptive immunity can develop immunologic memory, making subsequent reactions to infections faster and more efficient. This principle is fundamental to vaccine development. There is substantial evidence indicating that the immune response to vaccinations declines with aging, a phenomenon known as immunosenescence^33^. This decline affects both the adaptive and innate immune systems, resulting in weaker responses to vaccines in older adults. Studies specifically focusing on the influenza vaccine have demonstrated effectiveness was only 43% in people over 65 years old compared to 51% in younger adults^34^. This reduced efficacy is attributed to various factors, including decreased antibody responses and impaired T cell function^35^.

Increased secretion of EVs is an universal feature of senescent cells^27,36–38^. Consequently, an overall increase in EVs can be expected in elderly individuals compared to younger ones, as aging organs accumulate senescent cells. However, there is no consensus on whether the number of EVs in different body fluids increases with age. Some studies have shown a higher number of EVs in human plasma when comparing individuals aged 20–30 with those aged 80 years old^39^. Other studies have reported a decrease in EV concentration in human plasma among individuals aged 30–60 years old^40^. Additionally, some research indicates that the concentration of EVs in human serum is not affected by age when comparing adults (20-49 years old) with the elderly (70–104 years old)^41^. In our study, we observed a significant decrease in the pan-EV markers CD9, CD63, and CD81, which are generally assessed as surrogates for EV counts, in healthy subjects with age. This decrease may be attributable to impaired adaptive immunity, including development and activation of B- and T-cells that consistently contribute to blood-derived EV composition^42,43^. Indeed, we found a significant inverse correlation between lymphocytes and age in the overall cohort. Moreover, it has been demonstrated that EVs released from senescent vascular smooth muscle cells cause a decrease in the frequency of activated CD4+ cells (evidenced by a decrease in the number of CD4+CD69+ and CD4+CD38+ cells). These EVs interact with CD3 lymphocytes, stimulating the secretion of IL-17 and promoting THF secretion from CD14+CD16+ monocytes^27^. This supports the active and functional role of circulating EVs in the aging body, acting as a negative feedback mechanism for T cells while positively inducing inflammation in a feedback loop. This makes EV-based biomarkers of especial value as they are part of a pathway causing or reverting aging rather than merely associated with it^17,44^. It has been shown that the total number of EVs did not differ significantly between frail and non-frail individuals. However, frail individuals showed higher amounts of monocyte (CD14+) and natural killer (CD56+)-derived EVs^45^. In our study, the expression of these specific markers did not change in high-risk groups, and only minor differences were noted after stratifying by decades of age or sex.

Finally, we found a peculiar correlation between EV-CD31 levels and CV risk in younger and older patients. Low EV-CD31 levels predicted CV risk in younger patients, while higher levels were linked to increased CV risk in older patients. CD31, primarily expressed on endothelial cells, regulates leukocyte transmigration and endothelial homeostasis^46^. It is involved in both pro- and anti-atherosclerotic processes: evidence suggests that soluble CD31 reflects plaque inflammation^47,48^ while mendelian randomization analyses indicated that soluble CD31 (though not yet defined as associated with circulating EVs or not) is linked to a reduced risk of cardiovascular complications^49^. These data suggest that CD31 serves as both a marker of inflammation and an indicator of endothelial function. Elevated EV-CD31 levels in older patients may reflect plaque inflammation, while lower levels in younger high-risk patients could indicate reduced functionality of endothelial cells.

The primary limitation of our study is that the predictive value of *EVaging* index for both chronological age and age-related CV outcomes was assessed through retrospective analysis; we recognize that, in the context of aging biomarkers, a confirmation should ideally be conducted by prospectively tracking aging-associated outcomes^17^. In addition, *EVaging* was developed to predict demographical age (thus being directly correlated to age), and at the same time it tends to be higher in presence of a CV risk indicator or a worse CV outcome, conditions which are more prevalent in older individuals. Therefore, the possibility of an age-related bias cannot be excluded in this analysis. Nevertheless, our index should be tested in an independent external cohort to be further validated. On the other hand, we are confident in the accuracy and reliability of our analytic methods. This confidence stems from standardized sample collection and storage procedures across our cohorts, as well as the use of a well-established standard measurement multiplex assay, which enhances the precision and reproducibility of the results. Finally, even if a major impact of pharmacological treatment on EV profile was excluded, the rigorous integration of these data would require stepwise multivariable models which would have reduced the statistical power and the reliability of our analyses; potential biases related to daily medications should be addressed in future dedicated studies.

In conclusion, EVs offer several advantages for assessing “inflammaging” in CV disease. As non-invasive biomarkers, EVs can be easily obtained from body fluids like blood, enabling routine monitoring of CV health without invasive procedures. They reflect their cellular origins and provide insights into cellular pathways involved in inflammaging, giving a comprehensive view of the body’s inflammatory and CV health as people age. EVs also respond dynamically to physiological changes, offering early insights in response to treatments or interventions, helping track the impact of drugs or lifestyle changes on inflammaging and CV risk. In perspective, an EV-based biomarker of aging would have the potential of identifying aging-related processes together with health outcomes, and in particular CV comorbidities, whose prevalence increases with age. Further research is necessary to unravel these complexities and enhance the predictive utility of EV profiling in clinical settings.

## Material and method

All relevant data to the present study are available in the online supplement and from the corresponding author upon reasonable request.

### Study cohort

This is an observational cross-sectional cohort study. For each subject included in the analysis, an aliquot of serum was obtained from a biobank created during previous studies involving subjects recruited between March 2017 and July 2022^7,12,50,51^ by different medical centers based in Switzerland and Italy. The following institutions were involved in patient recruitment: Cardiocentro Ticino Institute and Neurocenter of Southern Switzerland (Lugano) and Limmattal Hospital, (Schlieren), in Switzerland; Department of Medical Sciences, Division of Internal Medicine and Hypertension Unit (Torino), in Italy.

All patients gave informed consent according to the Helsinki declaration. An assessment of CV profile was performed for all the study participants, including clinical records for age, sex, blood pressure, BMI, diagnosis of (or treatment for) hypertension, type 2 diabetes, hyperlipidemia, chronic kidney disease (eGFR lower than 60 mL/min according to Cockcroft-Gault equation), established cardiac diseases (coronary artery disease including myocardial infarction, or unstable angina requiring angioplasty, or chronic heart failure with an ejection fraction lower than 35% at echocardiography), organ damage (defined as presence of proteinuria or microalbuminuria with urinary albumin of 30–300 mg/24 h, or albumin-to-creatinine ratio of 30–300 mg/g, and/or left ventricular hypertrophy with a left ventricular mass higher than 50 g/m^2.7^ in men, or 47 g/m^2.7^ in women), biochemical parameters (glucose, lipid profile, creatinine, and complete blood cells count), and 10-year risk of fatal CV events was estimated using the SCORE risk charts of the ESC. The 625 recruited subjects were divided into four groups: (i) HC: subjects without CV risk factors, OD, cardiac disease, or CV events; (ii) Patients with at least a CV risk factor, including hypertension, hyperlipidemia, diabetes, obesity (BMI equal or higher than 30 Kg/sqm), or CKD; (iii) Patients with established cardiac disease and/or OD; (iv) Patients after an acute CV event (myocardial infarction with ST-segment elevation, or a cerebrovascular event). Patients were excluded in case of: (1) Acute/chronic inflammatory disease (acute infections, autoimmune disease); (2) History of, or active cancer; (3) Pregnancy.

### Characterization of circulating EVs

Aliquots of serum were obtained from peripheral venous samples collected in fasting conditions and pre-cleared by serial low-speed centrifugations at 4 °C (1600 × *g* 15 min; 3000 × *g* 20 min; 10,000 × *g* 15 min; 20,000 × *g* 30 min); serum was stored at −80 °C and never thawed before analysis. EV profiling was performed by FC after immuno-capture by fluorescent-labeled (different amounts of PE- phycoerythrin, and FITC- fluorescein isothiocyanate) capture beads against 37 surface antigens carried by circulating vesicles (MACSPlex EV Kit IO human, Miltenyi Biotec), as previously described^7^: 50 uL of serum and 15 uL of capture beads were mixed and diluted to a final volume of 120 uL with MACSPlex buffer. After overnight incubation under gentle agitation, isolated EVs were incubated with detection reagent (APC-, allophycocyanin-conjugated antibodies against CD9-CD63-CD81) and analyzed by FC. Median fluorescence intensity (MFI; arbitrary unit, a.u.) was measured for each EV antigen, and analyzed after normalization by the mean MFI for CD9-CD63-CD81 (normalized MFI, nMFI). To discriminate the different EV antigens, side scatter (SSC) and forward scatter (FSC) were set to confine the analysis to capture beads. FITC and PE voltage were selected to discriminate the different bead subsets, and single bead subsets were each gated to allow the measurement of APC signal (Supplementary Fig. S1).

### Statistical analysis

Variable distribution was evaluated by Kolmogorov–Smirnov test: variables with a normal distribution were expressed as mean ± standard deviation and analyzed by T-student test or one-way ANOVA with post-hoc Bonferroni test; non-normally distributed variables were expressed as median and interquartile range and analyzed by Mann–Whitney of Kruskal–Wallis tests, as appropriated. Ordinal variables were expressed as absolute number and percentage and analyzed by *Chi*-squared or Fisher’s test. Correlations were assessed by Spearman’s Rho test. Univariate and multivariate logistic and linear regression analysis were used to compute odds ratio (OR) and 95% confidence interval (CI), before and after adjustment for age and/or sex. An OR higher than 1 means an increased likelihood of the explored comparator, an OR less than 1 a decreased likelihood. Analysis of ROC curves was performed to evaluate diagnostic performance of EV antigens, through assessment of the area under the curve (AUC) and 95% CI; ROC curves were drawn for EV markers associated to the different CV risk indicators (CV risk factors; cardiac disease and/or OD; acute CV events) independently from age, after stratification for decades of age, in order to discriminate CV outcomes. Principal component analysis was applied to visualize patient distribution according to EV profiling and age stratification in a two-dimensional plot created by unsupervised learning. *P*-values lower than 0.05 were considered significant. *EVaging* index was generated using a support vector regressor with RBF kernel, a supervised machine learning algorithm that builds a function of the scalar input variables while, minimizing the prediction error; the model was trained on the 75% of the overall cohort and tested in the remaining 25%; patients were randomly assigned to training or validation dataset. SPSS Statistics 26 (IBM, USA), Python 3.5 (scikit-learn), and GraphPad PRISM 8.0 (La Jolla, USA) were used for analyses.

## Supplementary information

Supplemental material

## Data Availability

All relevant data to the present study are available in the online supplement and from the corresponding author upon reasonable request.

## Code availability

The underlying code for this study (and training/validation datasets) is not publicly available but may be made available to qualified researchers on reasonable request from the corresponding author.
