# Supplementary material for: Age- and sex-related variations in extracellular vesicle profiling for the assessment of cardiovascular risk: the EVaging index
Source: NPJ Aging. 2024 Dec 19;10(1):63. doi: 10.1038/s41514-024-00189-7 (PMC11659617; doi:10.1038/s41514-024-00189-7)
Supplement: Supplementary file 1 — Supplemental material [file 41514_2024_189_MOESM1_ESM.pdf]

ONLINE SUPPLEMENT

Age- and Sex-related variations in Extracellular Vesicle Profiling for the assessment of Cardiovascular Risk: the EVaging Index

Jacopo Burrello,[1,2] Jessica Goi,[2] Alessio Burrello,[3] Elena Vacchi,[4,5] Azucena Rendon,[1,6] Edoardo Lazzarini,[1,6] Giovanni Bianco,[7] Vittorio Limongelli,[6] Giuseppe Vassalli,[5,8] Carlo W. Cereda,[5,7] Silvia Monticone,[2] Paolo Mulatero,[2] Benedetta Bussolati,[9] Andrea Alimonti,[5,10,11] Giovanni G. Camici,[12] Giorgia Melli,[4,5,7] Elena Osto,[12,13] Giovanni Pedrazzini,[14] Lucio Barile.[1,5,6]

(1) Cardiovascular Theranostics, Istituto Cardiocentro Ticino, Laboratories for Translational Research, Ente Ospedaliero Cantonale, Bellinzona, Switzerland. (2) Division of Internal Medicine and Hypertension Unit, Department of Medical Sciences, University of Torino, Italy. (3) Interuniversity Department of Regional and Urban Studies and Planning, Politecnico di Torino, Italy. (4) Neurodegenerative Diseases Group, Laboratories for Translational Research, Ente Ospedaliero Cantonale, Bellinzona, Switzerland. (5) Faculty of Biomedical Sciences, Università della Svizzera Italiana, Lugano, Switzerland. (6) Euler Institute, Faculty of Biomedical Sciences, Università della Svizzera Italiana, Lugano, Switzerland. (7) Neurology Department, Neurocenter of Southern Switzerland, Ente Ospedaliero Cantonale, Lugano, Switzerland. (8) Cellular and Molecular Cardiology Laboratory, Cardiocentro Ticino Institute, Bellinzona, Switzerland. (9) Department of Molecular Biotechnology and Health Sciences, University of Torino, Italy. (10) Institute of Oncology Research (IOR), Oncology Institute of Southern Switzerland, Bellinzona, Switzerland. (11) Department of Health Sciences and Technology (D-HEST), ETH Zurich, Zurich, Switzerland. (12) Center for Molecular Cardiology, University of Zurich, Schlieren, Switzerland; Department of Research and Education, University Hospital Zurich, Zurich, Switzerland. (13) Division of Physiology and Pathophysiology, Medical University of Graz, Austria. (14) Division of Cardiology Cardiocentro Ticino Institute, Ente Ospedaliero Cantonale Lugano Switzerland.

List of Contents

Figure S1 – Study Design and Main Findings
Figure S2 – Training of *EVaging* index
Figure S3 – Correlation between EV profiling, age, lymphocytes, and C-reactive protein
Figure S4 – Correlations between *EVaging* index and biochemical profile
Figure S5 – Analysis of EV profiling after age- and sex- adjustment

Table S1 – Characteristics of the overall cohort after stratification for age
Table S2 – EV profiling in healthy controls after stratification for age
Table S3 – EV profiling in subjects with cardiovascular risk factors after stratification for age
Table S4 – EV profiling in patients with OD and/or cardiac disease after stratification for age
Table S5 – EV profiling in patients after an acute cardiovascular event after stratification for age
Table S6 – Correlation between EV profiling and age
Table S7 – Assessment of EV biomarkers at unadjusted and age-adjusted regressions
Table S8 – Assessment of EV biomarkers by analysis of ROC curves stratified for age
Table S9 – Characteristics of the overall cohort after stratification for sex
Table S10 – EV profiling in healthy controls after stratification for sex
Table S11 – EV profiling in subjects with cardiovascular risk factors after stratification for sex
Table S12 – EV profiling in patients with OD and/or cardiac disease after stratification for sex
Table S13 – EV profiling in patients after an acute cardiovascular event after stratification for sex
Table S14 – Assessment of EV biomarkers at unadjusted and sex-adjusted regressions
Table S15 – Assessment of EV biomarkers by analysis of ROC curves stratified for sex
Table S16 – Assessment of EV biomarkers at age and sex-adjusted regressions
Table S17 – Assessment of EV biomarkers by analysis of ROC curves stratified for age and sex
Table S18 – Correlation between leucocytes/CRP and age
Table S19 – Correlation between EV profiling (lymphocytes and inflammation markers) and CRP
Table S20 – Correlation between EV profiling (lymphocytes and inflammation markers) and age
Table S21 – Correlation between EV profiling and biochemical parameters
Table S22 – Association of *EVaging* index with CV risk indicators
Table S23 – EV profiling in patients with OD *versus* established cardiac disease
Table S24 – EV profiling after stratification for pharmacological treatment

Figure S1 – Study Design and Main Findings

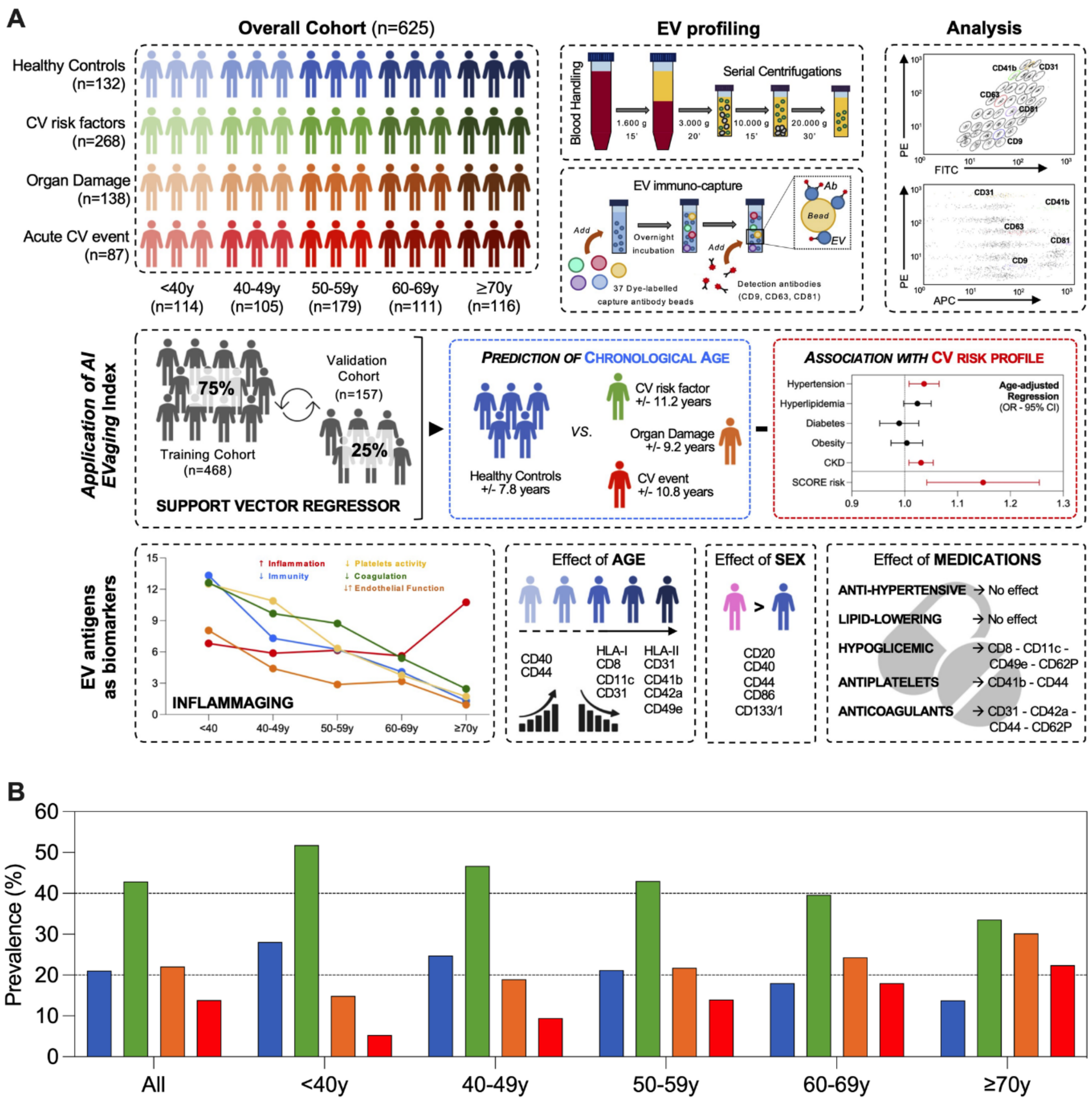

The study included 625 patients (132 healthy controls, blue; 268 patients with a CV risk factor, green; 138 with organ damage and/or cardiac disease, orange; 87 with an acute CV event, red) which were stratified for age. (**A**) An aliquot of serum for each patient was used for EV profiling and flow cytometric analysis (see methods). *EVaging* index was trained on the 75% of the overall cohort and validated in the remaining 25%, through a support vector regressor AI (artificial intelligence) model. *EVaging* accurately predicted chronological age in healthy controls, while its performance worsened in subjects with at least a CV risk factor, organ damage, or an acute CV event; even after correction for age, EVaging was associated to different CV risk indicators, including hypertension, CKD (chronic kidney disease), and the 10-years likelihood of a fatal CV event, as estimated by the SCORE risk of the European Society of Cardiology (ESC). Considering EV surface antigens, the *inflammaging* process increased markers of inflammation, while decreasing those of immunity, platelets, and coagulation. Age, sex, and medications may influence EV levels and should be taken into account when vesicle antigens are used as CV risk biomarkers. (**B**) Prevalence of CV risk indicators across the decades of age.

Figure S2 – Training of *EVaging* index

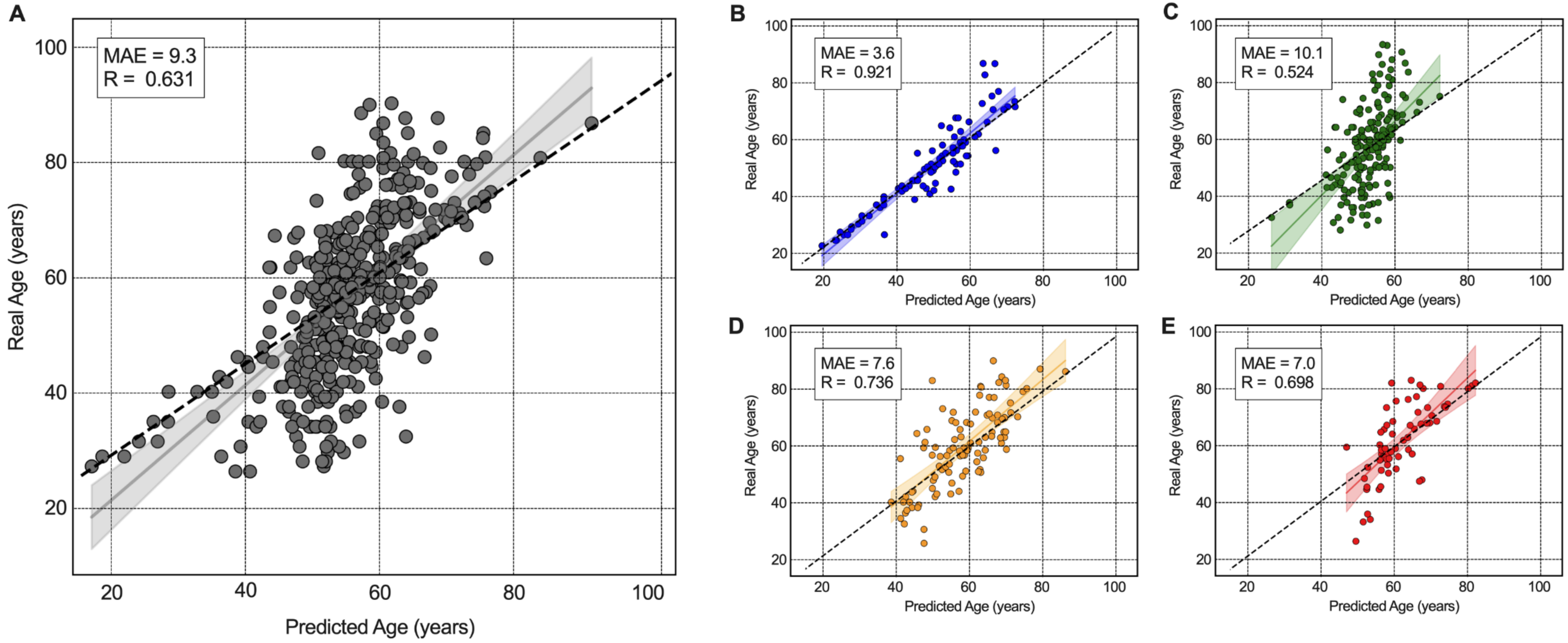

Data from EV profiling were used to train and validate a machine learning model (support vector regressor with RBF kernel) able to predict the age starting from levels of the 37 evaluated EV surface antigens in the overall cohort (**A**) and among healthy controls (**B**), patients with a CV risk factor (**C**), with organ damage and/or cardiac disease (**D**), and those after an acute CV event (**E**). For model generation, 75% of the dataset was used for training and 25% for testing. Prediction curves and 95% confidence intervals, mean absolute error (MAE), and Pearson's R are shown for each model at training. See Figure 1 for the visualization of EVaging performance at model validation.

Figure S3 – Correlation between EV profiling, age, lymphocytes, and C-reactive protein

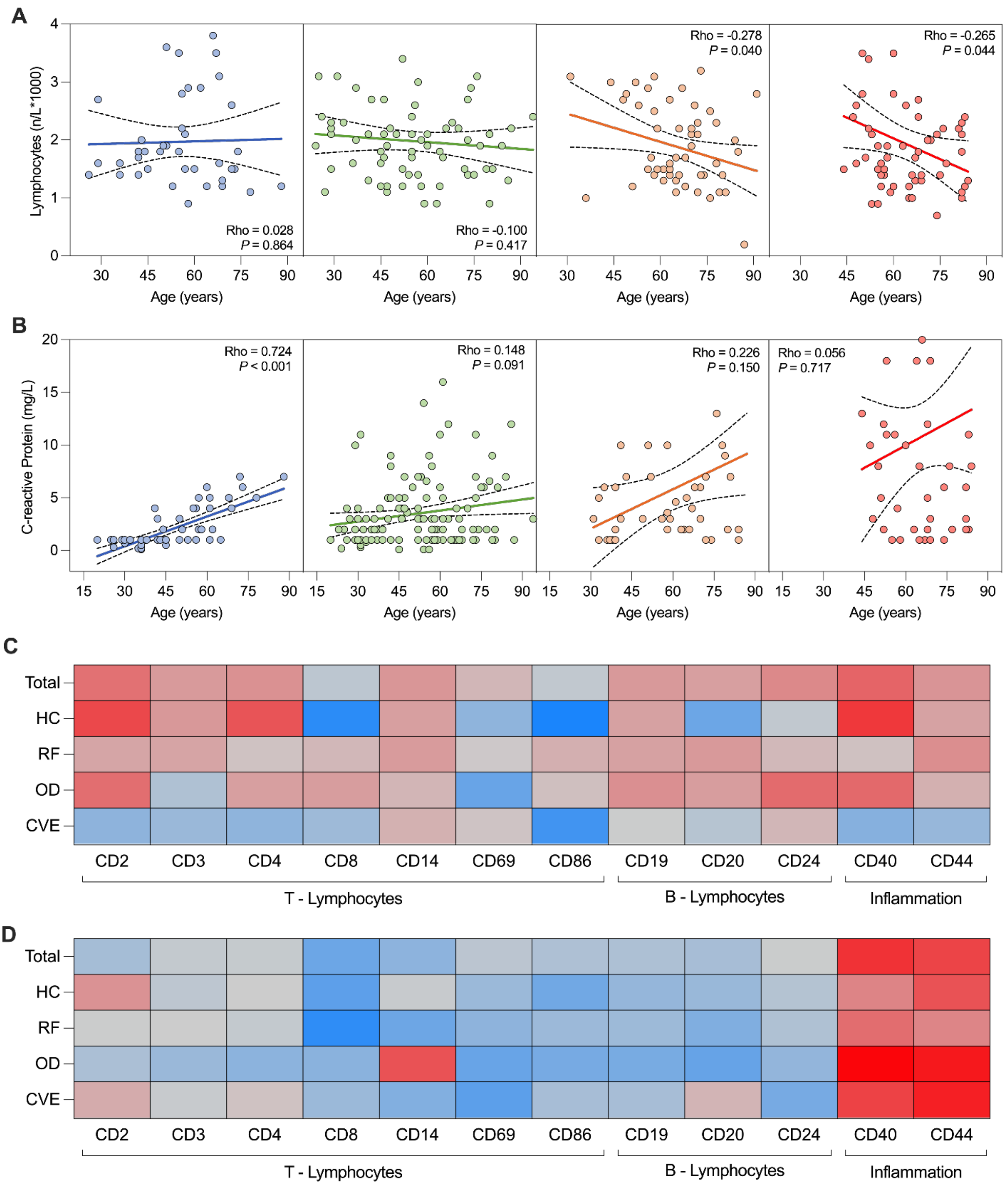

Regression lines and 95% confidence intervals; Rho coefficients and *P*-value are reported for each comparison (see Table S18). (**A**) Correlation between age and lymphocytes in healthy controls (blue), in patients with a CV risk factor (green), with organ damage and/or cardiac disease (orange), or after an acute CV even (red). (**B**) Correlation between age and C-reactive protein. (**C**) Matrix showing correlations between C-reactive protein and EV antigens (scale ranging between blue and red, for inverse and direct correlations, respectively; Table S19). (**D**) Matrix showing correlations between age and EV antigens (Table S20).

Figure S4 – Correlations between *EVaging* index and biochemical profile

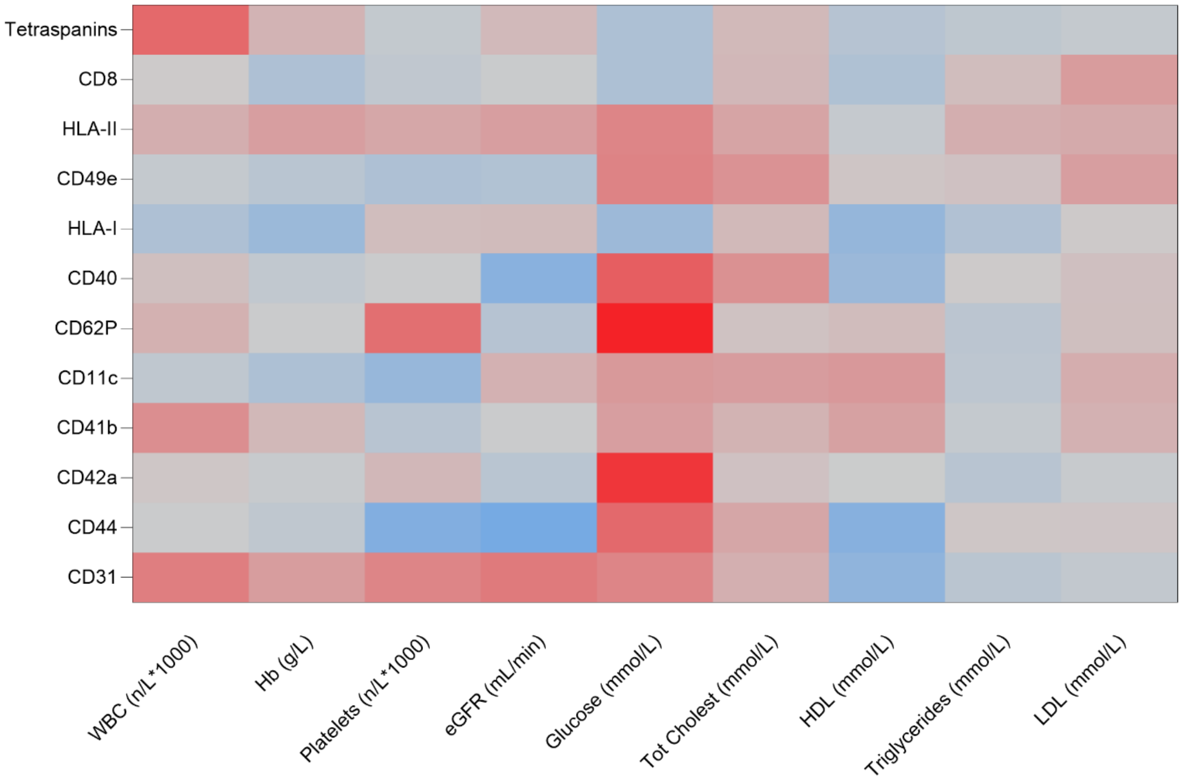

EV antigens differentially expressed in healthy controls were correlated to patient biochemical profile. The matrix shows correlations by Spearman's Rho test (scale ranging between blue and red, for inverse and direct correlations, respectively; see also Table S21). WBC, white blood cells; eGFR, estimated glomerular filtration rate.

Figure S5. Analysis of EV profiling after age- and sex- adjustment

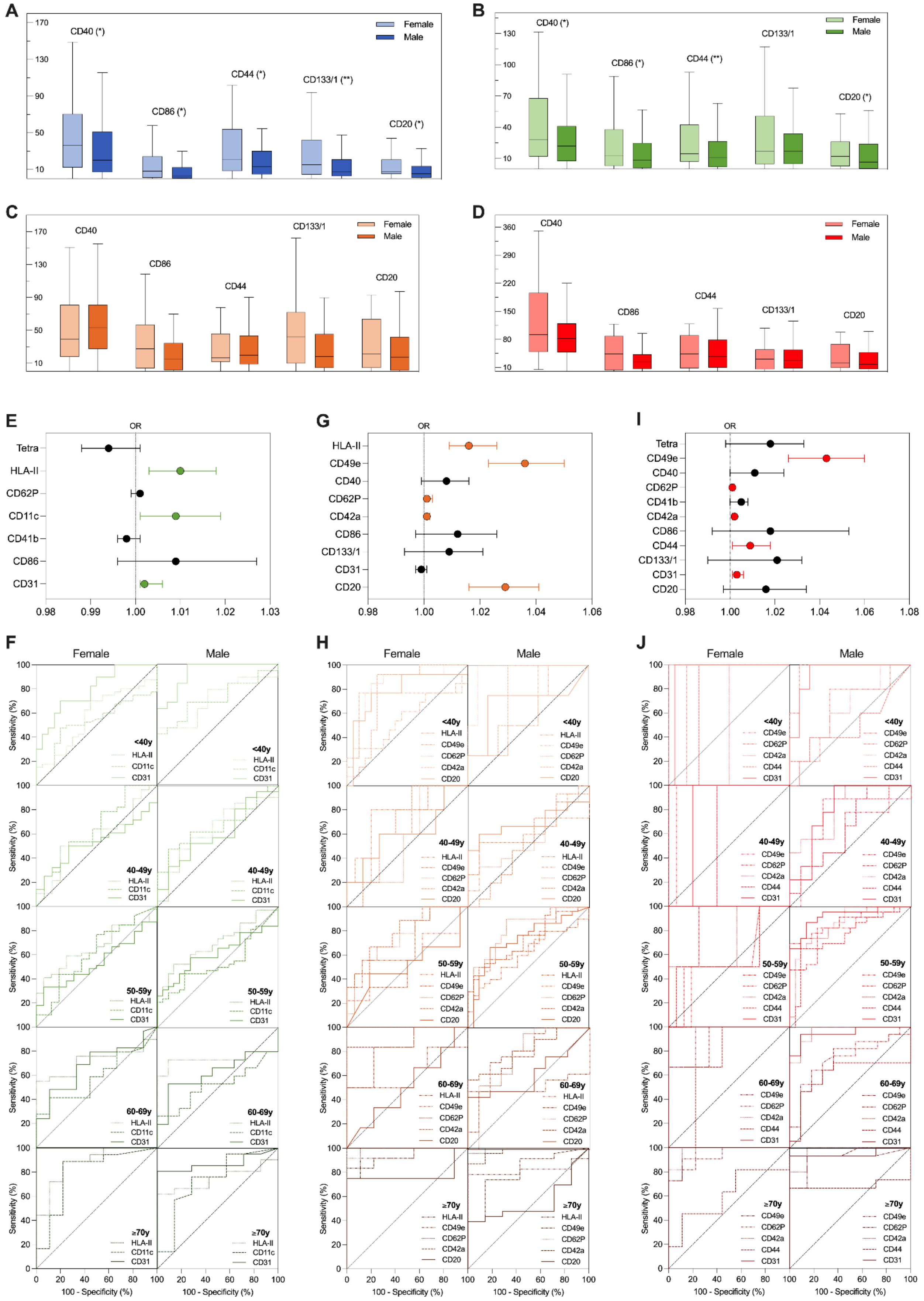

Evaluation of EV surface antigens in the overall cohort (n=625) after stratification for sex. (**A-D**) Levels of EV differentially expressed in HC after stratification for sex. Females and males were compared among HC (blue), subjects with a CV risk factor (green), patients with cardiac disease/OD (orange), or after an acute CV event (red); EV antigens are expressed as nMFI (%). (**E-J**) Multivariate regressions and analysis of ROC curves (after stratification for age and sex) for EV antigens associated with CV risk factors (**E-F**), cardiac disease/OD (**G-H**), or acute CV event (**I-J**); only EV antigens independently associated with CV risk indicators after correction for age and sex, were included in this analysis. *P<0.01; **P<0.05.

Table S1 – Characteristics of the overall cohort after stratification for age

**Variable**	**< 40y** (n=114)	**40-49y** (n=105)	**50-59y** (n=179)	**60-69y** (n=111)	**≥ 70y** (n=116)	***P*-value**
Sex (ref. female)	74 (64.9)	49 (46.7)	66 (36.9)	47 (42.3)	50 (43.1)	**<0.001**
SBP (mmHg)	126 ± 15.6	129 ± 18.1	133 ± 18.5	135 ± 20.1	144 ± 25.3	**<0.001**
DBP (mmHg)	79 ± 10.1	82 ± 10.8	83 ± 11.8	84 ± 14.2	83 ± 11.4	**0.017**
BMI (Kg/sqm)	29.8 ± 6.80	28.0 ± 6.24	26.9 ± 4.85	27.0 ± 5.04	26.1 ± 3.80	**<0.001**
Hypertension (ref. yes)	29 (25.4)	45 (42.9)	83 (46.4)	52 (46.8)	71 (61.2)	**<0.001**
Hyperlipidemia (ref. yes)	46 (40.4)	46 (43.8)	90 (50.3)	64 (57.7)	66 (56.9)	**0.031**
Type 2 Diabetes (ref. yes)	15 (13.2)	11 (10.5)	17 (9.5)	17 (15.3)	20 (17.2)	0.292
Obesity (ref. yes)	44 (38.6)	25 (23.8)	26 (14.5)	24 (21.6)	14 (12.1)	**<0.001**
CKD (ref. yes)	7 (6.1)	4 (3.8)	13 (7.3)	19 (17.1)	20 (17.2)	**<0.001**
Cardiac Disease (ref. yes)	3 (2.6)	11 (10.5)	38 (21.2)	30 (27.0)	37 (31.9)	**<0.001**
Organ damage (ref. yes)	22 (19.3)	19 (18.1)	39 (21.8)	17 (15.3)	32 (27.6)	0.197
Glucose (mmol/L)	5.1 ± 1.24	5.1 ± 1.13	5.6 ± 1.88	6.2 ± 2.42	6.2 ± 1.99	**<0.001**
Tot Cholesterol (mmol/L)	4.6 ± 1.12	5.0 ± 0.99	5.2 ± 1.19	5.1 ± 1.34	5.2 ± 1.29	**0.001**
HDL (mmol/L)	1.3 ± 1.05	1.3 ± 0.44	1.4 ± 0.49	1.4 ± 0.43	1.4 ± 0.55	0.565
Triglycerides (mmol/L)	1.6 ± 1.18	1.5 ± 0.99	1.6 ± 1.12	1.9 ± 1.34	1.8 ± 1.32	0.205
LDL (mmol/L)	2.9 ± 0.97	3.1 ± 0.79	3.1 ± 0.94	3.0 ± 1.08	3.1 ± 1.02	0.269
Creatinine (mg/dL)	0.95 ± 0.585	0.91 ± 0.242	0.99 ± 0.390	1.07 ± 0.624	0.97 ± 0.413	0.108
eGFR (mL/min)	97 ± 24.4	96 ± 21.1	90 ± 23.9	77 ± 23.1	68 ± 19.7	**<0.001**
WBC (n/L*1000)	7.7 ± 2.23	8.8 ± 3.71	8.4 ± 2.99	7.2 ± 2.81	8.0 ± 2.40	0.091
Neutrophils (n/L*1000)	5.0 ± 2.04	6.0 ± 2.99	5.7 ± 2.92	4.7 ± 2.52	5.6 ± 2.38	0.138
Lymphocytes (n/L*1000)	2.1 ± 0.67	2.2 ± 1.15	2.0 ± 0.82	1.9 ± 0.80	1.8 ± 0.61	0.230
Monocytes (n/L*1000)	0.43 ± 0.129	0.42 ± 0.136	0.45 ± 0.152	0.39 ± 0.181	0.44 ± 0.137	0.385
Basophils (n/L*1000)	0.05 ± 0.029	0.05 ± 0.033	0.05 ± 0.029	0.05 ± 0.036	0.04 ± 0.030	0.828
Eosinophils (n/L*1000)	0.18 ± 0.128	0.13 ± 0.085	0.16 ± 0.109	0.13 ± 0.097	0.15 ± 0.115	0.304
C-reactive Protein (mg/L)	2.0 ± 2.08	3.7 ± 3.00	4.5 ± 5.03	6.1 ± 6.54	7.9 ± 9.66	**<0.001**
Hb (g/L)	145 ± 14.2	147 ± 10.2	145 ± 10.3	143 ± 18.9	142 ± 11.3	0.477
Platelets (n/L*1000)	256 ± 43.0	256 ± 60.5	264 ± 63.0	250 ± 65.2	236 ± 57.8	0.160

The table reports characteristics of patients included in the study after stratification for age. A $P<0.05$ was considered significant and showed in bold. SBP, systolic blood pressure; DBP, diastolic blood pressure; CKD, chronic kidney disease (eGFR lower than 60 mL/min); HDL/LDL high/low density lipoproteins; eGFR, estimated glomerular filtration rate; WBC, white blood cells; Hb, haemoglobin.

Table S2 – EV profiling in healthy controls after stratification for age

EV marker	**< 40y** (n=32)			**40-49y** (n=26)			**50-59y** (n=38)			**60-69y** (n=20)			**≥ 70y** (n=16)			***P*-value**
	25th	50th	75th	25th	50th	75th	25th	50th	75th	25th	50th	75th	25th	50th	75th	
Tetraspanins	21.2	32.2	50.6	14.0	34.5	54.4	17.6	23.7	29.5	12.4	14.1	19.0	4.1	7.6	11.7	**<0.001**
CD3	0.0	1.9	14.8	0.0	1.4	8.9	0.1	2.9	11.2	0.0	2.0	6.7	0.0	0.8	4.6	0.494
CD4	0.0	5.1	14.7	0.5	3.8	23.2	1.7	7.7	25.5	2.2	8.9	17.4	0.3	2.0	7.2	0.524
CD19	2.6	14.6	30.2	1.6	7.8	29.5	3.4	14.1	30.0	1.5	13.4	33.7	0.4	2.1	5.8	0.067
CD8	22.8	39.4	50.1	7.1	23.0	32.9	6.7	16.3	43.6	14.2	20.3	35.8	3.6	8.4	26.4	**0.004**
HLA-II	27.6	55.6	82.3	17.8	38.4	50.0	11.1	25.4	36.9	13.7	22.1	26.6	3.0	6.7	10.4	**<0.001**
CD56	0.0	0.0	0.8	0.0	0.3	3.3	0.0	0.5	1.4	0.0	0.0	2.3	0.0	0.1	2.1	0.151
CD105	0.0	0.1	22.9	0.0	2.0	14.8	0.0	10.0	26.7	0.0	2.6	14.6	0.5	7.4	157.6	0.099
CD2	0.0	0.4	5.3	0.0	1.7	8.1	0.5	2.8	12.4	0.0	1.8	7.6	0.1	0.9	3.3	0.145
CD1c	0.0	2.2	12.0	1.4	8.7	16.1	0.8	8.4	21.2	0.9	5.2	9.2	0.1	0.8	10.9	0.144
CD25	0.0	1.9	14.0	0.0	5.4	21.7	0.1	2.1	15.6	0.1	3.5	13.4	0.0	0.6	3.7	0.567
CD49e	16.5	21.2	27.4	2.0	13.2	24.2	2.4	10.9	19.9	2.7	8.9	15.3	0.0	1.2	6.6	**<0.001**
ROR1	0.0	9.0	33.0	0.4	4.7	20.5	1.2	10.8	24.7	2.4	15.6	25.7	0.1	0.7	8.3	0.072
CD209	0.0	1.9	11.9	0.0	3.7	10.0	1.7	8.0	22.4	0.2	3.0	7.4	0.2	2.9	7.6	0.093
CD9	20.8	54.2	105.8	25.1	58.6	80.1	48.2	67.5	89.8	43.2	73.4	114.8	12.4	32.9	88.8	0.299
SSEA-4	5.4	7.1	8.0	0.6	9.0	28.9	2.5	7.0	18.3	6.1	7.0	9.4	6.0	7.6	18.0	0.875
HLA-I	46.1	147.6	497.4	22.9	30.9	44.2	15.6	29.5	42.3	6.9	10.4	24.3	0.5	10.0	14.7	**<0.001**
CD63	28.7	74.5	150.4	33.0	84.6	144.1	37.4	64.7	77.0	59.6	69.3	85.4	35.4	54.2	74.3	0.136
CD40	4.6	19.9	63.3	9.1	22.3	53.7	8.1	24.3	67.3	9.8	18.5	36.4	38.7	51.8	73.3	**0.027**
CD62P	223.6	336.2	487.8	65.7	144.6	341.6	67.4	101.1	229.4	49.7	104.1	224.1	9.3	76.5	129.8	**<0.001**
CD11c	17.1	21.3	30.1	1.8	8.6	23.7	1.0	7.3	20.6	1.3	4.7	9.9	0.0	0.7	6.3	**<0.001**
CD81	101.2	117.7	139.1	54.5	118.2	226.0	68.7	131.0	184.5	118.2	127.5	142.9	111.0	126.3	171.7	0.953
MCSP	0.0	4.5	17.4	0.2	4.4	23.5	1.2	7.9	26.1	0.2	5.2	15.7	0.0	0.7	2.9	0.074
CD146	0.0	0.7	4.9	0.0	1.1	7.7	0.2	2.1	9.9	0.2	3.1	5.2	0.0	0.4	2.5	0.307
CD41b	47.8	105.5	408.7	34.6	61.0	94.7	18.5	38.2	58.3	21.5	30.9	40.5	5.1	9.0	10.5	**<0.001**
CD42a	74.3	141.2	421.1	60.5	154.9	359.9	57.6	123.1	232.0	49.6	116.1	182.0	24.6	49.7	81.4	**0.004**
CD24	0.1	10.4	36.0	2.5	5.4	33.6	3.0	10.5	34.7	0.1	9.7	17.9	1.4	2.7	10.6	0.410
CD86	1.2	11.4	25.5	1.6	7.1	20.4	0.9	5.9	20.6	0.3	6.5	12.1	0.1	1.1	7.1	0.084
CD44	3.8	18.4	37.4	1.1	14.3	35.7	2.2	11.5	30.0	10.7	15.2	20.3	35.2	49.9	68.0	**<0.001**
CD326	0.0	11.4	48.9	0.0	3.3	24.6	0.3	9.2	18.6	0.2	5.8	20.0	0.0	0.3	1.8	0.148
CD133/1	3.4	21.9	50.0	4.8	10.9	31.3	3.0	7.8	19.8	4.4	16.9	26.0	0.4	2.5	14.9	0.058
CD29	21.9	69.1	188.5	15.4	55.3	126.4	22.2	58.6	132.1	34.7	71.5	125.3	7.2	28.8	72.7	0.223
CD69	5.0	22.6	41.5	7.4	15.8	29.5	4.2	17.4	39.2	10.9	21.2	42.8	1.7	3.3	21.8	0.087
CD142	0.0	1.1	13.7	1.1	3.5	13.7	1.0	10.1	19.1	0.0	0.9	6.3	0.5	2.3	10.0	0.152
CD45	0.0	1.8	13.3	0.4	3.9	17.7	1.9	7.8	18.2	0.1	1.8	7.2	0.3	2.3	8.8	0.088
CD31	57.5	160.6	440.8	24.6	44.8	69.0	25.3	34.2	54.9	13.2	28.6	37.7	0.7	6.6	7.8	**<0.001**
CD20	6.2	7.1	16.1	1.8	6.9	20.0	2.2	5.9	19.7	0.4	8.3	16.1	5.2	6.5	7.5	0.675
CD14	0.4	9.7	20.1	3.6	13.8	22.9	2.5	10.5	17.3	3.8	9.3	23.0	5.5	7.1	16.1	0.673

EV surface antigens in healthy controls (n=132), after stratification for age. Levels of EV markers are reported as MFI after normalization by mean MFI of tetraspanins, and expressed as median and interquartile range (25th-50th-75th percentiles). A P<0.05 was considered significant.

Table S3 – EV profiling in subjects with cardiovascular risk factors after stratification for age

EV marker	**< 40y** (n=59)			**40-49y** (n=49)			**50-59y** (n=77)			**60-69y** (n=44)			**≥ 70y** (n=39)			***P*-value**
	25th	50th	75th	25th	50th	75th	25th	50th	75th	25th	50th	75th	25th	50th	75th	
Tetraspanins	10.6	21.4	26.8	3.4	11.7	23.0	5.1	12.3	25.7	6.5	17.8	67.6	6.8	10.7	13.9	**0.012**
CD3	0.0	4.5	17.4	0.4	3.8	13.0	1.5	6.1	23.7	1.2	4.1	13.0	0.9	2.6	10.1	0.230
CD4	2.5	12.6	33.0	1.8	11.4	31.4	4.4	12.4	32.5	2.5	10.5	20.0	3.3	10.3	32.0	0.771
CD19	7.9	17.2	34.1	3.6	16.8	41.0	5.1	15.1	33.9	6.6	13.9	37.3	3.4	11.4	29.6	0.354
CD8	31.4	40.9	52.9	11.7	24.1	49.4	12.3	29.6	52.6	10.2	19.2	39.4	6.5	13.2	37.9	**<0.001**
HLA-II	17.6	36.2	61.2	19.5	40.8	69.9	17.5	41.5	91.0	18.9	42.7	79.6	8.2	13.2	20.4	**<0.001**
CD56	0.0	0.0	2.1	0.0	1.3	6.1	0.0	1.3	13.0	0.0	0.7	7.9	0.0	0.4	6.9	**0.002**
CD105	0.0	4.6	18.7	0.2	10.1	37.2	0.0	11.2	25.1	0.0	0.1	31.5	0.0	4.6	14.0	0.152
CD2	0.0	3.5	19.1	0.2	3.2	15.9	1.0	5.0	22.3	0.2	2.0	13.7	0.8	3.2	10.0	0.479
CD1c	0.8	10.9	22.3	1.8	7.2	22.0	1.7	8.0	25.7	2.0	5.8	15.1	2.8	6.1	18.8	0.812
CD25	0.3	5.8	25.5	0.1	4.0	25.3	0.0	2.3	17.0	0.4	3.7	15.4	0.7	4.0	9.2	0.683
CD49e	0.8	8.4	26.3	3.8	11.8	30.8	2.4	9.7	25.8	1.6	10.6	19.5	2.8	9.2	24.0	0.796
ROR1	0.0	8.2	35.9	2.1	11.3	31.4	4.0	12.2	32.5	2.0	15.2	37.7	2.7	7.1	21.0	0.505
CD209	0.5	6.7	22.6	0.9	4.2	19.8	2.3	7.9	17.7	1.7	7.2	18.0	1.0	4.5	13.4	0.577
CD9	24.2	61.2	84.0	48.8	64.6	90.8	31.2	48.5	87.4	23.2	48.5	105.4	47.7	73.3	116.1	0.196
SSEA-4	0.0	1.9	18.5	0.1	4.1	22.2	2.8	13.0	63.2	0.0	6.6	58.9	0.3	7.5	31.5	**0.004**
HLA-I	17.3	25.1	36.1	9.6	23.3	47.6	4.0	26.9	62.6	3.6	20.1	42.6	7.3	21.3	73.1	0.777
CD63	64.9	105.7	155.3	58.0	89.1	120.8	63.1	85.1	117.1	30.1	70.6	133.6	39.8	69.3	139.4	0.115
CD40	7.1	19.9	31.7	9.4	18.2	61.8	13.1	27.2	69.5	4.9	16.1	46.3	29.5	37.3	72.1	**<0.001**
CD62P	77.3	220.3	643.9	103.5	192.3	482.4	84.6	172.3	344.9	130.3	242.3	297.1	232.3	321.6	413.9	**0.020**
CD11c	1.8	16.9	29.3	1.3	11.0	25.4	2.6	13.9	37.9	1.3	7.0	19.7	1.9	7.1	17.9	0.323
CD81	59.6	85.1	154.0	85.5	117.1	162.0	91.6	136.4	201.8	66.1	133.0	223.9	70.5	127.2	199.3	0.106
MCSP	0.5	7.7	22.0	0.6	7.8	21.8	1.4	7.0	21.3	1.8	6.6	20.2	0.6	3.8	15.8	0.738
CD146	0.0	2.5	11.5	0.0	2.3	8.3	0.9	4.0	14.6	0.0	0.9	4.4	0.6	1.1	4.5	**0.019**
CD41b	25.4	47.2	87.2	33.4	62.6	79.8	26.4	49.0	89.8	24.8	43.8	73.9	24.9	46.5	118.8	0.736
CD42a	83.0	147.3	347.7	84.2	138.0	371.8	80.5	137.5	245.0	107.4	147.4	192.5	166.9	267.6	410.9	**0.001**
CD24	3.1	18.2	37.1	1.7	12.0	38.4	7.0	14.6	35.1	2.1	16.1	44.5	2.4	8.3	14.5	0.173
CD86	3.0	16.2	40.9	0.7	8.6	41.0	3.2	12.4	26.5	1.9	11.0	29.3	2.1	9.7	23.1	0.535
CD44	5.4	10.5	17.7	4.2	12.4	40.4	6.6	11.3	40.9	3.0	9.6	25.2	12.7	40.3	47.8	**0.002**
CD326	0.6	11.0	46.1	0.7	10.9	32.3	0.3	5.6	29.9	0.2	10.1	25.8	0.5	2.5	17.9	0.493
CD133/1	6.1	22.6	54.7	5.7	19.7	36.2	4.2	17.2	42.0	3.4	13.5	42.2	4.3	11.9	30.4	0.523
CD29	25.3	79.3	127.5	39.5	65.1	145.1	20.8	46.6	82.1	11.2	46.2	76.5	45.3	64.1	104.7	**0.005**
CD69	10.9	38.0	62.3	8.0	28.9	47.3	8.2	25.1	45.6	4.1	19.8	56.1	8.4	20.1	32.3	0.396
CD142	0.0	4.7	23.5	0.2	4.0	21.6	2.2	8.0	25.8	1.8	4.7	22.5	1.1	9.6	19.4	0.268
CD45	0.0	5.5	22.0	1.3	10.1	27.7	2.2	10.4	29.6	1.1	8.4	19.8	0.5	7.4	24.4	0.276
CD31	10.5	30.0	64.9	17.1	40.8	65.4	12.6	33.7	66.2	14.4	25.0	45.4	11.3	15.4	19.4	**0.004**
CD20	4.1	15.8	25.9	1.1	10.0	33.8	0.5	11.0	31.2	1.2	9.8	26.3	0.6	5.4	16.5	0.141
CD14	3.0	15.4	43.1	7.6	14.7	35.2	4.3	12.7	27.3	2.0	5.4	18.6	0.0	7.8	17.6	**0.019**

EV surface antigens in patients with CV risk factors (n=268), after stratification for age. Levels of EV markers are reported as MFI after normalization by mean MFI of tetraspanins, and expressed as median and interquartile range (25th-50th-75th percentiles). A P<0.05 was considered significant.

Table S4 – EV profiling in patients with OD and/or cardiac disease after stratification for age

EV marker	**< 40y** (n=17)			**40-49y** (n=20)			**50-59y** (n=39)			**60-69y** (n=27)			**≥ 70y** (n=35)			***P*-value**
	25th	50th	75th	25th	50th	75th	25th	50th	75th	25th	50th	75th	25th	50th	75th	
Tetraspanins	1.9	4.9	21.2	11.0	13.8	34.0	9.3	18.9	41.1	11.1	16.9	27.7	8.4	12.8	15.8	**0.004**
CD3	5.0	23.9	37.9	1.0	14.7	82.5	0.0	2.9	39.1	0.4	2.7	30.4	1.5	7.8	26.7	0.149
CD4	8.8	28.8	45.2	5.1	32.1	92.2	1.4	8.4	80.2	0.3	7.1	32.1	4.8	10.9	37.2	0.210
CD19	15.3	28.0	44.9	5.2	37.1	91.5	4.4	19.0	66.8	6.4	12.4	36.3	3.5	10.6	46.1	0.408
CD8	29.0	39.8	53.6	11.3	38.1	80.4	7.1	19.7	93.0	11.5	27.5	57.2	8.4	22.3	66.7	0.569
HLA-II	24.6	64.3	94.5	14.0	69.4	92.8	13.0	31.8	55.1	11.2	22.7	90.3	12.8	15.9	19.9	**0.008**
CD56	0.0	11.8	17.4	0.0	3.1	21.6	0.6	3.3	17.3	1.1	6.8	15.9	0.1	4.6	22.2	0.962
CD105	0.0	14.9	38.3	0.0	2.1	53.7	0.0	20.5	56.0	0.0	15.1	39.1	0.0	10.9	63.7	0.732
CD2	1.0	6.3	38.8	2.3	12.2	71.5	1.3	12.5	51.7	0.8	6.8	26.4	1.0	5.3	19.4	0.537
CD1c	0.0	14.0	37.5	3.1	14.0	60.0	0.9	8.2	58.8	0.7	6.4	30.6	3.0	8.5	29.6	0.863
CD25	2.3	11.0	27.3	1.0	27.7	38.9	1.2	11.1	35.7	0.8	12.7	20.7	0.5	5.1	36.8	0.882
CD49e	15.2	37.4	54.5	8.4	30.9	59.0	8.5	17.3	54.0	13.3	31.7	78.1	7.2	23.7	48.6	0.345
ROR1	6.5	32.0	56.5	3.5	13.9	68.0	6.0	23.4	57.5	2.0	9.6	45.4	1.0	16.3	56.6	0.467
CD209	4.3	47.7	66.4	2.6	31.9	56.8	2.2	8.3	30.1	1.7	13.8	29.7	1.0	8.0	37.1	0.244
CD9	54.0	75.7	90.5	47.7	59.2	98.9	52.3	76.3	100.6	58.0	73.1	94.1	60.0	87.9	121.5	0.295
SSEA-4	6.1	16.5	46.5	3.5	35.4	66.9	5.5	26.4	63.2	6.6	28.2	75.3	3.0	17.8	65.3	0.911
HLA-I	14.5	24.7	32.0	6.3	38.4	62.4	7.5	24.5	46.7	8.8	21.9	54.0	14.1	33.4	72.7	0.545
CD63	89.7	127.9	160.7	54.0	84.3	140.7	79.3	103.4	156.6	101.8	140.0	165.1	73.9	109.5	143.4	0.249
CD40	13.4	19.7	29.4	13.3	44.6	68.7	26.6	47.2	81.2	25.2	40.0	71.7	53.0	77.7	99.9	**<0.001**
CD62P	84.3	119.7	176.8	101.3	158.7	442.6	128.6	339.9	593.1	283.0	559.2	778.0	295.6	457.2	619.3	**<0.001**
CD11c	12.6	29.5	65.6	4.9	29.8	100.6	1.6	12.6	57.3	0.5	8.8	28.2	4.9	13.9	35.1	0.091
CD81	46.9	81.4	130.5	54.2	126.2	186.5	63.8	109.2	146.1	46.4	77.0	111.9	50.2	98.6	140.5	0.363
MCSP	0.0	11.6	30.0	2.5	21.0	65.6	1.3	13.3	49.6	0.1	6.4	29.2	1.4	19.3	40.2	0.460
CD146	0.5	3.8	17.7	0.5	3.0	14.9	0.3	2.4	23.5	0.1	2.6	8.0	0.4	1.5	8.7	0.790
CD41b	27.6	42.3	66.3	59.1	72.7	110.7	34.0	74.8	112.3	50.1	101.5	135.4	44.0	90.1	158.5	**0.011**
CD42a	62.8	76.0	90.6	127.0	232.8	352.0	146.6	295.6	567.2	187.4	513.2	912.0	389.5	541.2	722.2	**<0.001**
CD24	8.0	27.1	53.8	3.5	16.8	70.7	5.2	20.2	57.6	4.2	14.2	24.0	2.9	22.2	39.9	0.617
CD86	9.0	28.2	60.0	2.5	25.7	61.8	2.0	18.5	52.9	0.5	16.5	36.4	2.1	12.9	31.5	0.220
CD44	10.2	14.6	18.1	3.7	11.9	15.9	3.1	14.3	54.6	15.5	21.9	28.3	20.2	43.4	49.8	**<0.001**
CD326	12.2	45.0	67.0	2.2	15.2	73.3	0.6	7.6	56.9	0.9	17.9	29.3	0.7	5.4	35.0	0.212
CD133/1	29.7	60.1	80.4	6.9	56.5	111.8	3.0	16.3	47.5	6.5	23.1	42.0	6.0	17.7	49.6	**0.038**
CD29	50.6	113.0	165.5	37.8	77.1	134.8	58.7	92.9	126.8	50.0	78.4	182.6	35.4	66.3	120.8	0.611
CD69	27.4	63.8	86.9	7.1	40.4	67.7	10.0	30.1	62.9	10.0	27.1	38.7	8.4	23.5	56.6	0.059
CD142	1.5	11.6	46.0	1.1	22.2	70.8	3.7	22.0	71.4	4.5	18.3	37.8	1.4	7.1	38.3	0.827
CD45	2.1	24.2	39.0	1.6	18.0	55.7	2.0	7.4	51.0	0.1	2.4	44.5	2.2	10.2	38.5	0.602
CD31	14.3	16.9	27.0	17.9	43.1	77.8	45.3	79.2	99.8	38.3	49.8	88.1	27.8	38.8	57.4	**<0.001**
CD20	14.7	28.2	74.8	2.2	26.4	60.7	5.1	18.2	42.8	0.6	12.3	33.2	1.3	10.9	42.0	0.111
CD14	10.5	23.7	40.1	7.1	24.6	96.6	1.4	15.7	43.8	1.5	5.8	31.0	1.5	6.4	24.0	0.082

EV surface antigens in patients with OD and/or cardiac disease (n=138), after stratification for age. EV markers are reported as MFI after normalization by mean MFI of tetraspanins, and expressed as median and interquartile range (25th-50th-75th percentiles). A $P<0.05$ was considered significant.

Table S5 – EV profiling in patients after an acute cardiovascular event after stratification for age

EV marker	**< 40y** (n=6)			**40-49y** (n=10)			**50-59y** (n=25)			**60-69y** (n=20)			**≥ 70y** (n=26)			*P*-value
	25th	50th	75th	25th	50th	75th	25th	50th	75th	25th	50th	75th	25th	50th	75th	
Tetraspanins	54.4	71.9	83.6	38.9	51.4	73.0	50.8	80.0	127.6	38.9	66.0	76.6	20.8	34.5	48.3	**<0.001**
CD3	0.2	26.8	84.2	1.0	4.8	26.0	1.0	33.9	47.9	1.8	16.8	37.0	0.2	14.9	37.0	0.773
CD4	7.0	31.1	126.2	6.5	16.0	39.7	10.4	33.5	79.2	5.8	35.7	102.3	10.1	28.0	43.7	0.693
CD19	13.1	47.8	100.2	9.3	16.1	46.9	7.9	41.5	70.5	8.4	37.0	58.0	6.9	25.6	51.6	0.711
CD8	18.2	34.8	156.7	13.4	33.8	58.7	13.3	43.4	80.5	15.6	35.1	114.3	13.6	22.8	44.3	0.423
HLA-II	57.8	81.9	143.5	11.9	32.5	41.7	13.7	40.8	84.3	25.8	55.1	101.3	12.3	18.9	41.8	**0.002**
CD56	0.0	10.4	28.6	0.3	4.9	6.2	0.8	13.6	30.6	0.0	0.3	7.8	0.0	11.9	44.8	0.190
CD105	0.0	0.0	133.8	12.3	36.6	66.2	17.5	56.8	143.5	0.0	38.2	66.4	0.0	39.4	69.1	0.152
CD2	2.3	17.4	54.8	0.2	1.7	18.6	1.8	45.7	58.4	2.0	15.6	43.5	0.9	28.4	45.5	0.476
CD1c	12.2	40.8	133.9	2.0	7.9	27.4	2.9	31.0	61.5	1.9	12.5	30.4	3.3	29.7	43.8	0.338
CD25	0.3	7.5	22.3	0.3	8.4	19.6	8.5	24.4	50.5	1.1	18.4	40.2	0.3	12.2	44.9	0.175
CD49e	0.9	26.9	30.4	11.7	19.2	33.4	15.5	51.0	72.8	12.9	33.7	69.5	15.1	48.3	65.4	**0.042**
ROR1	7.9	17.7	29.2	18.2	23.8	36.5	17.5	58.4	78.7	12.0	47.5	89.7	11.2	27.6	60.5	0.214
CD209	3.3	14.3	39.1	0.3	4.8	10.6	6.4	27.8	68.8	6.5	25.9	81.3	0.3	5.5	42.8	**0.044**
CD9	49.3	61.0	65.6	36.2	69.8	86.0	54.6	67.7	88.7	33.8	67.9	102.4	61.5	80.3	121.3	0.247
SSEA-4	0.2	61.5	161.5	4.1	18.2	31.4	2.7	20.9	81.9	3.3	18.0	52.3	0.3	17.5	62.5	0.664
HLA-I	0.3	52.9	77.8	3.7	33.0	47.8	28.2	48.8	78.5	11.2	65.0	107.4	24.9	42.0	79.6	0.462
CD63	41.6	68.1	113.3	56.1	91.0	106.1	57.7	93.9	107.1	55.5	84.5	112.1	59.4	113.1	147.4	0.516
CD40	6.5	13.0	32.0	36.3	56.6	170.3	68.5	89.7	137.4	56.1	79.2	102.3	63.5	102.3	226.7	**0.002**
CD62P	38.0	122.5	203.1	156.2	243.1	505.0	114.8	232.9	913.0	272.9	410.8	899.7	366.8	766.2	1606.7	**0.005**
CD11c	1.3	22.5	90.0	2.7	9.6	43.6	8.3	44.0	77.5	4.4	22.2	80.9	11.6	28.8	67.2	0.489
CD81	138.4	194.9	210.2	84.6	124.1	176.5	66.2	112.4	138.3	83.1	127.1	182.4	66.8	91.0	125.5	0.057
MCSP	0.1	7.3	19.7	0.8	11.6	28.6	9.8	25.1	65.4	5.0	24.9	43.3	6.8	25.3	54.1	0.127
CD146	1.5	4.2	25.6	0.9	8.2	13.1	2.4	5.6	14.3	1.2	9.9	29.3	0.9	8.0	31.9	0.991
CD41b	35.4	55.1	122.6	48.6	111.7	201.4	76.7	119.3	175.1	83.1	114.5	181.2	202.6	345.1	474.8	**<0.001**
CD42a	60.7	137.2	254.1	290.3	564.2	889.2	206.8	550.1	1148.3	361.1	479.9	1209.5	235.2	918.6	1651.7	**0.019**
CD24	0.2	3.0	36.3	4.4	16.8	38.6	15.1	39.0	70.5	9.4	26.2	69.7	9.0	33.8	68.0	0.093
CD86	0.1	18.1	31.6	2.9	13.1	41.6	8.2	35.2	60.0	7.2	16.7	55.3	0.8	27.1	55.1	0.618
CD44	0.4	8.1	43.3	8.1	12.5	36.2	8.5	38.0	75.7	10.0	24.2	54.6	35.2	80.6	101.2	**0.006**
CD326	0.4	2.8	24.9	0.5	3.9	25.7	3.3	18.7	71.3	2.1	19.3	46.2	4.4	23.7	51.3	0.298
CD133/1	6.0	32.7	61.6	0.9	21.7	47.6	7.6	31.1	64.3	8.5	27.6	88.6	8.6	35.1	51.7	0.796
CD29	16.5	23.9	42.9	76.0	95.8	117.7	89.8	124.0	228.0	56.6	113.3	145.9	67.1	105.2	321.2	**0.004**
CD69	16.6	26.4	39.6	20.5	36.6	55.6	24.1	43.1	80.9	28.3	48.7	68.4	31.2	55.8	78.0	0.248
CD142	7.7	27.4	45.9	0.1	6.3	17.6	10.5	37.1	83.5	3.3	22.2	59.3	1.7	27.8	45.7	0.057
CD45	6.3	33.1	75.7	14.5	23.2	38.4	17.2	34.0	53.6	6.4	23.0	50.7	10.5	28.1	57.7	0.793
CD31	16.6	29.7	40.3	40.4	75.2	133.9	74.1	111.4	186.2	67.1	98.1	130.8	44.5	68.3	85.1	**<0.001**
CD20	6.8	10.4	34.6	2.3	7.3	18.5	13.5	38.3	62.5	2.2	17.5	34.0	4.1	20.8	69.4	0.101
CD14	18.6	41.8	130.0	4.5	16.4	32.6	4.1	26.9	66.5	6.0	19.1	94.8	0.6	11.2	27.8	0.147

EV surface antigens in patients with an acute CV event (n=87) after stratification for age. Levels of EV markers are reported as MFI after normalization by mean MFI of tetraspanins, and expressed as median and interquartile range (25th-50th-75th percentiles). A $P<0.05$ was considered significant.

Table S6 – Correlation between EV profiling and age

**Spearman Rho [*P*-value]**	**Overall Cohort** (n=625)	**Healthy Controls** (n=132)	**CV Risk Factor** (n=268)	**Organ Damage** (n=138)	**Acute CV Event** (n=87)
Tetraspanins	-0.105<br>0.008	-0.591<br><0.001	-0.069<br>0.263	-0.021<br>0.805	-0.388<br><0.001
CD8	-0.204<br><0.001	-0.237<br>0.006	-0.333<br><0.001	-0.140<br>0.103	-0.104<br>0.339
HLA-II	-0.295<br><0.001	-0.600<br><0.001	-0.187<br>0.002	-0.337<br><0.001	-0.213<br>0.047
CD49e	0.050<br>0.215	-0.359<br><0.001	0.018<br>0.766	0.008<br>0.922	0.194<br>0.071
HLA-I	-0.088<br>0.028	-0.722<br><0.001	-0.032<br>0.599	0.091<br>0.287	0.096<br>0.377
CD40	0.304<br><0.001	0.141<br>0.108	0.187<br>0.002	0.393<br><0.001	0.273<br>0.010
CD62P	0.136<br>0.001	-0.454<br><0.001	0.043<br>0.488	0.432<br><0.001	0.390<br><0.001
CD11c	-0.101<br>0.011	-0.472<br><0.001	-0.120<br>0.052	-0.145<br>0.090	0.066<br>0.543
CD41b	0.048<br>0.232	-0.634<br><0.001	-0.036<br>0.561	0.242<br>0.004	0.517<br><0.001
CD42a	0.257<br><0.001	-0.288<br>0.001	0.174<br>0.004	0.564<br><0.001	0.278<br>0.009
CD44	0.269<br><0.001	0.240<br>0.006	0.140<br>0.022	0.356<br><0.001	0.341<br>0.001
CD31	-0.098<br>0.014	-0.687<br><0.001	-0.145<br>0.018	0.116<br>0.176	-0.043<br>0.693

Correlations between age and EV profiling (EV antigens differentially expressed in healthy controls; see Table S2) were evaluated by Spearman's Rho test in the overall cohort, and among healthy controls, subjects with CV risk factors, organ damage, or with an acute CV event. Rho coefficient (above) and *P*-values (below) are reported for each comparison. $P<0.05$ was considered significant and highlighted in red.

Table S7 – Assessment of EV biomarkers at unadjusted and age-adjusted regressions

EV marker		Unadjusted Analysis			Age-adjusted Analysis		
		OR	95% CI	*P*-value	OR	95% CI	*P*-value
**CV Risk Factor**	Tetraspanins	0.993	0.987-0.999	**0.016**	0.993	0.988-0.999	**0.018**
	CD8	1.009	1.001-1.017	**0.036**	1.011	0.999-1.020	0.054
	HLA-II	1.009	1.003-1.015	**0.005**	1.010	1.004-1.017	**0.002**
	CD49e	1.004	0.995-1.014	0.387	-	-	-
	HLA-I	0.995	0.992-0.998	**<0.001**	0.995	0.992-0.1.004	0.061
	CD40	1.002	0.998-1.006	0.300	-	-	-
	CD62P	1.001	1.001-1.004	**0.026**	1.001	1.002-1.005	**0.006**
	CD11c	1.010	1.001-1.019	**0.049**	1.011	1.001-1.021	**0.028**
	CD41b	1.002	1.000-1.003	**0.034**	1.003	1.001-1.010	**0.046**
	CD42a	1.000	1.000-1.001	0.471	-	-	-
	CD44	0.997	0.991-1.002	0.247	-	-	-
	CD31	1.005	1.002-1.006	**0.001**	1.003	1.001-1.007	**0.008**
**OD & Cardiac Disease**	Tetraspanins	0.996	0.990-1.002	0.201	-	-	-
	CD8	1.012	1.005-1.020	**0.001**	1.015	0.997-1.023	0.071
	HLA-II	1.007	1.001-1.013	**0.044**	1.017	1.008-1.025	**<0.001**
	CD49e	1.030	1.018-1.042	**<0.001**	1.035	1.022-1.049	**<0.001**
	HLA-I	0.996	0.993-0.999	**0.010**	0.998	0.995-1.000	0.098
	CD40	1.011	1.005-1.018	**0.001**	1.009	0.993-1.016	0.077
	CD62P	1.002	1.001-1.003	**0.006**	1.002	1.001-1.003	**0.008**
	CD11c	1.020	1.020-1.030	**<0.001**	1.026	0.991-1.037	0.101
	CD41b	1.000	0.998-1.002	0.887	-	-	-
	CD42a	1.002	1.001-1.003	**0.002**	1.002	1.001-1.003	**0.005**
	CD44	1.998	1.991-1.006	0.627	-	-	-
	CD31	1.008	1.001-1.017	**0.043**	1.007	1.002-1.021	**0.032**
**Acute CV Event**	Tetraspanins	1.018	1.010-1.027	**<0.001**	1.025	1.015-1.035	**<0.001**
	CD8	1.016	1.007-1.025	**<0.001**	1.010	1.000-1.022	0.052
	HLA-II	1.011	1.004-1.019	**0.004**	1.014	0.998-1.035	0.075
	CD49e	1.039	1.026-1.053	**<0.001**	1.041	1.026-1.056	**<0.001**
	HLA-I	0.999	0.997-1.001	0.371	-	-	-
	CD40	1.021	1.013-1.028	**<0.001**	1.011	0.998-1.026	0.064
	CD62P	1.002	1.001-1.003	**<0.001**	1.002	1.001-1.003	**0.001**
	CD11c	1.029	1.018-1.041	**<0.001**	1.015	0.990-1.047	0.096
	CD41b	1.005	1.003-1.007	**<0.001**	1.005	1.003-1.007	**<0.001**
	CD42a	1.002	1.001-1.003	**<0.001**	1.002	1.001-1.003	**<0.001**
	CD44	1.011	1.004-1.018	**0.003**	1.007	0.999-1.014	0.062
	CD31	1.001	1.000-1.003	**0.047**	1.004	1.001-1.009	**0.024**

Univariate and age-adjusted multivariate logistic regressions to assess association between EV antigens differentially expressed in healthy controls after stratification for age (see Table S2) and presence of a CV risk factors, or OD/cardiac disease, or an acute CV event. The group of healthy controls was used as comparator. The table reports odds ratio (OR) and 95% confidence interval (95% CI); $P<0.05$ was considered significant and showed in bold. An OR greater than 1 indicates an increased likelihood of a CV risk factor, OD/cardiac disease, or an acute CV event, and an OR less than 1 a decreased likelihood.

Table S8 – Assessment of EV biomarkers by analysis of ROC curves stratified for age

EV marker		< 40y			40-49y			50-59y			60-69y			≥ 70y		
		AUC	95% CI	*P*-value	AUC	95% CI	*P*-value	AUC	95% CI	*P*-value	AUC	95% CI	*P*-value	AUC	95% CI	*P*-value
**CV Risk Factor**	Tetraspanins	0.771*	0.672-0.869	**<0.001**	0.799*	0.700-0.898	**<0.001**	0.715*	0.624-0.806	**<0.001**	0.518	0.377-0.659	0.817	0.666	0.507-0.824	0.055
	CD8	0.554	0.428-0.680	0.399	0.594	0.462-0.727	0.182	0.612	0.502-0.722	**0.048**	0.564*	0.422-0.707	0.413	0.604	0.439-0.770	0.228
	HLA-II	0.620*	0.320-0.572	0.060	0.527	0.389-0.664	0.705	0.685	0.589-0.781	**0.001**	0.714	0.591-0.837	**0.006**	0.808	0.693-0.923	**<0.001**
	CD49e	0.659*	0.546-0.772	**0.013**	0.521	0.382-0.660	0.764	0.506	0.395-0.618	0.915	0.537	0.385-0.689	0.638	0.766	0.629-0.903	**0.002**
	HLA-I	0.877*	0.799-0.954	**<0.001**	0.615*	0.490-0.740	0.102	0.536*	0.433-0.639	0.529	0.530	0.383-0.677	0.701	0.724	0.591-0.856	**0.010**
	CD40	0.557*	0.420-0.695	0.369	0.524	0.389-0.659	0.738	0.542	0.429-0.656	0.463	0.509*	0.358-0.661	0.908	0.612*	0.465-0.760	0.195
	CD62P	0.594*	0.478-0.710	0.141	0.571	0.433-0.709	0.311	0.619	0.509-0.730	**0.038**	0.706	0.548-0.863	**0.009**	0.946	0.880-1.000	**<0.001**
	CD11c	0.588*	0.470-0.706	0.168	0.517*	0.379-0.655	0.811	0.598	0.492-0.704	0.088	0.572	0.433-0.711	0.358	0.764	0.612-0.916	**0.002**
	CD41b	0.746*	0.639-0.853	**<0.001**	0.520*	0.382-0.659	0.772	0.607	0.504-0.710	0.063	0.632	0.497-0.768	0.092	0.911	0.830-0.992	**<0.001**
	CD42a	0.524*	0.398-0.650	0.708	0.504*	0.358-0.650	0.956	0.536	0.422-0.650	0.530	0.640	0.481-0.798	0.075	0.947	0.883-1.000	**<0.001**
	CD44	0.598*	0.466-0.729	0.125	0.540	0.397-0.683	0.574	0.549	0.437-0.662	0.390	0.642*	0.503-0.781	0.070	0.708*	0.564-0.851	**0.016**
	CD31	0.851*	0.771-0.930	**<0.001**	0.572*	0.442-0.702	0.306	0.533*	0.429-0.637	0.568	0.534	0.390-0.678	0.664	0.939	0.875-1.000	**<0.001**
**OD & Cardiac Disease**	Tetraspanins	0.840*	0.690-0.990	**<0.001**	0.673*	0.506-0.840	**0.046**	0.620*	0.491-0.749	0.071	0.562	0.392-0.732	0.471	0.717	0.572-0.861	**0.014**
	CD8	0.539	0.363-0.714	0.659	0.650	0.480-0.820	0.084	0.567	0.436-0.698	0.313	0.530	0.363-0.696	0.731	0.684	0.527-0.841	**0.037**
	HLA-II	0.511	0.329-0.693	0.900	0.631	0.449-0.812	0.132	0.588	0.459-0.718	0.182	0.569	0.396-0.741	0.426	0.880	0.788-0.973	**<0.001**
	CD49e	0.676	0.493-0.860	0.054	0.681	0.521-0.840	**0.037**	0.650	0.528-0.773	**0.023**	0.820	0.697-0.944	**<0.001**	0.861	0.758-0.964	**<0.001**
	HLA-I	0.890*	0.801-0.978	**<0.001**	0.535	0.345-0.724	0.690	0.542*	0.409-0.675	0.528	0.607	0.439-0.776	0.212	0.846	0.742-0.951	**<0.001**
	CD40	0.523*	0.362-0.684	0.793	0.631	0.467-0.794	0.132	0.659	0.536-0.783	**0.016**	0.722	0.570-0.874	**0.010**	0.696	0.545-0.848	**0.026**
	CD62P	0.862*	0.748-0.976	**<0.001**	0.546	0.375-0.717	0.595	0.765	0.659-0.871	**0.000**	0.869	0.752-0.985	**<0.001**	0.979	0.944-1.000	**<0.001**
	CD11c	0.626	0.442-0.810	0.150	0.635	0.463-0.806	0.121	0.587	0.458-0.716	0.189	0.578	0.413-0.743	0.366	0.830	0.712-0.949	**<0.001**
	CD41b	0.787*	0.662-0.911	**0.001**	0.604	0.434-0.774	0.231	0.727	0.611-0.843	**0.001**	0.880	0.784-0.975	**<0.001**	0.959	0.903-1.000	**<0.001**
	CD42a	0.765*	0.630-0.899	**0.002**	0.588	0.423-0.754	0.308	0.734	0.623-0.845	**<0.001**	0.872	0.775-0.970	**<0.001**	0.988	0.966-1.000	**<0.001**
	CD44	0.544*	0.379-0.709	0.614	0.552*	0.379-0.725	0.550	0.554	0.424-0.683	0.418	0.654	0.484-0.823	0.074	0.654*	0.492-0.815	0.081
	CD31	0.967*	0.925-1.000	**<0.001**	0.502*	0.325-0.679	0.982	0.742	0.623-0.860	**<0.001**	0.815	0.692-0.938	**<0.001**	0.984	0.951-1.000	**<0.001**
**Acute CV Event**	Tetraspanins	0.823	0.653-0.993	**0.013**	0.688	0.507-0.870	0.084	0.889	0.803-0.976	**<0.001**	0.988	0.963-1.000	**<0.001**	0.950	0.874-1.000	**<0.001**
	CD8	0.562	0.266-0.859	0.631	0.677	0.475-0.878	0.104	0.669	0.527-0.810	**0.024**	0.648	0.469-0.826	0.110	0.731	0.564-0.898	**0.013**
	HLA-II	0.719	0.500-0.938	0.093	0.606*	0.409-0.803	0.331	0.666	0.518-0.814	**0.026**	0.827	0.687-0.968	**<0.001**	0.877	0.770-0.985	**<0.001**
	CD49e	0.529	0.241-0.817	0.826	0.631	0.437-0.824	0.230	0.790	0.674-0.906	**<0.001**	0.816	0.684-0.949	**0.001**	0.925	0.843-1.000	**<0.001**
	HLA-I	0.781*	0.613-0.950	**0.031**	0.542*	0.293-0.792	0.698	0.734	0.601-0.866	**0.002**	0.706	0.530-0.883	**0.026**	0.911	0.812-1.000	**<0.001**
	CD40	0.583*	0.393-0.774	0.522	0.773	0.610-0.936	**0.012**	0.827	0.720-0.934	**<0.001**	0.833	0.693-0.972	**<0.001**	0.798	0.662-0.934	**0.001**
	CD62P	0.906*	0.810-1.000	**0.002**	0.600	0.396-0.804	0.358	0.726	0.596-0.856	**0.003**	0.780	0.630-0.930	**0.002**	0.953	0.896-1.000	**<0.001**
	CD11c	0.529	0.164-0.893	0.826	0.562	0.344-0.779	0.572	0.762	0.636-0.889	**<0.001**	0.740	0.573-0.907	**0.009**	0.863	0.750-0.976	**<0.001**
	CD41b	0.698*	0.510-0.885	0.128	0.715	0.513-0.917	**0.048**	0.916	0.829-1.000	**<0.001**	0.903	0.792-1.000	**<0.001**	1.000	1.000-1.000	**<0.001**
	CD42a	0.578*	0.361-0.796	0.548	0.769	0.608-0.930	**0.013**	0.845	0.751-0.940	**<0.001**	0.975	0.934-1.000	**<0.001**	0.971	0.928-1.000	**<0.001**
	CD44	0.570*	0.303-0.838	0.589	0.552	0.356-0.748	0.634	0.711	0.576-0.845	**0.005**	0.612	0.424-0.801	0.224	0.623	0.454-0.791	0.187
	CD31	0.922*	0.835-1.000	**0.001**	0.735	0.556-0.913	**0.031**	0.926	0.857-0.995	**<0.001**	0.968	0.916-1.000	**<0.001**	0.978	0.935-1.000	**<0.001**

Analyses of ROC curves were performed to evaluate diagnostic performance of EV antigens in the discrimination of CV risk factors, or OD/cardiac disease, or acute CV events, from healthy subjects, used as controls. The table reports the area under the curve (AUC) and 95% confidence interval (95% CI) after stratification for age. $P<0.05$ was considered significant and showed in bold (*AUC computed for threshold less than X).

Table S9 – Characteristics of the overall cohort after stratification for sex

**Variable**	**Female** (n=286)	**Male** (n=339)	***P*-value**
Age (years)	53 ± 16.9	57 ± 14.5	**0.001**
SBP (mmHg)	130 ± 19.6	137 ± 20.9	**<0.001**
DBP (mmHg)	80 ± 11.2	84 ± 12.0	**<0.001**
BMI (Kg/sqm)	28.3 ± 6.41	26.8 ± 4.50	**0.001**
Hypertension (ref. yes)	109 (38.1)	171 (50.4)	**0.002**
Hyperlipidemia (ref. yes)	128 (44.8)	184 (54.3)	**0.018**
Type 2 Diabetes (ref. yes)	34 (11.9)	46 (13.6)	0.531
Obesity (ref. yes)	82 (28.7)	51 (15.0)	**<0.001**
CKD (ref. yes)	31 (10.8)	32 (9.4)	0.563
Cardiac Disease (ref. yes)	28 (9.8)	91 (26.8)	**<0.001**
Organ damage (ref. yes)	44 (15.4)	85 (25.1)	**0.003**
Glucose (mmol/L)	5.3 ± 1.25	6.0 ± 2.20	**<0.001**
Tot Cholesterol (mmol/L)	5.1 ± 1.27	5.1 ± 1.16	0.925
HDL (mmol/L)	1.4 ± 1.77	1.3 ± 0.48	0.060
Triglycerides (mmol/L)	1.7 ± 1.28	1.7 ± 1.12	0.870
LDL (mmol/L)	3.1 ± 0.94	3.0 ± 0.98	0.631
Creatinine (mg/dL)	0.89 ± 0.488	1.05 ± 0.434	**<0.001**
eGFR (mL/min)	83 ± 24.7	88 ± 25.1	**0.004**
WBC (n/L*1000)	7.8 ± 2.73	8.1 ± 2.96	0.435
Neutrophils (n/L*1000)	5.2 ± 2.58	5.6 ± 2.68	0.233
Lymphocytes (n/L*1000)	2.0 ± 0.72	1.9 ± 0.86	0.271
Monocytes (n/L*1000)	0.42 ± 0.142	0.43 ± 0.158	0.612
Basophils (n/L*1000)	0.05 ± 0.034	0.05 ± 0.030	0.522
Eosinophils (n/L*1000)	0.15 ± 0.114	0.14 ± 0.102	0.572
C-reactive Protein (mg/L)	3.7 ± 3.75	6.2 ± 8.23	**0.001**
Hb (g/L)	140 ± 14.4	146 ± 12.2	**0.002**
Platelets (n/L*1000)	265 ± 59.3	242 ± 59.9	**0.006**

The table reports characteristics of patients included in the study after stratification for sex. A $P<0.05$ was considered significant and showed in bold. SBP, systolic blood pressure; DBP, diastolic blood pressure; CKD, chronic kidney disease (eGFR lower than 60 mL/min); HDL/LDL high/low density lipoproteins; eGFR, estimated glomerular filtration rate; WBC, white blood cells; Hb, haemoglobin.

Table S10 – EV profiling in healthy controls after stratification for sex

EV marker	**Female** (n=69)			**Male** (n=63)			***P*-value**
	25th	50th	75th	25th	50th	75th	
Tetraspanins	12.3	22.3	35.2	14.1	21.2	37.9	0.548
CD3	0.0	1.0	11.5	0.2	2.5	7.1	0.419
CD4	0.1	4.8	27.0	0.9	7.2	15.6	0.864
CD19	2.5	14.3	30.7	1.5	5.2	28.5	0.166
CD8	14.1	24.2	47.6	9.0	17.1	38.3	0.096
HLA-II	12.4	34.0	49.1	11.5	23.8	36.8	0.259
CD56	0.0	0.0	1.3	0.0	0.2	1.9	0.267
CD105	0.0	2.6	21.2	0.0	5.1	24.2	0.347
CD2	0.0	1.0	5.4	0.3	2.3	8.3	0.120
CD1c	0.5	5.2	15.1	0.7	5.3	12.9	0.918
CD25	0.0	3.1	15.8	0.1	2.2	13.4	0.599
CD49e	2.6	17.5	25.4	2.7	11.1	18.8	0.357
ROR1	0.3	10.7	26.9	1.2	6.5	20.6	0.877
CD209	0.0	4.0	12.5	0.5	3.5	8.9	0.894
CD9	32.9	59.1	82.1	25.3	65.5	97.9	0.534
SSEA-4	4.8	7.2	11.5	4.0	6.8	17.3	0.719
HLA-I	12.0	27.4	60.0	13.3	30.0	53.9	0.915
CD63	40.3	69.2	121.7	39.2	67.0	85.5	0.406
CD40	12.1	36.0	70.6	7.0	20.0	51.1	**0.046**
CD62P	73.8	181.4	341.7	67.9	114.6	277.2	0.159
CD11c	1.2	10.2	23.0	2.0	7.4	20.2	0.288
CD81	82.7	117.6	150.2	100.9	132.3	176.1	0.100
MCSP	0.1	4.5	22.7	0.2	3.1	15.7	0.592
CD146	0.0	0.6	6.5	0.2	2.7	5.5	0.118
CD41b	24.6	41.4	85.6	14.7	37.4	67.1	0.260
CD42a	47.0	112.0	245.5	55.3	139.7	247.6	0.279
CD24	1.0	10.2	39.3	1.9	5.3	19.5	0.177
CD86	1.0	8.3	24.3	0.4	3.0	12.4	**0.043**
CD44	8.3	20.6	54.0	4.7	13.3	30.2	**0.048**
CD326	0.0	13.1	34.3	0.1	1.5	14.0	0.067
CD133/1	4.4	15.3	42.4	2.9	7.7	21.1	**0.007**
CD29	34.5	68.9	144.8	16.5	35.8	102.0	0.083
CD69	4.5	20.4	42.5	4.2	13.1	33.8	0.263
CD142	0.0	3.7	15.9	0.1	3.8	11.3	0.952
CD45	0.0	3.0	10.9	0.6	4.7	15.2	0.319
CD31	16.4	42.8	67.7	18.3	34.0	71.8	0.741
CD20	5.1	7.3	20.9	1.1	5.3	13.8	**0.012**
CD14	2.7	10.9	27.4	3.7	10.9	15.9	0.288

EV surface antigens in healthy controls (n=132), after stratification for sex. Levels of EV markers are reported as MFI after normalization by mean MFI of tetraspanins, and expressed as median and interquartile range (25th-50th-75th percentiles). A P<0.05 was considered significant.

Table S11 – EV profiling in subjects with CV risk factors after stratification for sex

EV marker	**Female** (n=154)			**Male** (n=114)			***P*-value**
	25th	50th	75th	25th	50th	75th	
Tetraspanins	4.3	11.0	23.4	7.8	16.0	28.0	**0.002**
CD3	0.7	4.8	18.5	0.9	4.1	14.4	0.787
CD4	3.1	11.1	35.2	2.7	11.0	24.4	0.268
CD19	5.8	14.3	34.0	5.1	17.9	33.7	0.782
CD8	12.8	30.4	47.2	11.2	29.7	45.3	0.483
HLA-II	16.1	36.4	67.5	13.0	32.7	62.6	0.607
CD56	0.0	0.4	6.8	0.0	0.5	8.1	0.343
CD105	0.0	5.1	22.5	0.0	6.5	24.4	0.966
CD2	0.3	5.0	16.4	0.5	3.3	15.8	0.938
CD1c	2.0	7.9	21.3	1.7	6.4	20.7	0.735
CD25	0.3	4.4	17.9	0.1	4.0	16.1	0.638
CD49e	1.6	10.8	24.8	2.8	9.1	24.5	0.815
ROR1	2.0	10.1	29.8	3.2	10.9	32.8	0.295
CD209	1.0	5.3	18.1	1.8	7.6	16.8	0.439
CD9	38.0	63.7	92.9	20.1	51.5	84.9	**0.035**
SSEA-4	0.1	7.2	23.0	0.1	7.5	33.8	0.368
HLA-I	8.5	26.0	57.6	6.9	20.3	53.0	0.169
CD63	53.8	93.1	144.8	49.1	75.2	109.7	**0.009**
CD40	12.0	27.9	67.8	7.5	22.0	41.1	**0.030**
CD62P	151.6	256.4	558.3	78.7	160.7	287.8	**<0.001**
CD11c	1.8	12.5	32.9	1.8	9.0	24.4	0.477
CD81	67.4	116.9	169.3	76.6	131.8	209.2	**0.042**
MCSP	0.7	7.9	21.8	1.5	6.2	19.2	0.996
CD146	0.0	2.2	8.1	0.3	1.9	9.4	0.987
CD41b	25.9	52.7	92.8	25.9	46.2	82.3	0.399
CD42a	87.6	156.8	348.7	96.3	145.7	270.6	0.400
CD24	3.2	14.4	41.9	2.8	11.2	28.7	0.208
CD86	3.0	12.9	38.0	1.0	8.2	24.9	**0.016**
CD44	7.1	14.7	42.5	1.8	11.1	26.7	**0.005**
CD326	1.0	13.2	37.0	0.3	2.9	21.2	**0.001**
CD133/1	4.7	17.2	50.8	4.8	16.9	34.1	0.274
CD29	31.7	65.0	128.5	20.4	49.8	89.0	**0.013**
CD69	8.4	23.3	54.1	6.9	24.6	43.5	0.304
CD142	0.7	5.8	22.6	1.0	5.8	19.6	0.920
CD45	1.9	10.1	26.0	0.9	6.4	22.0	0.153
CD31	16.0	35.9	67.9	10.7	19.8	41.7	**<0.001**
CD20	2.6	12.1	26.2	0.1	6.6	24.1	**0.037**
CD14	3.1	13.8	33.1	3.5	12.2	20.7	0.219

EV surface antigens in patients with CV risk factors (n=268), after stratification for sex. Levels of EV markers are reported as MFI after normalization by mean MFI of tetraspanins, and expressed as median and interquartile range (25th-50th-75th percentiles). A $P<0.05$ was considered significant.

Table S12 – EV profiling in patients with OD and/or cardiac disease after stratification for sex

EV marker	**Female** (n=45)			**Male** (n=93)			***P*-value**
	25th	50th	75th	25th	50th	75th	
Tetraspanins	6.1	12.4	25.5	9.2	14.6	25.2	0.303
CD3	1.4	8.7	39.4	0.4	5.4	31.4	0.402
CD4	4.7	16.7	57.4	2.0	9.6	47.7	0.263
CD19	9.4	28.0	58.5	4.2	14.2	54.4	0.100
CD8	12.5	30.4	68.9	8.3	24.1	74.6	0.387
HLA-II	13.9	34.8	76.3	11.9	22.7	67.8	0.240
CD56	0.3	5.4	18.3	0.1	4.6	16.7	0.636
CD105	0.0	10.5	43.8	0.0	15.1	53.1	0.509
CD2	1.0	5.1	38.8	1.1	9.1	39.6	0.576
CD1c	1.7	11.1	44.0	1.0	8.3	34.1	0.771
CD25	1.5	15.4	33.8	0.7	9.7	33.0	0.592
CD49e	14.5	35.3	60.8	9.9	22.0	49.3	0.172
ROR1	7.0	32.0	65.7	1.0	15.0	45.7	**0.014**
CD209	3.8	16.0	53.3	1.3	8.9	36.8	0.112
CD9	53.7	78.0	102.5	54.6	76.9	100.8	0.780
SSEA-4	5.5	17.8	54.2	3.8	28.2	67.1	0.457
HLA-I	11.1	24.5	45.8	9.5	31.1	59.9	0.429
CD63	75.1	113.1	168.1	75.9	107.9	145.2	0.253
CD40	17.8	39.5	81.0	27.4	53.0	81.2	0.347
CD62P	156.7	295.6	613.7	150.0	339.9	603.0	0.829
CD11c	3.9	13.9	55.0	2.7	13.7	58.8	0.650
CD81	42.1	81.4	143.3	56.5	102.0	139.7	0.308
MCSP	1.7	14.2	46.5	1.0	10.1	37.6	0.508
CD146	0.3	4.0	20.8	0.3	1.7	10.8	0.271
CD41b	39.5	63.0	111.4	43.2	81.9	122.1	0.234
CD42a	87.7	295.6	640.2	147.2	389.5	612.1	0.362
CD24	8.0	26.1	51.2	3.0	14.9	47.3	0.138
CD86	3.9	27.7	57.0	1.6	14.8	34.6	0.127
CD44	11.9	16.8	46.0	8.8	19.7	43.7	0.938
CD326	3.0	17.9	64.0	0.6	8.8	35.4	0.105
CD133/1	7.7	40.0	67.1	4.4	20.1	55.6	0.052
CD29	45.1	101.6	165.5	46.4	78.4	121.7	0.141
CD69	12.5	30.3	72.9	10.7	25.3	50.2	0.223
CD142	2.8	21.0	46.0	2.4	17.7	40.5	0.962
CD45	3.1	12.8	46.5	0.7	6.6	45.0	0.301
CD31	19.3	34.9	73.9	32.3	49.7	79.0	0.059
CD20	4.4	21.1	64.0	1.3	17.5	42.0	0.303
CD14	5.2	23.4	46.6	1.4	7.0	34.1	**0.025**

EV surface antigens in patients with OD and/or cardiac disease (n=138), after stratification for sex. Levels of EV markers are reported as MFI after normalization by mean MFI of tetraspanins, and expressed as median and interquartile range (25th-50th-75th percentiles). A $P<0.05$ was considered significant.

Table S13 – EV profiling in patients after cardiovascular event after stratification for sex

EV marker	**Female** (n=18)			**Male** (n=69)			***P*-value**
	25th	50th	75th	25th	50th	75th	
Tetraspanins	35.1	45.1	76.0	36.9	56.6	80.2	0.441
CD3	0.1	7.3	31.5	1.2	21.0	43.4	0.187
CD4	5.0	29.3	43.2	9.9	28.8	74.1	0.382
CD19	0.6	15.8	57.4	11.4	35.5	59.0	0.220
CD8	14.4	27.1	50.9	13.8	31.6	66.2	0.496
HLA-II	13.3	31.9	71.0	17.2	34.1	69.7	0.745
CD56	0.0	4.2	33.0	0.0	6.9	24.7	0.642
CD105	4.5	52.5	88.9	0.0	31.5	68.5	0.533
CD2	1.5	4.6	42.4	1.2	26.0	51.1	0.322
CD1c	3.3	30.1	50.1	3.4	19.3	50.2	0.822
CD25	0.3	9.5	46.3	3.0	17.5	42.3	0.773
CD49e	9.9	34.8	56.6	14.9	35.8	66.1	0.637
ROR1	4.3	27.1	77.5	17.2	32.7	70.7	0.463
CD209	1.8	5.7	61.9	3.3	18.7	47.3	0.667
CD9	57.2	77.7	102.5	53.2	67.7	91.4	0.300
SSEA-4	5.8	30.8	69.9	0.7	17.3	56.5	0.444
HLA-I	37.6	66.8	106.1	18.1	44.6	70.0	**0.047**
CD63	49.3	78.8	127.3	58.2	94.4	117.3	0.737
CD40	48.9	91.9	196.2	47.7	82.4	119.5	0.444
CD62P	112.8	342.5	780.4	168.9	421.0	973.0	0.496
CD11c	6.6	27.7	68.0	4.4	28.9	72.8	0.883
CD81	66.4	118.5	174.6	79.3	105.4	162.4	0.496
MCSP	10.9	23.4	44.9	4.2	22.2	43.1	0.737
CD146	0.9	1.4	11.8	1.8	8.9	22.6	0.072
CD41b	93.8	129.4	302.0	85.9	144.7	325.9	0.875
CD42a	154.8	374.4	989.5	271.7	543.5	1165.1	0.292
CD24	11.0	33.8	68.6	6.3	30.5	55.0	0.457
CD86	3.4	43.4	88.5	6.7	23.8	43.1	0.240
CD44	7.8	43.4	90.4	9.8	37.1	79.2	0.714
CD326	2.7	23.7	43.5	1.9	16.1	43.3	0.900
CD133/1	5.3	30.8	55.1	7.5	28.2	54.7	0.996
CD29	75.0	109.5	321.2	61.2	104.8	157.8	0.516
CD69	22.1	62.7	84.7	25.6	39.0	71.7	0.163
CD142	2.9	19.4	47.9	4.1	26.6	47.2	0.896
CD45	10.2	28.1	51.8	10.5	27.2	53.2	0.950
CD31	45.9	74.4	100.7	53.2	82.9	115.9	0.236
CD20	8.8	20.8	68.6	5.7	17.8	48.0	0.473
CD14	0.6	13.6	31.1	5.8	21.3	53.1	0.205

EV surface antigens in patients with an acute CV event (n=87), after stratification for sex. Levels of EV markers are reported as MFI after normalization by mean MFI of tetraspanins, and expressed as median and interquartile range (25th-50th-75th percentiles). A P<0.05 was considered significant.

Table S14 – Assessment of EV biomarkers at unadjusted and sex-adjusted regressions

EV marker		Unadjusted Analysis			Sex-adjusted Analysis		
		OR	95% CI	*P*-value	OR	95% CI	*P*-value
**CV Risk Factor**	CD40	1.002	0.998-1.006	0.300	-	-	-
	CD86	1.015	1.005-1.026	**0.003**	1.015	1.007-1.024	**0.004**
	CD44	0.997	0.991-1.002	0.247	-	-	-
	CD133/1	1.007	1.001-1.014	**0.047**	1.007	1.000-1.014	0.061
	CD20	1.009	0.999-1.019	0.090	-	-	-
**OD & Cardiac Disease**	CD40	1.011	1.004-1.018	**0.001**	1.012	1.005-1.019	**<0.001**
	CD86	1.022	1.011-1.033	**<0.001**	1.026	1.014-1.038	**<0.001**
	CD44	0.998	0.991-1.006	0.627	-	-	-
	CD133/1	1.012	1.004-1.019	**0.002**	1.015	1.007-1.023	**<0.001**
	CD20	1.022	1.011-1.034	**<0.001**	1.025	1.013-1.036	**<0.001**
**Acute CV Event**	CD40	1.021	1.013-1.028	**<0.001**	1.023	1.015-1.031	**<0.001**
	CD86	1.029	1.017-1.042	**<0.001**	1.037	1.023-1.052	**<0.001**
	CD44	1.011	1.004-1.018	**0.003**	1.013	1.006-1.021	**0.001**
	CD133/1	1.013	1.004-1.021	**0.003**	1.017	1.008-1.027	**<0.001**
	CD20	1.024	1.012-1.037	**<0.001**	1.028	1.015-1.042	**<0.001**

Univariate and sex-adjusted multivariate logistic regressions to assess association between EV antigens differentially expressed in healthy controls after stratification for sex (see Table S10) and presence of a CV risk factors, or OD/cardiac disease, or an acute CV event. The group of healthy controls was used as comparator. The table reports odds ratio (OR) and 95% confidence interval (95% CI); *P*<0.05 was considered significant and showed in bold. An OR greater than 1 indicates an increased likelihood of a CV risk factor, OD/cardiac disease, or an acute CV event, and an OR less than 1 a decreased likelihood.

Table S15 – Assessment of EV biomarkers by analysis of ROC curves stratified for sex

EV marker		Female			Male		
		AUC	95% CI	*P*-value	AUC	95% CI	*P*-value
**CV Risk Factor**	CD40	0.518*	0.435-0.601	0.662	0.505	0.414-0.596	0.907
	CD86	0.583	0.502-0.633	**0.049**	0.606	0.522-0.690	**0.020**
	CD44	0.544*	0.460-0.629	0.290	0.544*	0.456-0.633	0.328
	CD133/1	0.518	0.436-0.600	0.667	0.634	0.551-0.717	**0.003**
	CD20	0.527	0.447-0.606	0.524	0.537	0.453-0.622	0.414
**OD & Cardiac Disease**	CD40	0.569	0.463-0.676	0.212	0.724	0.641-0.806	**<0.001**
	CD86	0.643	0.535-0.752	**0.010**	0.662	0.578-0.747	**0.001**
	CD44	0.525*	0.418-0.632	0.657	0.593	0.501-0.685	**0.049**
	CD133/1	0.634	0.528-0.739	**0.016**	0.630	0.544-0.717	**0.006**
	CD20	0.620	0.508-0.732	**0.031**	0.652	0.567-0.738	**0.001**
**Acute CV Event**	CD40	0.775	0.644-0.905	**<0.001**	0.809	0.732-0.885	**<0.001**
	CD86	0.667	0.501-0.836	**0.030**	0.745	0.660-0.830	**<0.001**
	CD44	0.618	0.467-0.769	0.123	0.674	0.582-0.766	**0.001**
	CD133/1	0.568	0.414-0.722	0.379	0.710	0.619-0.801	**<0.001**
	CD20	0.666	0.508-0.824	**0.031**	0.712	0.624-0.801	**<0.001**

Analyses of ROC curves were performed to evaluate diagnostic performance of EV antigens in the discrimination of CV risk factors, or OD/cardiac disease, or acute CV events, from healthy subjects, used as controls. The table reports the area under the curve (AUC) and 95% confidence interval (95% CI) after stratification for sex. $P<0.05$ was considered significant and showed in bold (*AUC computed for threshold less than X).

Table S16 – Assessment of EV biomarkers at age and sex-adjusted regressions

EV marker		Age-adjusted Analysis			Sex-adjusted Analysis			Age & Sex-adjusted Analysis		
		OR	95% CI	*P*-value	OR	95% CI	*P*-value	OR	95% CI	*P*-value
**CV Risk Factor**	Tetraspanins	0.993	0.988-0.999	**0.018**	-	-	-	0.994	0.988-1.001	0.071
	CD8	1.011	0.999-1.020	0.054	-	-	-	-	-	-
	HLA-II	1.010	1.004-1.017	**0.002**	-	-	-	1.010	1.003-1.018	**0.003**
	CD49e	-	-	-	-	-	-	-	-	-
	HLA-I	0.995	0.992-0.1.004	0.061	-	-	-	-	-	-
	CD40	-	-	-	-	-	-	-	-	-
	CD62P	1.001	1.002-1.005	**0.006**	-	-	-	1.001	0.999-1.001	0.104
	CD11c	1.011	1.001-1.021	**0.028**	-	-	-	1.009	1.001-1.019	**0.034**
	CD41b	1.003	1.001-1.010	**0.046**	-	-	-	0.998	0.996-1.001	0.051
	CD42a	-	-	-	-	-	-	-	-	-
	CD86	-	-	-	1.015	1.007-1.024	**0.004**	1.009	0.996-1.027	0.073
	CD44	-	-	-	-	-	-	-	-	-
	CD133/1	-	-	-	1.007	1.000-1.014	0.061	-	-	-
	CD31	1.003	1.001-1.007	**0.008**	-	-	-	1.002	1.001-1.006	**0.001**
	CD20	-	-	-	-	-	-	-	-	-
**OD & Cardiac Disease**	Tetraspanins	-	-	-	-	-	-	-	-	-
	CD8	1.015	0.997-1.023	0.071	-	-	-	-	-	-
	HLA-II	1.017	1.008-1.025	**<0.001**	-	-	-	1.016	1.009-1.026	**<0.001**
	CD49e	1.035	1.022-1.049	**<0.001**	-	-	-	1.036	1.023-1.050	**<0.001**
	HLA-I	0.998	0.995-1.000	0.098	-	-	-	-	-	-
	CD40	1.009	0.993-1.016	0.077	1.012	1.005-1.019	**<0.001**	1.008	0.999-1.016	0.054
	CD62P	1.002	1.001-1.003	**0.008**	-	-	-	1.001	1.001-1.003	**0.006**
	CD11c	1.026	0.991-1.037	0.101	-	-	-	-	-	-
	CD41b	-	-	-	-	-	-	-	-	-
	CD42a	1.002	1.001-1.003	**0.005**	-	-	-	1.001	1.001-1.002	**0.004**
	CD86	-	-	-	1.026	1.014-1.038	**<0.001**	1.012	0.997-1.026	0.097
	CD44	-	-	-	-	-	-	-	-	-
	CD133/1	-	-	-	1.015	1.007-1.023	**<0.001**	1.009	0.993-1.021	0.145
	CD31	1.007	1.002-1.021	**0.032**	-	-	-	0.999	0.997-1.001	0.384
	CD20	-	-	-	1.025	1.013-1.036	**<0.001**	1.029	1.016-1.041	**<0.001**
**Acute CV Event**	Tetraspanins	1.025	1.015-1.035	**<0.001**	-	-	-	1.018	0.998-1.033	0.076
	CD8	1.010	1.000-1.022	0.052	-	-	-	-	-	-
	HLA-II	1.014	0.998-1.035	0.075	-	-	-	-	-	-
	CD49e	1.041	1.026-1.056	**<0.001**	-	-	-	1.043	1.026-1.060	**<0.001**
	HLA-I	-	-	-	-	-	-	-	-	-
	CD40	1.011	0.998-1.026	0.064	1.023	1.015-1.031	**<0.001**	1.011	1.000-1.024	0.058
	CD62P	1.002	1.001-1.003	**0.001**	-	-	-	1.001	1.001-1.002	**0.001**
	CD11c	1.015	0.990-1.047	0.096	-	-	-	-	-	-
	CD41b	1.005	1.003-1.007	**<0.001**	-	-	-	1.005	1.000-1.008	0.053
	CD42a	1.002	1.001-1.003	**<0.001**	-	-	-	1.002	1.001-1.002	**<0.001**
	CD86	-	-	-	1.037	1.023-1.052	**<0.001**	1.018	0.992-1.053	0.165
	CD44	1.007	0.999-1.014	0.062	1.013	1.006-1.021	**0.001**	1.009	1.001-1.018	**0.026**
	CD133/1	-	-	-	1.017	1.008-1.027	**<0.001**	1.021	0.990-1.032	0.283
	CD31	1.004	1.001-1.009	**0.024**	-	-	-	1.003	1.001-1.006	**0.003**
	CD20	-	-	-	1.028	1.015-1.042	**<0.001**	1.016	0.997-1.034	0.075

Age- and sex- adjusted multivariate logistic regressions to assess association between EV antigens differentially expressed in healthy controls and presence of a CV risk factors, or OD/cardiac disease, or an acute CV event. Healthy controls were used as comparator (see also Tables S7-S14).

Table S17 – Assessment of EV biomarkers by analysis of ROC curves stratified for age and sex

	EV marker	< 40y			40-49y			50-59y			60-69y			70y and over		
		AUC	95% CI	*P*-value	AUC	95% CI	*P*-value	AUC	95% CI	*P*-value	AUC	95% CI	*P*-value	AUC	95% CI	*P*-value
**CV Risk Factor**	**Female**	HC=20; CV-RF=40			HC=15; CV-RF=28			HC=16; CV-RF=39			HC=9; CV-RF=29			HC=9; CV-RF=18		
	HLA-II	0.571*	0.491-0.723	0.371	0.564*	0.384-0.744	0.491	0.663	0.520-0.807	0.059	0.720	0.563-0.878	**0.048**	0.877	0.737-1.000	**0.002**
	CD11c	0.523*	0.374-0.672	0.772	0.638*	0.458-0.818	0.139	0.616	0.449-0.783	0.179	0.586	0.369-0.777	0.440	0.793	0.591-0.995	**0.015**
	CD31	0.791*	0.674-0.909	**<0.001**	0.529*	0.357-0.700	0.760	0.540	0.387-0.693	0.643	0.680	0.494-0.866	0.107	1.000	0.999-1.000	**<0.001**
	**Male**	HC=12; CV-RF=19			HC=11; CV-RF=21			HC=22; CV-RF=38			HC=11; CV-RF=15			HC=7; CV-RF=21		
	HLA-II	0.711*	0.528-0.893	0.052	0.641	0.436-0.846	0.197	0.699	0.567-0.830	**0.011**	0.739	0.528-0.951	**0.040**	0.748	0.573-0.924	0.053
	CD11c	0.715*	0.535-0.895	**0.047**	0.667	0.479-0.855	0.127	0.539	0.392-0.687	0.613	0.521	0.295-0.747	0.856	0.738	0.508-0.969	0.063
	CD31	0.930*	0.840-1.000	**<0.001**	0.606*	0.402-0.810	0.331	0.612*	0.472-0.753	0.149	0.624*	0.403-0.845	0.287	0.874	0.744-1.000	**0.004**
**OD & Cardiac Disease**	**Female**	HC=20; OD-CD=13			HC=15; OD-CD=5			HC=16; OD-CD=9			HC=9; OD-CD=6			HC=9; OD-CD=12		
	HLA-II	0.554	0.335-0.772	0.606	0.720	0.440-1.000	0.150	0.625	0.360-0.890	0.308	0.630	0.293-0.966	0.409	0.944	0.845-1.000	**0.001**
	CD49e	0.723	0.505-0.941	**0.033**	0.693	0.423-0.963	0.206	0.611	0.380-0.843	0.365	0.833	0.619-1.000	**0.034**	0.926	0.815-1.000	**0.001**
	CD62P	0.831*	0.679-0.982	**0.002**	0.573	0.298-0.849	0.631	0.750	0.549-0.951	**0.042**	1.000	0.999-1.000	**0.001**	0.991	0.961-1.000	**<0.001**
	CD42a	0.769*	0.601-0.938	**0.010**	0.680	0.448-0.912	0.239	0.785	0.597-0.972	**0.020**	0.907	0.726-1.000	**0.010**	1.000	0.999-1.000	**<0.001**
	CD20	0.785	0.608-0.961	**0.006**	0.613	0.298-0.928	0.458	0.510*	0.246-0.775	0.932	0.509*	0.199-0.819	0.953	0.778	0.553-1.000	**0.033**
	**Male**	HC=12; OD-CD=4			HC=11; OD-CD=15			HC=22; OD-CD=30			HC=11; OD-CD=21			HC=7; OD-CD=23		
	HLA-II	0.604*	0.285-0.924	0.544	0.624	0.393-0.855	0.287	0.609	0.456-0.762	0.182	0.519	0.315-0.724	0.858	0.820	0.670-0.970	**0.012**
	CD49e	0.625	0.316-0.934	0.467	0.642	0.426-0.859	0.223	0.706	0.566-0.846	**0.012**	0.779	0.601-0.957	**0.010**	0.807	0.630-0.985	**0.015**
	CD62P	0.958*	0.862-1.000	**0.008**	0.570	0.345-0.794	0.551	0.818	0.703-0.933	**<0.001**	0.766	0.570-0.963	**0.015**	0.994	0.974-1.000	**<0.001**
	CD42a	0.771*	0.533-1.000	0.115	0.527	0.290-0.765	0.815	0.727	0.587-0.867	**0.005**	0.810	0.662-0.957	**0.005**	0.975	0.927-1.000	**<0.001**
	CD20	0.688	0.315-1.000	0.275	0.697	0.488-0.906	0.092	0.729	0.587-0.870	**0.005**	0.615	0.420-0.810	0.293	0.556	0.343-0.769	0.659
**Acute CV Event**	**Female**	HC=20; CV-E=1			HC=15; CV-E=1			HC=16; CV-E=2			HC=9; CV-E=3			HC=9; CV-E=11		
	CD49e	1.000*	0.999-1.000	0.099	0.933	0.807-1.000	0.159	0.547*	0.134-0.960	0.833	0.926	0.759-1.000	**0.033**	0.926	0.882-1.000	**0.033**
	CD62P	0.850*	0.694-1.000	0.248	1.000*	0.999-1.000	0.104	0.813*	0.613-1.00	0.160	0.889	0.667-1.000	0.052	0.889	0.821-1.000	0.052
	CD42a	0.500	0.281-0.719	1.000	0.600*	0.352-0.848	0.745	0.719	0.302-1.000	0.325	1.000	0.999-1.000	**0.013**	1.000	0.929-1.000	**0.013**
	CD44	0.750*	0.560-0.940	0.409	0.600*	0.352-0.848	0.745	0.594	0.094-1.000	0.673	0.704	0.408-0.999	0.309	0.704	0.350-0.862	0.309
	CD31	0.950*	0.854-1.000	0.137	0.800*	0.598-1.000	0.329	1.000	0.999-1.000	**0.025**	1.000	0.999-1.000	**0.013**	1.000	0.999-1.000	**0.013**
	**Male**	HC=12; CV-E=5			HC=11; CV-E=9			HC=22; CV-E=23			HC=11; CV-E=17			HC=7; CV-E=15		
	CD49e	0.633	0.340-0.927	0.399	0.576	0.312-0.839	0.569	0.877	0.776-0.979	**<0.001**	0.746	0.557-0.935	**0.030**	0.905	0.770-1.000	**0.003**
	CD62P	0.967*	0.887-1.000	**0.003**	0.788	0.583-0.993	**0.030**	0.818	0.694-0.942	**<0.001**	0.706	0.501-0.910	0.070	0.967	0.895-1.000	**0.001**
	CD42a	0.633*	0.350-0.917	0.399	0.818	0.632-1.000	**0.017**	0.854	0.741-0.966	**<0.001**	0.963	0.899-1.000	**<0.001**	0.971	0.906-1.000	**<0.001**
	CD44	0.508	0.165-0.852	0.958	0.636	0.388-0.885	0.305	0.751	0.603-0.899	**0.004**	0.604	0.382-0.827	0.359	0.686	0.456-0.915	0.169
	CD31	0.933*	0.815-1.000	**0.006**	0.808	0.610-1.000	**0.020**	0.921	0.841-1.000	**<0.001**	0.952	0.876-1.000	**<0.001**	0.952	0.857-1.000	**0.001**

Analyses of ROC curves were performed to evaluate diagnostic performance of EV antigens in the discrimination of CV risk factors, or OD/cardiac disease, or acute CV events, from healthy subjects, used as controls. The table reports the area under the curve (AUC) and 95% confidence interval (95% CI) after stratification for sex and age. P<0.05 was considered significant and showed in bold. Only EV markers associated to CV risk indicators were included in these analysis (see Table S16) (*AUC computed for threshold less than X).

Table S18 – Correlation between Leucocytes/CRP and Age

**Spearman Rho [*P*-value]**	**Overall Cohort** (n=220)	**Healthy Controls** (n=39)	**CV Risk Factor** (n=68)	**Organ Damage** (n=55)	**Acute CV Event** (n=58)
WBC (n/L*1000)	-0.050<br>0.462	0.049<br>0.768	-0.202<br>0.098	-0.010<br>0.945	-0.199<br>0.135
Neutrophils (n/L*1000)	-0.002<br>0.975	0.047<br>0.777	-0.179<br>0.143	0.074<br>0.589	-0.119<br>0.373
Lymphocytes (n/L*1000)	-0.157<br>0.020	0.028<br>0.864	-0.100<br>0.417	-0.278<br>0.040	-0.265<br>0.044
Monocytes (n/L*1000)	-0.032<br>0.636	-0.084<br>0.611	0.012<br>0.920	0.066<br>0.630	-0.194<br>0.145
Basophils (n/L*1000)	-0.084<br>0.216	-0.140<br>0.394	-0.050<br>0.683	-0.274<br>0.043	0.015<br>0.909
Eosinophils (n/L*1000)	-0.049<br>0.470	-0.139<br>0.398	-0.006<br>0.960	-0.045<br>0.746	0.001<br>0.999
C-reactive Protein (mg/L)	0.349<br><0.001	0.724<br><0.001	0.148<br>0.091	0.226<br>0.150	0.056<br>0.717

Correlations between age and Leucocytes were evaluated by Spearman's Rho test in the overall cohort, and among healthy controls, subjects with CV risk factors, organ damage, or with an acute CV event. Rho coefficient (above) and *P*-values (below) are reported for each comparison. $P<0.05$ was considered significant and highlighted in red.

Table S19 – Correlation between EV profiling (lymphocytes/inflammation markers) and CRP

**Spearman Rho [*P*-value]**	**Overall Cohort** (n=625)	**Healthy Controls** (n=132)	**CV Risk Factor** (n=268)	**Organ Damage** (n=138)	**Acute CV Event** (n=87)
CD2	0.180<br>0.003	0.261<br>0.057	0.076<br>0.387	0.186<br>0.237	-0.133<br>0.385
CD3	0.098<br>0.105	0.102<br>0.462	0.079<br>0.372	-0.062<br>0.695	-0.107<br>0.484
CD4	0.116<br>0.056	0.237<br>0.085	0.019<br>0.826	0.089<br>0.575	-0.130<br>0.396
CD8	-0.034<br>0.576	-0.342<br>0.011	0.040<br>0.647	0.092<br>0.560	-0.098<br>0.524
CD14	0.106<br>0.082	0.088<br>0.528	0.096<br>0.277	0.044<br>0.782	0.053<br>0.729
CD69	0.042<br>0.495	-0.131<br>0.343	0.006<br>0.942	-0.213<br>0.175	0.016<br>0.917
CD86	-0.025<br>0.684	-0.377<br>0.005	0.056<br>0.526	0.025<br>0.875	-0.296<br>0.048
CD19	0.100<br>0.101	0.085<br>0.540	0.084<br>0.340	0.119<br>0.451	-0.004<br>0.978
CD20	0.089<br>0.144	-0.205<br>0.137	0.100<br>0.256	0.093<br>0.557	-0.047<br>0.762
CD24	0.132<br>0.030	-0.025<br>0.858	0.038<br>0.669	0.192<br>0.223	0.042<br>0.782
CD40	0.205<br>0.001	0.293<br>0.032	0.021<br>0.816	0.195<br>0.217	-0.149<br>0.327
CD44	0.109<br>0.072	0.082<br>0.554	0.122<br>0.167	0.055<br>0.729	-0.113<br>0.458

Correlations between C-reactive protein (mg/L) and EV profiling (markers from T-lymphocytes [CD2, CD3, CD4, CD8, CD14, CD69, CD86], B-lymphocytes [CD19, CD20, CD24], and of inflammation [CD40, CD44]) were evaluated by Spearman's Rho test in the overall cohort, and among healthy controls, subjects with CV risk factors, organ damage, or with an acute CV event. Rho coefficient (above) and *P*-values (below) are reported for each comparison. *P*<0.05 was considered significant and highlighted in red.

Table S20 – Correlation between EV profiling (lymphocytes/inflammation markers) and age

**Spearman Rho [*P*-value]**	**Overall Cohort** (n=625)	**Healthy Controls** (n=132)	**CV Risk Factor** (n=268)	**Organ Damage** (n=138)	**Acute CV Event** (n=87)
CD2	-0.083<br>0.038	0.113<br>0.195	-0.003<br>0.966	-0.071<br>0.408	0.064<br>0.553
CD3	-0.021<br>0.599	-0.032<br>0.717	-0.001<br>0.992	-0.106<br>0.217	-0.011<br>0.920
CD4	-0.015<br>0.706	0.000<br>0.999	-0.021<br>0.735	-0.136<br>0.112	0.019<br>0.859
CD8	-0.204<br><0.001	-0.237<br>0.006	-0.333<br><0.001	-0.140<br>0.103	-0.104<br>0.339
CD14	-0.135<br>0.001	-0.012<br>0.895	-0.202<br>0,001	0.240<br>0.005	-0.157<br>0.147
CD69	-0.037<br>0.357	-0.105<br>0.231	-0.132<br>0.030	-0.213<br>0.012	-0.239<br>0.026
CD86	-0.064<br>0.108	-0.194<br>0.026	-0.103<br>0.093	-0.182<br>0.033	-0.080<br>0.464
CD19	-0.067<br>0.093	-0.117<br>0.180	-0.100<br>0.101	-0.178<br>0.036	-0.079<br>0.468
CD20	-0.080<br>0.047	-0.109<br>0.214	-0.162<br>0.008	-0.217<br>0.010	0.046<br>0.628
CD24	-0.005<br>0.907	-0.052<br>0.553	-0.062<br>0.308	-0.122<br>0.153	-0.182<br>0.091
CD40	0.304<br><0.001	0.141<br>0.108	0.187<br>0.002	0.393<br><0.001	0.273<br>0.010
CD44	0.269<br><0.001	0.240<br>0.006	0.140<br>0.022	0.356<br><0.001	0.341<br>0.001

Correlations between age and EV profiling (markers from T-lymphocytes [CD2, CD3, CD4, CD8, CD14, CD69, CD86], B-lymphocytes [CD19, CD20, CD24], and of inflammation [CD40, CD44]) were evaluated by Spearman's Rho test in the overall cohort, and among healthy controls, subjects with CV risk factors, organ damage, or with an acute CV event. Rho coefficient (above) and *P*-values (below) are reported for each comparison. $P<0.05$ was considered significant and highlighted in red.

Table S21 – Correlation between EV profiling and biochemical parameters

**Spearman Rho [*P*-value]**	**WBC** (n/L*1000)	**Hb** (g/L)	**Platelets** (n/L*1000)	**eGFR** (mL/min)	**Glucose** (mmol/L)	**Tot Cholest** (mmol/L)	**HDL** (mmol/L)	**Triglycerides** (mmol/L)	**LDL** (mmol/L)
Tetraspanins	0.147<br>0.030	0.036<br>0.591	-0.015<br>0.829	0.027<br>0.504	-0.050<br>0.216	0.028<br>0.477	-0.036<br>0.368	-0.023<br>0.570	-0.014<br>0.725
CD8	0.002<br>0.975	-0.049<br>0.469	-0.020<br>0.767	-0.005<br>0.906	-0.050<br>0.216	0.03<br>0.449	-0.047<br>0.245	0.022<br>0.587	0.071<br>0.077
HLA-II	0.044<br>0.512	0.067<br>0.321	0.054<br>0.427	0.067<br>0.094	0.105<br>0.009	0.058<br>0.147	-0.012<br>0.773	0.044<br>0.268	0.050<br>0.208
CD49e	-0.014<br>0.832	-0.031<br>0.649	-0.049<br>0.468	-0.043<br>0.283	0.108<br>0.007	0.085<br>0.033	0.010<br>0.805	0.015<br>0.703	0.067<br>0.093
HLA-I	-0.048<br>0.483	-0.078<br>0.247	0.022<br>0.747	0.025<br>0.525	-0.076<br>0.056	0.028<br>0.489	-0.088<br>0.028	-0.044<br>0.276	0.004<br>0.928
CD40	0.019<br>0.774	-0.018<br>0.791	-0.004<br>0.956	-0.107<br>0.008	0.162<br><0.001	0.087<br>0.030	-0.079<br>0.049	0.002<br>0.969	0.018<br>0.659
CD62P	0.040<br>0.557	-0.004<br>0.957	0.138<br>0.041	-0.036<br>0.368	0.251<br><0.001	0.014<br>0.734	0.023<br>0.568	-0.028<br>0.481	0.019<br>0.627
CD11c	-0.022<br>0.743	-0.050<br>0.462	-0.085<br>0.210	0.039<br>0.328	0.075<br>0.060	0.070<br>0.079	0.076<br>0.057	-0.025<br>0.535	0.045<br>0.263
CD41b	0.091<br>0.181	0.029<br>0.668	-0.032<br>0.634	-0.004<br>0.925	0.067<br>0.095	0.037<br>0.362	0.063<br>0.116	-0.013<br>0.749	0.039<br>0.327
CD42a	0.008<br>0.909	-0.009<br>0.891	0.030<br>0.662	-0.031<br>0.432	0.222<br><0.001	0.015<br>0.701	-0.002<br>0.965	-0.032<br>0.418	-0.009<br>0.829
CD44	-0.004<br>0.957	-0.021<br>0.759	-0.119<br>0.078	-0.137<br>0.001	0.146<br><0.001	0.055<br>0.170	-0.111<br>0.006	0.008<br>0.840	0.009<br>0.814
CD31	0.115<br>0.088	0.069<br>0.310	0.104<br>0.122	0.121<br>0.002	0.104<br>0.009	0.042<br>0.291	-0.096<br>0.017	-0.029<br>0.467	-0.017<br>0.679

Correlations of conventional biochemical parameters (white blood cells [WBC], haemoglobin [Hb], platelets estimated glomerular filtration rate [eGFR], and gluco-lipidic profile) with EV profiling (EV surface antigens differentially expressed in healthy controls after stratitication for age) were evaluated by Spearman's Rho test in the overall cohort. Rho coefficient (above) and *P*-values (below) are reported for each comparison. *P*<0.05 was considered significant and highlighted in red.

Table S22 – Association of *EVaging* index with CV risk indicators

CV risk indicator [comparator]	*EVaging* index		
	OR	95% CI	*P*-value
CV risk factor [HC]	1.061	1.025-1.099	**0.001**
OD and/or cardiac disease [HC]	1.101	1.053-1.150	**<0.001**
Acute CV event [HC]	1.105	1.051-1.161	**<0.001**
Hypertension [others]	1.037	1.008-1.065	**0.010**
Hyperlipidemia [others]	1.024	0.998-1.050	0.072
Diabetes [others]	0.989	0.953-1.027	0.570
Obesity [others]	1.004	0.974-1.034	0.816
Chronic Kideny Disease [others]	1.031	1.008-1.054	**0.008**
ESC SCORE risk	1.148	1.042-1.255	**<0.001**

Age-adjusted univariate logistic and linear regressions to assess association of *EVaging* index with single CV risk indicators and ESC (European Society of Cardiology) SCORE risk. The table reports odds ratio (OR) and 95% confidence interval (95% CI); $P<0.05$ was considered significant and showed in bold. An OR greater than 1 indicates an increased likelihood of a CV risk indicator, and an OR less than 1 a decreased likelihood. HC, Healthy Controls; OD, Organ Damage; CV, Cardiovascular; ESC, European Society of Caridology.

Table S23 – EV profiling in patients with OD *versus* established cardiac disease

EV marker	**Organ Damage** (n=39)			**Cardiac Disease** (n=65)			**OD & Cardiac Disease** (n=34)			**Overall *P*-value**	**Pairwise Comparisons**		
	25th	50th	75th	25th	50th	75th	25th	50th	75th		**OD vs. CD**	**OD vs. OD/CD**	**CD vs. OD/CD**
Tetraspanins	4.2	12.1	22.5	8.8	14.1	22.4	9.4	15.7	47.1	0.206	-	-	-
CD3	1.4	14.0	40.1	1.3	12.0	24.1	3.0	17.1	73.8	0.402	-	-	-
CD4	6.9	17.7	60.2	0.2	16.6	30.0	6.7	34.1	108.8	**<0.001**	0.356	**<0.001**	**<0.001**
CD19	11.1	24.0	68.8	3.2	20.5	36.9	9.8	2825	70.9	0.201	-	-	-
CD8	23.5	30.9	60.5	7.8	35.6	67.4	10.5	42.0	109.7	**0.002**	0.860	**0.030**	**0.011**
HLA-II	14.1	47.9	76.1	7.8	47.3	78.8	14.3	48.3	104.6	0.562	-	-	-
CD56	0.1	5.0	17.9	0.2	3.3	9.9	0.0	9.5	34.9	0.244	-	-	-
CD105	0.0	14.9	44.3	0.2	15.1	39.9	0.0	6.9	73.0	0.971	-	-	-
CD2	1.8	6.3	37.5	1.0	6.8	22.4	1.0	13.8	70.6	0.297	-	-	-
CD1c	2.8	17.7	43.8	4.5	15.0	23.0	5.6	28.2	97.0	**<0.001**	0.154	**0.024**	**<0.001**
CD25	4.0	14.7	30.9	0.4	14.1	29.1	4.3	28.8	68.1	**0.021**	0.819	**0.022**	**0.027**
CD49e	10.6	34.4	50.0	9.1	21.1	44.6	12.2	54.1	85.3	0.079	-	-	-
ROR1	3.5	15.0	45.6	1.4	19.6	42.5	11.0	23.2	41.0	0.566	-	-	-
CD209	1.8	19.2	47.7	1.0	18.0	35.4	6.8	22.1	45.0	0.698	-	-	-
CD9	53.7	75.2	93.5	52.6	77.8	106.7	56.0	80.9	104.2	0.829	-	-	-
SSEA-4	4.2	32.0	63.7	3.3	36.7	49.3	10.0	48.8	79.5	0.079	-	-	-
HLA-I	15.1	33.6	56.5	16.4	39.1	62.7	15.3	39.9	82.4	0.907	-	-	-
CD63	64.3	91.0	138.8	89.2	118.4	165.1	70.6	108.0	130.4	0.055	-	-	-
CD40	14.6	35.1	74.3	25.8	43.8	79.4	21.5	54.8	75.7	0.056	-	-	-
CD62P	98.0	149.4	262.7	288.1	457.2	624.5	156.5	478.5	980.5	**<0.001**	**<0.001**	**0.001**	0.965
CD11c	8.2	31.1	70.5	6.1	27.3	56.9	6.0	23.6	94.3	0.894	-	-	-
CD81	72.5	118.9	167.2	40.7	99.7	143.7	53.8	109.5	161.5	0.068	-	-	-
MCSP	0.9	20.5	38.9	0.6	24.7	39.4	8.7	33.7	50.1	0.421	-	-	-
CD146	0.2	3.8	20.8	0.3	2.5	17.2	0.6	7.0	24.9	0.077	-	-	-
CD41b	44.8	69.7	104.2	32.9	72.0	120.1	49.1	87.2	129.5	0.429	-	-	-
CD42a	76.0	133.0	280.8	223.6	466.6	636.8	155.6	457.1	705.8	**<0.001**	**<0.001**	**<0.001**	0.871
CD24	5.7	26.1	52.7	2.4	29.2	47.2	13.6	35.2	72.3	0.121	-	-	-
CD86	6.0	19.3	49.0	0.5	27.6	23.8	15.1	36.9	68.8	**0.021**	0.058	**0.027**	0.081
CD44	7.0	16.0	34.3	7.8	20.0	29.9	14.0	37.7	64.5	**0.019**	0.460	**0.026**	**0.008**
CD326	2.6	15.8	63.8	1.3	14.7	45.0	4.9	17.8	52.5	0.093	-	-	-
CD133/1	14.0	42.0	80.0	12.0	41.7	65.7	15.8	56.0	87.8	0.407	-	-	-
CD29	57.9	101.3	141.9	43.6	75.4	120.0	49.0	82.3	164.3	0.284	-	-	-
CD69	18.8	36.7	71.9	8.3	39.6	64.7	23.7	46.7	69.7	0.391	-	-	-
CD142	1.1	18.9	38.8	1.7	19.3	37.2	12.7	24.5	51.7	0.605	-	-	-
CD45	2.4	16.0	41.8	0.2	14.8	36.4	5.3	16.0	56.6	0.715	-	-	-
CD31	16.9	37.1	76.8	30.1	42.6	73.5	31.2	66.7	94.2	0.135	-	-	-
CD20	8.8	25.8	63.0	1.0	28.3	56.1	11.3	35.3	64.5	0.091	-	-	-
CD14	7.0	13.4	46.0	2.4	17.5	39.5	4.4	21.4	48.1	0.411	-	-	-

EV antigens in patients with organ damage (OD) and or established cardiac disease (n=138). Levels of EV markers are reported as MFI after normalization by mean tetraspanins MFI, and expressed as median and interquartile range. A *P*<0.05 was considered significant.

Table S24 – EV profiling after stratification for pharmacological treatment

Risk Group	Antihypertensive Medications							Lipid-lowering Drugs							Hypoglycemic Agents							Antiplatelets							Anticoagulants						
	No			Yes			P-value	No			Yes			P-value	No			Yes			P-value	No			Yes			P-value	No			Yes			P-value
**CV Risk F**	*25th*	*50th*	*75th*	*25th*	*50th*	*75th*		*25th*	*50th*	*75th*	*25th*	*50th*	*75th*		*25th*	*50th*	*75th*	*25th*	*50th*	*75th*		*25th*	*50th*	*75th*	*25th*	*50th*	*75th*		*25th*	*50th*	*75th*	*25th*	*50th*	*75th*	
**Tetra**	5.2	12.3	23.1	6.8	14.2	28.9	0.065	6.1	13.2	26.3	5.3	11.5	19.0	0.140	6.1	13.1	25.6	3.4	10.3	20.0	0.100	5.6	13.2	25.9	7.0	11.7	21.0	0.357		N.A.			N.A.		N.A.
**CD8**	11.9	33.0	47.1	12.4	24.2	44.8	0.567	12.6	32.6	47.4	7.6	22.0	43.0	0.055	12.3	29.0	45.8	12.7	35.2	54.6	0.277	12.4	30.5	45.8	11.2	23.2	51.8	0.651		N.A.			N.A.		N.A.
**HLA-II**	14.7	33.2	60.1	13.4	36.6	74.5	0.435	16.1	35.9	72.2	11.8	28.4	49.9	0.064	14.2	34.2	66.7	15.0	33.0	62.0	0.931	15.8	38.2	73.6	12.8	33.1	53.0	0.063		N.A.			N.A.		N.A.
**CD49e**	1.5	10.6	25.7	3.9	9.3	21.3	0.469	1.9	9.2	26.3	3.4	10.9	18.6	0.919	1.8	9.2	24.0	5.6	12.9	42.8	0.086	2.1	10.1	24.2	1.3	10.1	29.7	0.598		N.A.			N.A.		N.A.
**HLA-I**	6.4	23.1	54.0	8.7	26.9	58.0	0.205	10.0	24.2	58.4	5.5	19.1	46.5	0.085	8.1	23.6	57.1	4.1	16.2	39.2	0.220	8.9	23.8	57.1	0.5	13.7	34.5	0.081		N.A.			N.A.		N.A.
**CD40**	10.8	24.1	56.7	11.9	25.0	49.0	0.991	9.2	24.1	51.3	12.8	27.2	62.5	0.258	9.9	24.9	55.4	16.9	22.3	51.9	0.668	9.8	24.1	51.8	16.7	34.5	62.1	0.117		N.A.			N.A.		N.A.
**CD62P**	103.7	224.1	413.8	108.5	230.8	412.6	0.779	104.4	226.3	423.5	111.0	232.3	388.1	0.963	102.9	222.0	403.9	174.6	264.8	610.6	0.094	102.9	216.2	412.9	219.4	240.2	394.0	0.211		N.A.			N.A.		N.A.
**CD11c**	1.8	12.0	25.2	1.9	11.0	33.4	0.573	1.8	11.6	31.1	2.7	11.0	22.3	0.967	1.8	11.1	26.9	0.6	17.2	37.4	0.440	1.8	11.4	29.7	1.6	12.9	24.6	0.803		N.A.			N.A.		N.A.
**CD41b**	20.8	45.5	86.0	30.0	53.1	86.3	0.105	26.9	52.4	88.5	21.4	39.0	63.0	0.115	25.9	49.4	85.6	26.3	45.6	95.2	0.856	26.3	50.5	88.6	25.8	43.8	70.9	0.251		N.A.			N.A.		N.A.
**CD42a**	87.5	152.0	343.5	100.8	156.7	248.2	0.829	98.8	154.3	290.8	83.2	170.2	368.4	0.902	88.2	154.9	294.6	100.0	143.3	320.9	0.809	87.7	153.3	294.6	112.8	161.0	302.1	0.373		N.A.			N.A.		N.A.
**CD44**	6.2	12.7	36.2	4.5	12.4	40.3	0.795	5.7	12.3	29.4	6.7	17.2	47.2	0.212	6.0	12.6	35.6	3.5	12.5	51.6	0.819	5.0	11.5	28.7	11.8	26.1	50.7	**0.002**		N.A.			N.A.		N.A.
**CD31**	10.8	23.8	53.6	15.4	25.7	46.4	0.388	12.8	25.7	52.7	15.3	21.8	45.4	0.880	13.6	24.9	51.7	5.8	25.8	54.1	0.741	13.2	28.0	53.9	13.8	22.5	38.8	0.214		N.A.			N.A.		N.A.
**OD&CD**	*25th*	*50th*	*75th*	*25th*	*50th*	*75th*	*P-value*	*25th*	*50th*	*75th*	*25th*	*50th*	*75th*	*P-value*	*25th*	*50th*	*75th*	*25th*	*50th*	*75th*	*P-value*	*25th*	*50th*	*75th*	*25th*	*50th*	*75th*	*P-value*	*25th*	*50th*	*75th*	*25th*	*50th*	*75th*	*P-value*
**Tetra**	9.1	14.6	28.6	8.3	12.9	21.4	0.314	8.8	13.8	26.7	8.2	14.2	22.3	0.867	9.2	14.2	24.9	7.1	12.0	26.9	0.601	10.7	18.9	34.8	6.2	15.2	28.9	0.102	9.0	15.0	30.7	8.2	10.0	25.7	0.053
**CD8**	8.0	24.6	57.8	13.0	28.3	80.7	0.244	10.4	28.3	80.4	9.6	21.7	60.1	0.498	8.9	22.5	48.4	15.1	49.1	106.8	**0.003**	12.1	35.6	94.9	8.0	22.3	65.0	0.060	11.7	28.2	80.8	5.2	22.7	43.7	0.113
**HLA-II**	11.8	22.7	67.9	13.0	23.7	73.2	0.554	12.5	31.8	77.6	12.9	20.1	48.3	0.455	12.6	20.0	58.8	14.4	38.5	100.3	0.059	13.0	36.2	94.0	12.2	20.0	50.4	0.069	12.8	32.5	78.3	13.2	19.5	45.7	0.175
**CD49e**	8.4	23.0	48.9	12.7	29.2	61.0	0.305	11.9	26.6	61.0	7.9	21.4	42.8	0.193	8.8	19.3	45.5	18.5	44.6	80.9	**0.003**	8.6	34.3	63.7	11.3	21.1	45.7	0.272	10.0	26.0	59.6	11.8	20.2	48.6	0.792
**HLA-I**	8.7	23.6	53.9	11.6	31.1	63.2	0.305	11.3	31.6	61.9	8.2	19.1	38.1	0.074	10.3	24.6	56.4	11.0	29.3	60.8	0.860	13.2	33.8	68.3	8.0	21.9	52.7	0.057	13.3	32.0	59.4	4.6	26.4	52.4	0.141
**CD40**	25.9	53.0	81.5	22.4	42.6	81.2	0.766	23.3	51.0	79.5	25.6	41.0	84.6	0.807	25.1	43.9	84.2	19.0	54.8	77.6	0.796	26.3	45.2	77.7	21.7	44.6	84.1	0.816	24.9	44.9	83.4	21.6	48.7	78.6	0.887
**CD62P**	142.9	350.0	613.7	159.4	308.0	603.0	0.961	146.7	308.0	610.6	165.4	410.9	601.1	0.600	174.1	329.5	585.9	107.4	323.8	702.3	0.757	130.0	290.1	593.1	178.4	370.7	629.6	0.300	132.3	286.5	588.0	299.8	474.3	647.1	**0.026**
**CD11c**	1.8	12.5	43.1	5.1	15.0	58.4	0.132	3.9	14.4	58.8	1.5	11.3	54.2	0.398	2.5	11.0	34.9	7.6	35.4	94.5	**0.002**	3.0	15.7	59.1	3.8	13.7	58.7	0.693	3.3	14.1	59.1	3.7	13.2	58.4	0.718
**CD41b**	38.3	68.1	102.4	49.7	86.0	124.2	0.051	34.7	69.7	112.0	59.7	81.9	124.9	0.171	41.1	72.5	110.7	42.1	72.4	126.3	0.857	49.1	90.1	145.9	39.4	66.1	126.8	0.069	46.6	75.4	127.6	33.2	62.2	97.1	0.072
**CD42a**	121.4	331.8	624.7	133.6	348.6	612.4	0.934	129.0	295.6	622.7	144.0	421.0	593.8	0.626	155.6	345.3	613.2	91.6	253.1	634.3	0.223	132.9	280.8	638.0	155.1	348.6	604.3	0.565	106.3	275.9	603.3	244.2	495.5	663.2	**0.024**
**CD44**	8.7	19.4	43.9	9.6	17.5	44.9	0.841	10.2	19.7	47.4	7.8	16.6	30.8	0.138	8.1	18.1	43.8	12.5	18.4	45.1	0.429	7.2	17.0	43.4	11.6	19.0	45.2	0.431	8.1	16.9	40.3	16.2	23.6	50.8	**0.028**
**CD31**	20.7	42.0	78.5	25.9	47.0	87.3	0.352	22.0	42.9	79.9	25.9	47.0	72.8	0.939	22.5	42.6	76.6	28.0	52.5	94.0	0.186	25.0	43.4	76.0	22.4	43.3	79.8	0.888	21.8	42.0	74.1	32.4	69.1	104.4	**0.016**
**CV event**	*25th*	*50th*	*75th*	*25th*	*50th*	*75th*	*P-value*	*25th*	*50th*	*75th*	*25th*	*50th*	*75th*	*P-value*	*25th*	*50th*	*75th*	*25th*	*50th*	*75th*	*P-value*	*25th*	*50th*	*75th*	*25th*	*50th*	*75th*	*P-value*	*25th*	*50th*	*75th*	*25th*	*50th*	*75th*	*P-value*
**Tetra**	35.8	56.6	76.2	32.9	54.2	82.3	0.942	36.6	54.0	78.8	29.3	54.9	95.8	0.929	33.4	52.7	77.7	43.9	72.5	126.7	0.115	38.9	64.1	85.2	26.3	45.9	72.3	0.070		N.A.			N.A.		N.A.
**CD8**	18.1	30.1	66.0	11.0	31.4	63.6	0.309	14.5	29.2	64.2	8.7	40.8	85.1	0.949	13.8	29.8	66.0	8.1	41.8	60.6	0.995	14.5	29.2	66.0	13.3	43.5	63.2	0.956		N.A.			N.A.		N.A.
**HLA-II**	13.9	34.0	65.1	16.2	41.9	75.0	0.658	15.4	33.6	66.8	14.4	51.0	88.1	0.804	16.0	34.1	68.2	7.2	38.2	81.8	0.588	17.5	37.2	69.0	12.0	26.1	77.1	0.383		N.A.			N.A.		N.A.
**CD49e**	15.4	30.7	61.0	13.9	38.9	71.7	0.512	14.9	31.5	62.6	13.6	59.1	80.6	0.226	14.0	35.2	65.3	19.4	47.2	70.7	0.273	13.7	30.3	59.6	20.8	62.3	71.1	0.071		N.A.			N.A.		N.A.
**HLA-I**	20.0	43.0	80.9	25.7	54.9	85.9	0.472	26.5	46.1	79.1	16.7	58.2	96.0	0.913	20.0	45.3	80.9	40.7	59.8	95.4	0.116	21.5	44.1	66.3	28.5	71.2	113.3	0.054		N.A.			N.A.		N.A.
**CD40**	45.8	79.3	144.4	49.8	92.8	125.4	0.529	48.7	84.1	144.8	46.3	86.0	114.2	0.725	45.8	85.2	130.5	71.8	89.2	174.0	0.526	45.4	79.3	115.9	68.9	113.6	249.9	0.060		N.A.			N.A.		N.A.
**CD62P**	134.1	400.5	948.7	190.7	392.2	953.1	0.721	143.9	408.7	895.3	165.1	390.5	911.2	0.921	138.5	314.5	848.3	401.4	700.3	997.6	**0.029**	129.2	285.0	896.7	264.4	783.1	999.2	0.059		N.A.			N.A.		N.A.
**CD11c**	4.5	22.6	68.2	7.1	32.0	78.8	0.592	6.7	29.1	68.0	3.5	28.9	83.8	0.851	5.9	28.9	71.5	4.1	28.8	92.5	0.815	5.3	26.2	68.5	10.4	35.0	80.9	0.519		N.A.			N.A.		N.A.
**CD41b**	87.2	172.2	355.7	85.3	128.7	199.1	0.138	73.2	126.1	277.0	118.0	176.2	402.2	0.122	82.5	134.5	294.1	112.6	177.2	348.2	0.263	72.3	123.4	245.9	142.3	205.2	448.2	**0.004**		N.A.			N.A.		N.A.
**CD42a**	323.0	583.2	784.3	210.0	406.9	971.7	0.242	215.2	486.6	803.4	277.1	543.5	854,2	0.552	231.8	470.8	981.0	295.0	785.5	962,4	0.350	202.7	450.2	834.1	297.7	640.9	943.0	0.346		N.A.			N.A.		N.A.
**CD44**	9.8	35.4	68.4	8.7	39.3	90.2	0.426	8.1	36.3	75.6	22.6	41.0	87.1	0.226	9.8	37.1	78.9	5.3	39.6	92.0	0.825	8.2	35.2	68.2	29.6	81.4	102.0	**0.036**		N.A.			N.A.		N.A.
**CD31**	48.3	75.2	105.4	58.0	85.4	115.3	0.443	44.5	75.9	111.0	63.6	98.9	115.9	0.215	46.4	76.5	110.9	70.3	95.1	172.9	0.124	45.9	75.9	116.5	61.1	82.9	108.2	0.882		N.A.			N.A.		N.A.

EV surface antigens in patients with or without pharmacological treatment (antihypertensive medicagtions, lipid-lowering drugs, hypoglycemic agents, anti-platelets and anticoagulants) after stratification for CV risk group (subjects with CV risk factors, or OD/cardiac disease, or acute CV events). For this analysis, we considered EV markers differentially expressed in healthy controls after stratification for age. Levels of EV markers are reported as MFI after normalization by mean MFI of tetraspanins, and expressed as median and interquartile range. A $P<0.05$ was considered significant. N.A., Not Applicable (less than 3 patients per group).

nature portfolio

Corresponding author(s): Lucio Barile

Last updated by author(s): 12.11.2024

Reporting Summary

Nature Portfolio wishes to improve the reproducibility of the work that we publish. This form provides structure for consistency and transparency in reporting. For further information on Nature Portfolio policies, see our Editorial Policies and the Editorial Policy Checklist.

Please do not complete any field with "not applicable" or n/a. Refer to the help text for what text to use if an item is not relevant to your study.
For final submission: please carefully check your responses for accuracy; you will not be able to make changes later.

Statistics

For all statistical analyses, confirm that the following items are present in the figure legend, table legend, main text, or Methods section.

n/a	Confirmed	
☐	☒	The exact sample size (*n*) for each experimental group/condition, given as a discrete number and unit of measurement
☐	☒	A statement on whether measurements were taken from distinct samples or whether the same sample was measured repeatedly
☐	☒	The statistical test(s) used AND whether they are one- or two-sided<br>*Only common tests should be described solely by name; describe more complex techniques in the Methods section.*
☐	☒	A description of all covariates tested
☐	☒	A description of any assumptions or corrections, such as tests of normality and adjustment for multiple comparisons
☐	☒	A full description of the statistical parameters including central tendency (e.g. means) or other basic estimates (e.g. regression coefficient) AND variation (e.g. standard deviation) or associated estimates of uncertainty (e.g. confidence intervals)
☒	☐	For null hypothesis testing, the test statistic (e.g. *F*, *t*, *r*) with confidence intervals, effect sizes, degrees of freedom and *P* value noted<br>*Give P values as exact values whenever suitable.*
☒	☐	For Bayesian analysis, information on the choice of priors and Markov chain Monte Carlo settings
☒	☐	For hierarchical and complex designs, identification of the appropriate level for tests and full reporting of outcomes
☒	☐	Estimates of effect sizes (e.g. Cohen's *d*, Pearson's *r*), indicating how they were calculated

Our web collection on statistics for biologists contains articles on many of the points above.

Software and code

Policy information about availability of computer code

Data collection	SPSS Statistics 26 (IBM, USA)
Data analysis	SPSS Statistics 26 (IBM, USA); Python 3.5 (scikit-learn), and GraphPad PRISM 8.0 (La Jolla, USA)

For manuscripts utilizing custom algorithms or software that are central to the research but not yet described in published literature, software must be made available to editors and reviewers. We strongly encourage code deposition in a community repository (e.g. GitHub). See the Nature Portfolio guidelines for submitting code & software for further information.

Data

Policy information about availability of data

All manuscripts must include a data availability statement. This statement should provide the following information, where applicable:

- Accession codes, unique identifiers, or web links for publicly available datasets
- A description of any restrictions on data availability
- For clinical datasets or third party data, please ensure that the statement adheres to our policy

Data availability and code availability statements are included as requested

Research involving human participants, their data, or biological material

Policy information about studies with human participants or human data. See also policy information about sex, gender (identity/presentation), and sexual orientation and race, ethnicity and racism.

Reporting on sex and gender	Non applicable (data were stratified for sex; gender data were not available)
Reporting on race, ethnicity, or other socially relevant groupings	Non applicable
Population characteristics	Cohort characteristics are reported in the results section
Recruitment	Retrospective analyses of samples used in previous studies (recruitment 03/2017 - 07-2022)
Ethics oversight	All patients gave informed consent. Ethical committee approved single studies in which subjects were enrolled.

Note that full information on the approval of the study protocol must also be provided in the manuscript.

Field-specific reporting

Please select the one below that is the best fit for your research. If you are not sure, read the appropriate sections before making your selection.

[X] Life sciences [ ] Behavioural & social sciences [ ] Ecological, evolutionary & environmental sciences

For a reference copy of the document with all sections, see nature.com/documents/nr-reporting-summary-flat.pdf

Life sciences study design

All studies must disclose on these points even when the disclosure is negative.

Sample size	625 patients
Data exclusions	Retrospective recruitment; no data exclusion
Replication	The overall cohort was divided in a training dataset (n=468) and in a validation dataset (n=157)
Randomization	Not applicable
Blinding	Not applicable

Behavioural & social sciences study design

All studies must disclose on these points even when the disclosure is negative.

Study description	Not applicable
Research sample	Not applicable
Sampling strategy	Not applicable
Data collection	Not applicable
Timing	Not applicable
Data exclusions	Not applicable
Non-participation	Not applicable
Randomization	Not applicable

Ecological, evolutionary & environmental sciences study design

All studies must disclose on these points even when the disclosure is negative.

Study description	Not applicable
Research sample	Not applicable
Sampling strategy	Not applicable
Data collection	Not applicable
Timing and spatial scale	Not applicable
Data exclusions	Not applicable
Reproducibility	Not applicable
Randomization	Not applicable
Blinding	Not applicable

Did the study involve field work? ☐ Yes ☒ No

Field work, collection and transport

Field conditions	Not applicable
Location	Not applicable
Access & import/export	Not applicable
Disturbance	Not applicable

Reporting for specific materials, systems and methods

We require information from authors about some types of materials, experimental systems and methods used in many studies. Here, indicate whether each material, system or method listed is relevant to your study. If you are not sure if a list item applies to your research, read the appropriate section before selecting a response.

Materials & experimental systems

n/a	Involved in the study	
☐	☒	Antibodies
☒	☐	Eukaryotic cell lines
☒	☐	Palaeontology and archaeology
☒	☐	Animals and other organisms
☐	☒	Clinical data
☒	☐	Dual use research of concern
☒	☐	Plants

Methods

n/a	Involved in the study	
☒	☐	ChIP-seq
☐	☒	Flow cytometry
☒	☐	MRI-based neuroimaging

Antibodies

Antibodies used	MACSPlex EV Kit IO human, Miltenyi Biotec
Validation	Standardized validated commercially available kit including analyses for 37 different EV antigens

Eukaryotic cell lines

Policy information about cell lines and Sex and Gender in Research

Cell line source(s)	Not applicable
Authentication	Not applicable
Mycoplasma contamination	Not applicable
Commonly misidentified lines (See ICLAC register)	Not applicable

Palaeontology and Archaeology

Specimen provenance	Not applicable
Specimen deposition	Not applicable
Dating methods	Not applicable

☐ Tick this box to confirm that the raw and calibrated dates are available in the paper or in Supplementary Information.

Ethics oversight	Not applicable

Note that full information on the approval of the study protocol must also be provided in the manuscript.

Animals and other research organisms

Policy information about studies involving animals; ARRIVE guidelines recommended for reporting animal research, and Sex and Gender in Research

Laboratory animals	Not applicable
Wild animals	Not applicable
Reporting on sex	Not applicable
Field-collected samples	Not applicable
Ethics oversight	Not applicable

Note that full information on the approval of the study protocol must also be provided in the manuscript.

Clinical data

Policy information about clinical studies
All manuscripts should comply with the ICMJE guidelines for publication of clinical research and a completed CONSORT checklist must be included with all submissions.

Clinical trial registration	This is not a clinical trial.
Study protocol	Not applicable
Data collection	Retrospective
Outcomes	CV risk profile / CV fatal events / Prediction of chronological age

Dual use research of concern

Policy information about dual use research of concern

Hazards

Could the accidental, deliberate or reckless misuse of agents or technologies generated in the work, or the application of information presented in the manuscript, pose a threat to:

No	Yes	
X	☐	Public health
X	☐	National security
X	☐	Crops and/or livestock
X	☐	Ecosystems
X	☐	Any other significant area

Experiments of concern

Does the work involve any of these experiments of concern:

No	Yes	
X	☐	Demonstrate how to render a vaccine ineffective
X	☐	Confer resistance to therapeutically useful antibiotics or antiviral agents
X	☐	Enhance the virulence of a pathogen or render a nonpathogen virulent
X	☐	Increase transmissibility of a pathogen
X	☐	Alter the host range of a pathogen
X	☐	Enable evasion of diagnostic/detection modalities
X	☐	Enable the weaponization of a biological agent or toxin
X	☐	Any other potentially harmful combination of experiments and agents

Plants

Seed stocks: Not applicable

Novel plant genotypes: Not applicable

Authentication: Not applicable

ChIP-seq

Data deposition Not applicable

☐ Confirm that both raw and final processed data have been deposited in a public database such as GEO.

☐ Confirm that you have deposited or provided access to graph files (e.g. BED files) for the called peaks.

Data access links
May remain private before publication.: Not applicable

Files in database submission: Not applicable

Genome browser session (e.g. UCSC): Not applicable

Methodology

Replicates: Not applicable

Sequencing depth: Not applicable

Antibodies: Not applicable

Peak calling parameters: Not applicable

Data quality: Not applicable

Software	Not applicable

Flow Cytometry

Plots

Confirm that:

Please consider that plots are not reported in the present manuscript, but representative plots are available if requested

- [X] The axis labels state the marker and fluorochrome used (e.g. CD4-FITC).
- [X] The axis scales are clearly visible. Include numbers along axes only for bottom left plot of group (a 'group' is an analysis of identical markers).
- [X] All plots are contour plots with outliers or pseudocolor plots.
- [ ] A numerical value for number of cells or percentage (with statistics) is provided.

Methodology

Sample preparation	Serial centrifugations and capture by beads covered with Ab against specific EV antigens
Instrument	MACSQuant, Miltenyi Biotec
Software	Kalusa
Cell population abundance	Not applicable
Gating strategy	Selection of beads populations capturing single EV antigens

- [X] Tick this box to confirm that a figure exemplifying the gating strategy is provided in the Supplementary Information.

Magnetic resonance imaging

Experimental design

Design type	Not applicable
Design specifications	Not applicable
Behavioral performance measures	Not applicable

Imaging type(s)	Not applicable
Field strength	Not applicable
Sequence & imaging parameters	Not applicable
Area of acquisition	Not applicable

Diffusion MRI [ ] Used [ ] Not used

Preprocessing

Preprocessing software	Not applicable
Normalization	Not applicable
Normalization template	Not applicable
Noise and artifact removal	Not applicable
Volume censoring	Not applicable

Statistical modeling & inference

Model type and settings	Not applicable
Effect(s) tested	Not applicable

Specify type of analysis: ☐ Whole brain ☐ ROI-based ☐ Both

Statistic type for inference (See Eklund et al. 2016)	Not applicable
Correction	Not applicable

Models & analysis

n/a	Involved in the study
☒	☐ Functional and/or effective connectivity
☒	☐ Graph analysis
☒	☐ Multivariate modeling or predictive analysis

Functional and/or effective connectivity	Not applicable
Graph analysis	Not applicable
Multivariate modeling and predictive analysis	Not applicable

This checklist template is licensed under a Creative Commons Attribution 4.0 International License, which permits use, sharing, adaptation, distribution and reproduction in any medium or format, as long as you give appropriate credit to the original author(s) and the source, provide a link to the Creative Commons license, and indicate if changes were made. The images or other third party material in this article are included in the article's Creative Commons license, unless indicated otherwise in a credit line to the material. If material is not included in the article's Creative Commons license and your intended use is not permitted by statutory regulation or exceeds the permitted use, you will need to obtain permission directly from the copyright holder. To view a copy of this license, visit http://creativecommons.org/licenses/by/4.0/
